# Challenges and opportunities for the diverse substrates of SPOP E3 ubiquitin ligase in cancer

**DOI:** 10.7150/thno.113356

**Published:** 2025-05-08

**Authors:** Xiaojuan Yang, Jiang Zhu, Xue Tao, Fengwei Gao, Yunshi Cai, Yinghao Lv, Sinan Xie, Kunlin Xie, Tian Lan, Junhong Han, Hong Wu

**Affiliations:** 1 Liver Digital Transformation Research Laboratory, State Key Laboratory of Biotherapy and Cancer Center, West China Hospital, Sichuan University and Collaborative Innovation Center of Biotherapy, Chengdu, Sichuan 610041, P.R. China; 2 Department of Biotherapy, Cancer Center and State Laboratory of Biotherapy, and Frontiers Science Center for Disease-related Molecular Network, West China Hospital, Sichuan University, Chengdu, 610041, China.; 3 Liver Transplantation Center, Liver Digital Transformation Research Laboratory, State Key Laboratory of Biotherapy and Cancer Center, West China Hospital, Sichuan University and Collaborative Innovation Center of Biotherapy, Chengdu, Sichuan 610041, P.R. China; 4 Breast Center, Department of General Surgery, West China Hospital, Sichuan University, Chengdu, China

**Keywords:** SPOP, diverse substrates, functions, cancer, therapeutic targeting

## Abstract

The Speckle-type POZ protein (SPOP), a substrate adaptor of the cullin-RING E3 ligase complex, mediates both the degradation and non-degradative ubiquitination of substrates, which are crucial for regulating various biological functions and cellular processes. Dysregulation of SPOP-mediated ubiquitination has been implicated in several cancers. Emerging evidence suggests that SPOP functions as a double-edged sword: acting as a tumor suppressor in prostate cancer (PCa), hepatocellular carcinoma (HCC), and colorectal cancer (CRC), while potentially serving as an oncoprotein in kidney cancer (KC). Therefore, SPOP's role in tumorigenesis appears to be tissue- or context-dependent. Numerous downstream substrates of SPOP have been identified across various cancers, where they regulate carcinogenesis, metabolic reprogramming, cell death, immune evasion, therapy resistance, and tumor microenvironment (TME) remodeling. However, the definitive role of SPOP in these cancers requires further investigation. A comprehensive understanding of the molecular mechanisms of SPOP in different cancer types will provide new insights into its function in oncogenesis, potentially advancing anti-cancer drug development. Here, we summarize the latest findings on SPOP's functions and structural features, its regulatory mechanisms, the roles of its substrates in various cancers, and SPOP-targeting strategies.

## 1. Introduction

**Figure 1:**
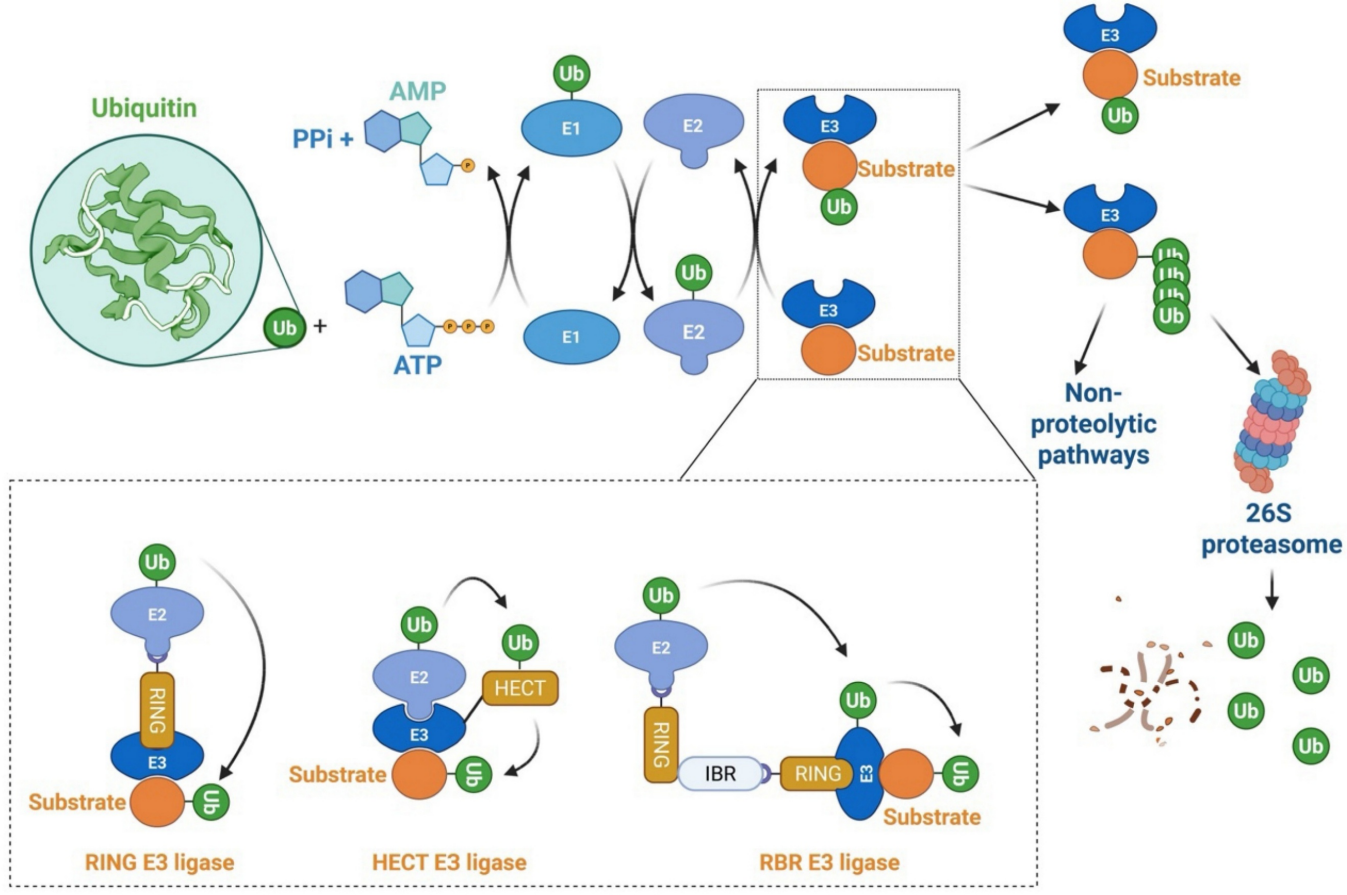
** Ubiquitination and degradation of target proteins.** This figure illustrates the process of ubiquitination, where target proteins are tagged with ubiquitin molecules, signaling their degradation by the 26S proteasome. The process begins with the activation of ubiquitin by the E1 enzyme, followed by its transfer to the E2 conjugating enzyme. The E3 ligase then facilitates the attachment of ubiquitin to the target protein, often in the form of a polyubiquitin chain, which serves as a recognition signal for the proteasome. However, when a protein is tagged with a single ubiquitin (monoubiquitination), it may not lead to degradation but instead may regulate non-proteolytic functions, such as modifying protein activity or localization. Once the polyubiquitinated protein is recognized by the proteasome, it is unfolded and translocated into the proteolytic core for degradation. HECT, Homology to E6AP C-terminus; RBR, RING homology-in-between-RING; RING: Really interesting new gene.

Proteasome-mediated protein degradation is one of the principal proteolytic pathways in eukaryotes, regulating nearly all cellular processes. This pathway, governed by the ubiquitin-proteasome system (UPS), plays a critical role in maintaining cellular homeostasis 1-4. The UPS exerts its biological functions through a series of enzymatic events, encompassing two distinct steps. In the first step, three classes of enzymes are involved: E1 (ubiquitin-activating enzymes), E2 (ubiquitin-conjugating enzymes), and E3 (ubiquitin-protein ligases), where substrate specificity is primarily determined by specific E3 ligases. The second step involves the 26S proteasome complex, which serves as the proteolytic component of the system 5-8 [Figure 1]. In humans, there are typically only two E1 enzymes, but around 40 E2 enzymes and over 600 putative E3 ligases, reflecting the complexity and specificity of substrate recognition in the UPS 4,9-11. E3 ligases are categorized into three major families: the really interesting new gene (RING) family, the homology to E6AP C-terminus (HECT) family, and the RING homology-in-between-RING (RBR) family 9,11-13. The HECT and RBR family E3 ligases catalyze the indirect transfer of ubiquitin from the E2 enzyme to a catalytic cysteine on the E3, followed by transfer to the target protein. In contrast, RING family E3 ligases mediate a direct, one-step ubiquitination, where ubiquitin is transferred from the E2 enzyme directly to the substrate 11,13,14 [Figure 1]. The RING family is the largest and most diverse group of E3 ligases, encompassing approximately 270 members 15. A canonical RING finger domain is a zinc-binding motif that contains conserved cysteine and histidine residues at specific intervals 16. This structure is essential for E2-dependent ubiquitination, facilitating the direct transfer of ubiquitin from E2 enzymes to substrate proteins, thereby ensuring precise regulation of ubiquitin-dependent cellular processes 16. The HECT family is classified into three subclasses: (1) NEDD4/NEDD4-like E3s, which include WW domains that recognize PY motifs in substrates such as ion channels 17; (2) HERC E3s, which possess RLD domains crucial for membrane association and GTPase regulation 17; and (3) non-canonical HECT E3s, such as HUWE1, which lack WW/RLD domains and regulate MYC stability 17. Genomic analysis reveals that humans encode around 30 HECT E3 genes, compared to more than 600 RING-type E3 ligases, underscoring the distinct evolutionary and functional trajectories of these two families 15. The HECT family is distinguished by catalytic flexibility via C-terminal domains that allosterically regulate ubiquitin chain formation, in contrast to RING E3s, which depend on E2 selectivity 18. RBR E3 ligases, identified through sequence alignments, exhibit a unique tripartite structure with three zinc-binding domains: two canonical RING domains (RING1 and RING2) flanking a central in-between-RING (IBR) domain 19. The RING1 domain binds to ubiquitin-charged E2 enzymes, while the RING2 domain contains a critical cysteine residue that accepts ubiquitin from the E2Ub intermediate—a mechanism typical of HECT-type E3s 20. Thus, RBR E3s combine features of both RING and HECT families, enabling efficient ubiquitin transfer.

Additionally, compensation mechanisms within ubiquitination pathways are critical for maintaining cellular homeostasis and ensuring proper protein regulation, particularly in response to disruptions in specific components of the UPS. For instance, in yeast, the dosage compensation mechanism involves a network of E3 ubiquitin ligases and N-acetyltransferases that collaborate to regulate the levels of multiprotein complex subunits by enhancing their proteolysis 21. The compensation of Pop3 and Bet4 primarily relies on the minor N-acetyltransferase NatD. Interestingly, even in the absence of NatD, canonical substrates such as histones H2A and H4 were still compensated, indicating that stoichiometric control can occur independently of N-acetylation 21. This highlights that the Ac/N-end rule pathway, while significant, is not the sole contributor to stoichiometry control, indicating a more intricate network of interactions that enable cells to adapt to fluctuations in protein levels. Furthermore, compensatory mechanisms are not limited to the UPS; they also encompass autophagy. Under conditions of nitrogen starvation, yeast fatty acid synthase (FASN) is predominantly degraded through autophagy 22. In the absence of autophagy, the UPS provides a compensatory mechanism for the degradation of FAS. Furthermore, it has identified that the degradation of Fas2 via the UPS is dependent on the E3 ubiquitin ligase known as Ubr1 22. This interplay between different degradation pathways underscores the cell's ability to maintain proteostasis and respond to various stressors, emphasizing the importance of understanding these compensatory responses in the context of diseases.

**Figure 2:**
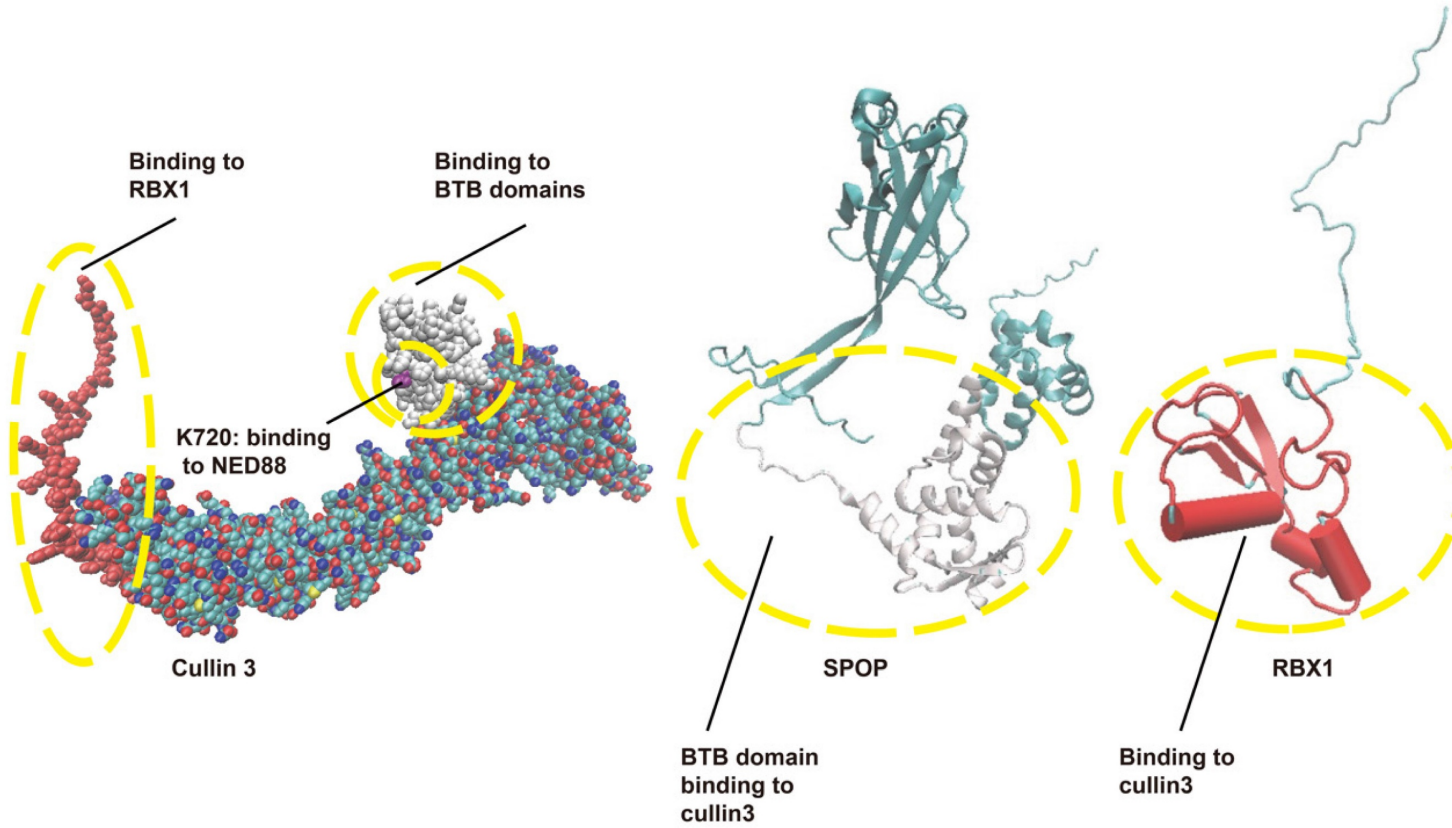
** The structure of CRL3.** CRL3 is composed of cullin 3, RBX1, and a BTB protein, with SPOP serving as an example of a BTB protein in this complex. The interaction domains are shown: red indicates the interaction between RBX1 and cullin 3, while white represents the interaction between the BTB domain and cullin 3. BTB: Bric-à-brac/Tramtrack/Broad; CRL3: Cullin-RING ligase 3; RBX1: RING-box protein 1; SPOP: Speckle-type POZ protein.

RING E3s, with the cullin-RING ligases (CRLs) being the largest known subclass, comprising eight members, including CRL1-3, CRL4A-B, CRL5, CRL7, and CRL9. Typically, CRL E3 ligases consist of a core cullin scaffold protein, a RING-box protein (RBX1/2) that recruits the E2 enzyme, a substrate receptor protein, and an adaptor protein that connects the substrate receptor to the scaffold 3,23,24. Unlike other CRL E3 ligases, CRL3 utilizes a Bric-à-brac/Tramtrack/Broad (BTB) protein, which serves as both the substrate receptor and adaptor, such as Speckle-type pox virus and zinc finger protein (SPOP), as shown in Figure 2. CRL3 also includes RBX1 for E2 recruitment and the cullin 3 scaffold protein. Additionally, a conserved lysine residue in the C-terminal domain is conjugated to NEDD8, a modification that regulates CRL3 activity 23,25 [Figure 2].

As shown in Figure 2, SPOP functions as a substrate-binding adaptor for the Cullin3 (CUL3)/RBX1 E3 ubiquitin ligase complex. SPOP, the mammalian homolog of *Drosophila* hedgehog (Hh)-induced BTB protein (Hib), plays a crucial role in development, with studies in vertebrate models showing that its gene deletion disrupts normal physiological processes 26,27. Notably, both human and plant SPOP proteins can form dimers or oligomers, underscoring the evolutionary conservation of SPOP's function. The dimerization interface is formed by the BTB and BACK domains, while the C-terminus independently promotes the assembly of higher-order oligomers that enhance substrate ubiquitination. These oligomers boost E3 ligase activity by increasing substrate avidity and facilitating the availability of the E2 ubiquitin-conjugating enzyme 28.

## 2. Structural characteristics of the SPOP protein

**Figure 3:**
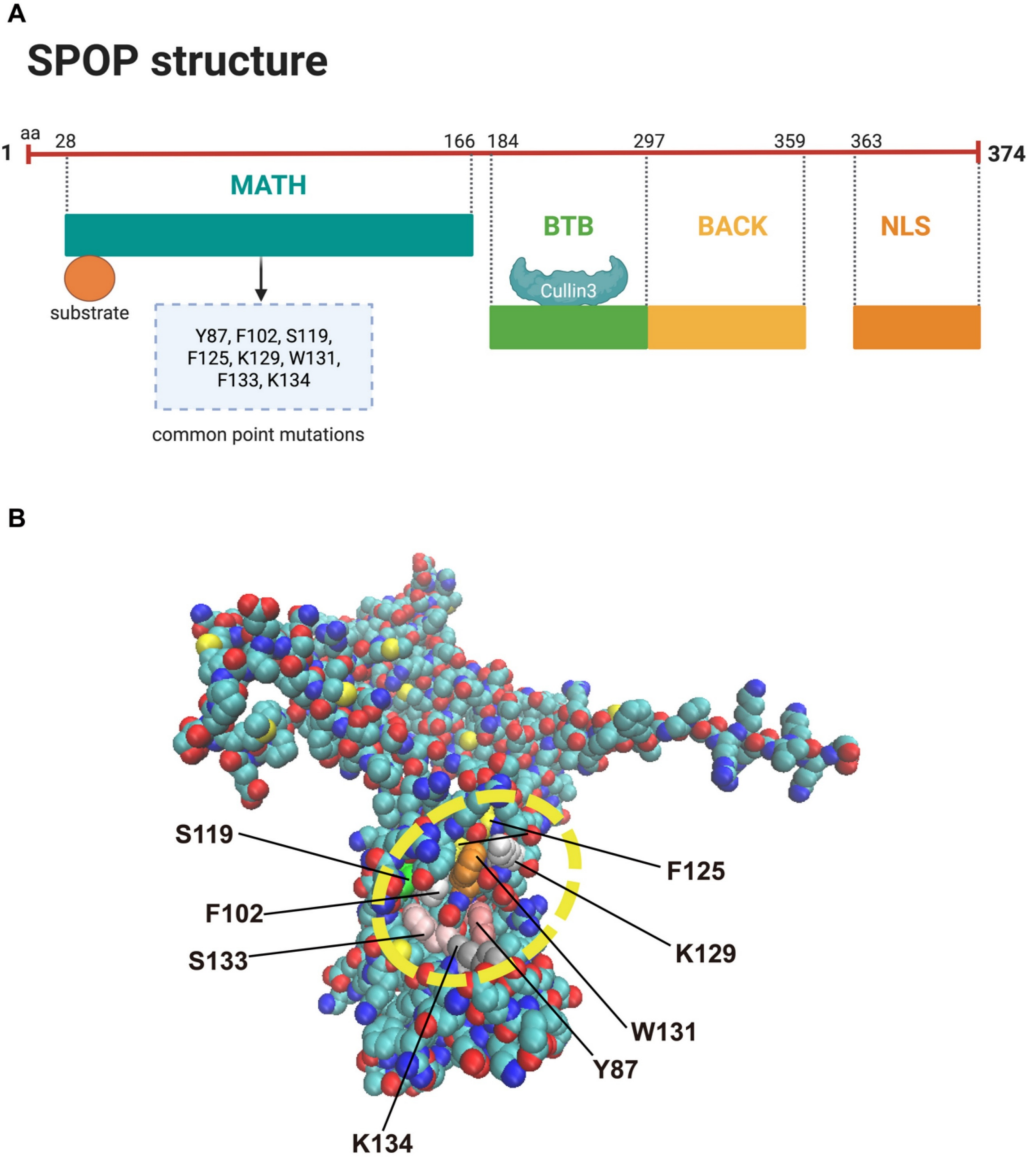
** Structural overview of SPOP.** (A) The SPOP protein consists of five key domains: the N-terminal MATH domain, which binds substrates containing the SBC motif (a serine/threonine-rich peptide motif, Φ-π-S-S/T-S/T, where Φ is nonpolar and π is polar); an internal BTB/POZ domain, which interacts with Cullin 3 and facilitates SPOP dimerization; a BACK domain, which mediates secondary dimerization; and a C-terminal NLS. (B) The structure of SPOP, along with its hotspot mutations in prostate cancer, is shown. BTB: Bric-à-brac/Tramtrack/Broad; MATH: Meprin and TRAF homology; NLS: nuclear localization sequence; SBC: SPOP-binding consensus.

SPOP was first identified by Nagai *et al.* in 1997 and is characterized by a typical POZ/BTB domain 29. Structurally, the SPOP protein consists of five domains: an N-terminal meprin and TRAF homology (MATH) domain that binds substrates containing the SPOP-binding consensus (SBC) motif (a serine/threonine-rich peptide motif, Φ-π-S-S/T-S/T, where Φ is nonpolar and π is polar); an internal BTB/POZ domain that interacts with Cullin 3 and facilitates SPOP dimerization; a BACK domain that mediates secondary dimerization; the 3-box, a subdomain within the BACK domain, enhances the SPOP-CUL3 interaction; and a C-terminal nuclear localization sequence (NLS) [Figure 3A] 28. The structure of SPOP and its hotspot mutations are depicted in Figure 3B. SPOP mutations are most commonly found in PCa, and Figure 3B highlights the most frequent mutation sites associated with this cancer 14. The clustering of SPOP alterations specifically within the MATH domain can be attributed to its functional and structural importance in substrate recognition and binding. The MATH domain is essential for SPOP's role as an E3 ubiquitin ligase, as it facilitates the recognition and binding of various substrates, including oncoproteins, for ubiquitination and degradation 24. Mutations in this domain can disrupt substrate interactions, impairing SPOP's ability to regulate processes like the cell cycle 30, apoptosis 31, and DNA repair 32. Structurally, the MATH domain is highly conserved and mediates multi-point binding to substrates through a distinct three-dimensional structure 33. Alterations in this region, through mutations or deletions, can destabilize the binding site or induce conformational changes that affect substrate specificity and SPOP's overall function 34. In cancers such as prostate, renal carcinoma, and endometrial cancer, SPOP mutations are often clustered in the MATH domain, leading to loss-of-function or gain-of-function alterations 31,35,36. Loss-of-function mutations impair substrate binding and prevent the degradation of oncogenic proteins, while gain-of-function mutations may create new binding interfaces that promote oncogenic pathways 35,37. The evolutionary conservation of the MATH domain suggests that mutations in this region are more likely to disrupt SPOP's core function, contributing to the high frequency of these mutations in cancer 34. Overall, the clustering of mutations in the MATH domain reflects its crucial role in substrate recognition, structural integrity, and tumor suppression, with alterations in this region significantly impacting cancer progression.

## 3. SPOP-regulated processes

As a key adaptor in CRL3-type E3 ligases, SPOP plays a critical role in tumorigenesis, supported by substantial physiological, pathological, and biochemical evidence 24. Key biochemical evidence indicates that SPOP facilitates the ubiquitination of its downstream substrates 24. The identification of diverse ubiquitin substrates has underscored the dual role of SPOP in tumorigenesis, thus posing challenges to cancer therapy and attracting significant attention 14. Thus, an accurate understanding of mechanisms for SPOP in cancer is critical for developing future effective drug development.

**Figure 4:**
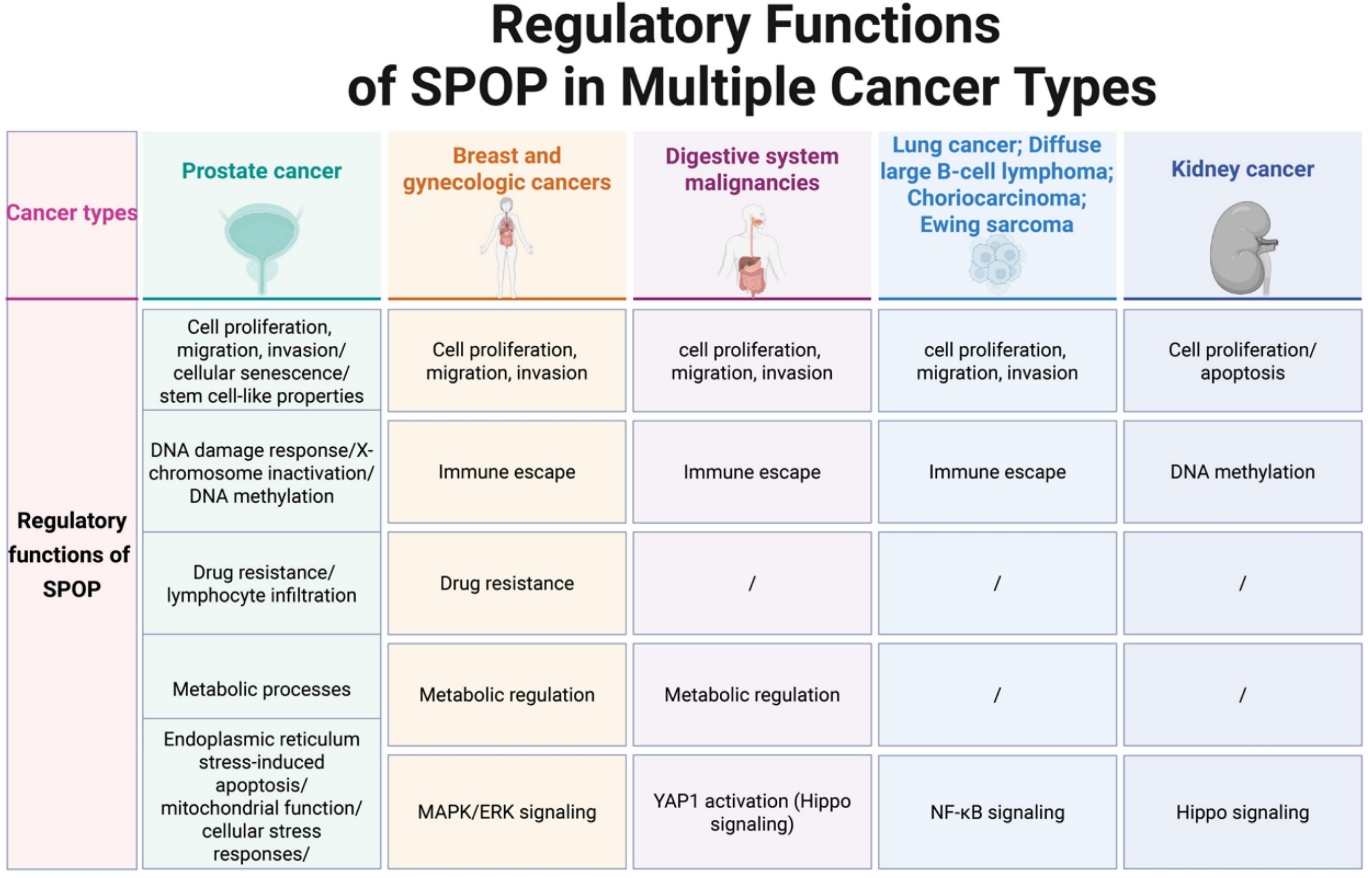
** Regulatory functions of SPOP across multiple cancer types.** This figure highlights the roles of SPOP in prostate cancer, breast and gynecologic cancers, digestive system malignancies, diffuse large B-cell lymphoma, choriocarcinoma, Ewing sarcoma, bladder cancer, and kidney cancer.

SPOP functions as a pivotal regulatory hub, orchestrating a broad spectrum of cellular processes critical to tumorigenesis across various cancer types [Figure 4]. In PCa, SPOP functions as a tumor suppressor, regulating cell proliferation/migration/invasion 14,30,38-47, drug resistance 35,48-51, DNA damage response (DDR) 52-55, X-chromosome inactivation 56, metabolic processes 57-59, cellular senescence 60, lymphocyte infiltration 61,62, stem cell-like properties 63,64, and endoplasmic reticulum stress-induced apoptosis 65. Of note, loss of SPOP further inhibits DNA hypermethylation while exacerbating mitochondrial dysfunction 66, AKT kinase activation 67, and aberrant cellular stress responses 68.

In breast and gynecologic cancers, multiple lines of evidence suggest that SPOP primarily functions as a tumor suppressor, influencing cell proliferation/migration/invasion 42,69,70, immune escape 71-73, MAPK/ERK signaling 74, and metabolic regulation 75. However, in breast cancer, SPOP appears to promote tumor metastasis by degrading BRMS1 76, a key metastasis suppressor gene. In endometrial cancer, SPOP-specific mutants, which markedly reduce BET protein levels, enhance cancer cell sensitivity to BET inhibitors 36. In cervical cancer, SPOP seems to promote paclitaxel resistance and diminish the efficacy of immune therapies, thereby contributing to tumor progression 72,77**;** however, these findings warrant further investigation.

In digestive system malignancies, SPOP primarily functions as a tumor suppressor, regulating cell proliferation/migration/invasion 78-83, YAP1 activation 84, metabolic processes 85, and immune escape 86. Notably, the HCC-derived mutant SPOP-M35L exhibits enhanced interaction with IRF2BP2, leading to its ubiquitination and degradation, thereby promoting HCC cell proliferation and migration 37. Similarly, in other cancers, including lung cancer, diffuse large B-cell lymphoma (DLBCL), choriocarcinoma, and Ewing sarcoma, SPOP also exerts tumor-suppressive functions. In lung cancer and DLBCL, SPOP regulates cell proliferation, migration, invasion, and NF-κB signaling 87-90. Moreover, SPOP controls proliferation, migration, and invasion in choriocarcinoma and Ewing sarcoma 91,92, while in bladder cancer, it inhibits immune escape 93.

In KC, SPOP promotes tumor progression by enhancing proliferation, inhibiting apoptosis 31,94, and regulating H3K36me3 levels and Hippo signaling 95,96.

Notably, SPOP has recently been implicated in liquid-liquid phase separation (LLPS), a biophysical process where cellular components form membrane-less, dynamic compartments that play key roles in cellular functions such as signal transduction, transcription, and stress responses 25. SPOP's ability to form higher-order oligomers, combined with its intrinsically disordered regions (IDRs), allows it to undergo phase separation, creating liquid-like droplets that concentrate substrates for efficient ubiquitination 97. This phase separation enhances the specificity and efficiency of SPOP's E3 ligase activity by organizing both the enzyme and its substrates into localized areas. However, in the context of tumorigenesis, disruptions in SPOP's phase-separating ability can lead to the stabilization of oncogenic proteins that should otherwise be degraded, promoting uncontrolled cell proliferation and cancer progression 97. Mutations in SPOP, which impair its phase separation, can dysregulate key processes such as cell cycle control, DNA damage response, and apoptosis 25,34. Moreover, alterations in SPOP's interactions with other phase-separating proteins or changes in its phosphorylation status can further complicate its function, influencing its ubiquitin ligase activity and substrate fate 25. The emerging role of SPOP in phase separation underscores the complex interplay between genetic mutations and biophysical properties in cancer.

## 4. The regulation of SPOP

The regulation of SPOP expression occurs at multiple levels, including DNA methylation, which affects transcription 80,98, miRNAs that modulate translation 99-101, and phosphorylation and self-ubiquitination, which influence posttranscriptional modifications 102-104. Together, these regulatory processes ultimately alter either the expression or the function of SPOP. Table 1 summarizes the regulators that promote increased SPOP expression, while Table 2 outlines those that reduce SPOP expression, and Table 3 highlights the factors that influence its function.

## 5. Roles of SPOP substrates in human cancers

Growing evidence has clarified the role of SPOP in carcinogenesis, with its expression levels and mutation status varying in a context-dependent manner across human cancers. SPOP functions predominantly as a tumor suppressor in prostate, lung, gastric, liver, colon, and endometrial cancers 14,24, but acts as an oncogene in clear cell renal cell carcinoma (ccRCC) 14,31. The identification of an increasing number of its substrates within specific cancer types further underscores its significance in cancer [Table 4].

### 5.1 Tumor-suppressive functions of SPOP in PCa

**Figure 5:**
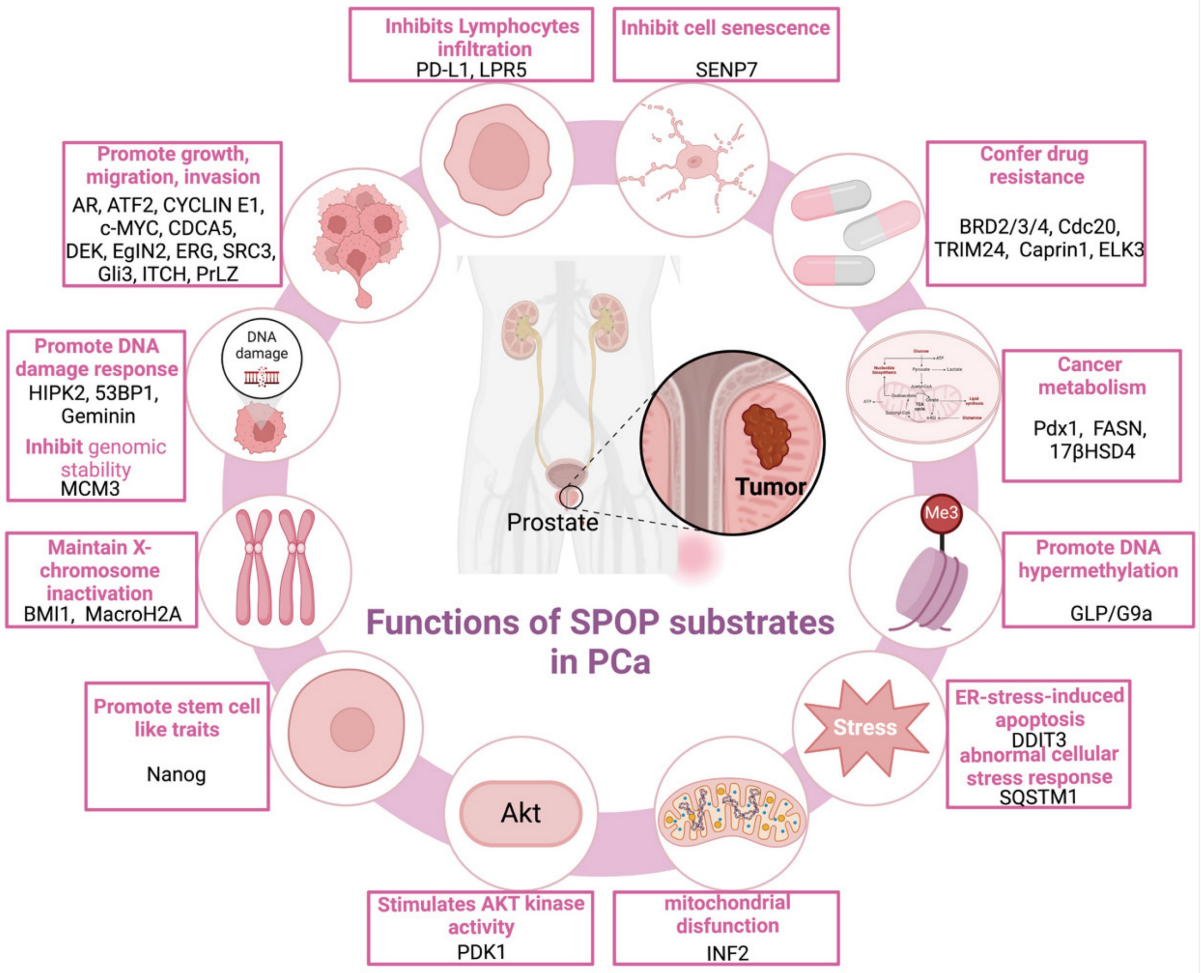
** Functions of SPOP Substrates in PCa**. This figure outlines the functional roles of SPOP substrates in PCa, highlighting how the ubiquitination-and either degradation or non-degradation-of specific substrates by SPOP impacts key cellular processes such as cell growth, apoptosis, androgen receptor signaling, and tumor progression. The diagram emphasizes how dysregulation of these processes, often resulting from SPOP mutations, contributes to the development and progression of PCa. PCa: prostate cancer; SPOP: Speckle-type POZ protein.

Physiological evidence from animal models and pathological evidence from human cancer specimens reveal frequent SPOP mutations, which are associated with a worse prognosis in PCa 57,118,119. These loss-of-function missense mutations predominantly cluster in the MATH domain [Figure 3B], the substrate-binding motif, potentially impairing or blocking substrate affinity 33. This failure to degrade oncogenic substrates can lead to the activation of oncogenic pathways. A diverse array of SPOP substrates has recently been identified in PCa, each playing a role in specific oncogenic pathways [Figure 5].

#### 5.1.1 Downstream substrates of SPOP associated with growth, migration and invasion

From Table 4 and Figure 5, it is evident that the core substrates promoting cell proliferation, migration, and invasion include AR, activating transcription factor 2 (ATF2), cyclin E1, c-MYC, cell division cycle associated protein 5 (CDCA5), DEK, Egl-9 family hypoxia inducible factor 2 (EglN2), ETS-related gene (ERG), steroid receptor coactivator 3 (SRC3), Gli3, ITCH, and prostate leucine zipper (PrLZ).

Androgens, primarily testosterone and dihydrotestosterone, play a crucial role in the differentiation and functioning of various components of the male reproductive system. The androgen receptor (AR) pathway serves as a key element in the signaling processes within healthy prostatic tissue 120. The AR signaling pathway is a well-recognized driver of PCa progression 120. Recent findings suggest that while wild-type (WT) SPOP can interact directly with the hinge region of AR at the SBC motif, its mutant forms lack this capacity. This interaction promotes AR ubiquitination and subsequent degradation, underscoring the regulatory role of SPOP in AR signaling 38,111. In 2014, An *et al*. demonstrated that SPOP could interact with the AR both *in vitro* and *in vivo*. However, only AR splice variants containing the SBC motif, such as the v567es variant, are capable of being bound by SPOP 111. Androgens diminish SPOP-mediated degradation of endogenous AR; however, this effect is significantly inhibited by the antiandrogen enzalutamide 111. Based on these findings, combining SPOP activators with antiandrogens could serve as a promising approach for therapeutic development. Additionally, Geng *et al.* revealed that WT SPOP, but not its mutant forms-such as SPOP-F102C, SPOP-F133V, SPOP-F125V, SPOP-S119N, SPOP-Y87C, and SPOP-Y87N-binds to AR, promoting its ubiquitination and subsequent degradation 38. Similarly, the presence of SPOP mutations can lead to a partial decrease in sensitivity to enzalutamide 38. However, they proposed that ARv7 indirectly interacts with WT-SPOP through the formation of AR-full-length (FL) /ARv7 heterodimers in 22Rv1 prostate adenocarcinoma cells, even in the absence of the SBC 38. In immunocompromised mice, they observed that SPOP-F102C xenografts grew significantly faster and exhibited elevated AR protein levels compared with WT-SPOP xenografts 38. In patient cohorts, a strong correlation was observed between the SPOP signature score and AR activity score 38. Therefore, enhancing the interaction between ARv7 and WT SPOP could be a promising therapeutic strategy for PCa treatment.

The ATF/CREB bZIP family includes the transcription factor ATF2, a ubiquitously expressed protein 121. ATF2, while predominantly found in brain tissue, is a protein expressed throughout various tissues and plays a significant role in regulating transcription, remodeling chromatin, and responding to DNA damage 121. The total loss of ATF2 in somatic cells leads to lethality after birth, whereas a partial dysregulation of ATF2 has been associated with cancer development 121. In 2014, Ricote *et al.* reported that PCa patients exhibit overexpression of phosphorylated ATF2, as demonstrated through immunohistochemical and western blot analyses. This overexpression is associated with enhanced cell proliferation and survival 122. Subsequent studies have identified several SBC motifs in ATF2, which are essential for its degradation via SPOP-mediated ubiquitination. Notably, PCa-associated SPOP mutants impair this process, leading to defective ATF2 degradation and consequently promoting cell proliferation, invasion, and migration 39.

Cyclin E1, which acts as an activator for cyclin-dependent kinase 2 (CDK2), is predominantly expressed during the transition from G1 to S phase of the cell cycle 14. This protein plays a crucial role in facilitating DNA replication, centrosome duplication, and histone biosynthesis, all of which are integral to the commencement of the S phase 14. It is an oncogene and key regulator of S phase progression in the cell cycle, is implicated in PCa proliferation. Zhang *et al.* demonstrated that cyclin E1 plays a crucial role in PCa cell proliferation 40. In PCa tissues, the relative expression of cyclin E1 mRNA was significantly correlated with the progression of high-grade carcinomas, particularly those with a Gleason score greater than 7 123. The SPOP/CUL3/RBX1 complex mediates polyubiquitination and subsequent degradation of cyclin E1, thereby inhibiting PCa cell proliferation and migration 40. Conversely, proteins such as OTUB1 promote PCa progression by deubiquitinating and stabilizing cyclin E1 124. These findings suggest that cyclin E1 functions as a tumor promoter in PCa and is a substrate of SPOP. Dysregulated ubiquitination-mediated proteolysis of cyclin E1 contributes to PCa development.

Previous studies have shown that elevated levels of c-MYC expression are linked to aggressive forms of human PCa 125. Recent work has uncovered that AR signaling regulates c-MYC expression, which has important implications for the effectiveness of AR signaling antagonists 126,127. This newly identified regulatory axis sheds light on the complex mechanisms driving PCa progression and therapeutic responses. Geng and colleagues demonstrated that WT-SPOP directly interacts with c-MYC, promoting its ubiquitination and subsequent proteasomal degradation in PCa cells. This regulatory process, however, is disrupted in SPOP mutants with altered substrate binding pockets 41. Furthermore, SPOP plays a pivotal role in regulating prostate epithelial cell proliferation, indicating its broader involvement in prostate homeostasis and carcinogenesis 41. Mice with prostate-specific heterozygous or homozygous SPOP deletion (*SPOP^-/+^* or *SPOP^-/-^*) displayed increased prostate mass and elevated c-MYC protein expression, ultimately developing prostatic intraepithelial neoplasia (PIN) 41. Clinical data from human PCa samples further revealed a strong association between high c-MYC transcriptional activity and poor clinical outcomes 41. Taken together, these findings, along with mechanistic studies, suggest that c-MYC is a bona fide SPOP substrate. Thus, SPOP appears to exert its tumor-suppressive function, in part, by targeting c-MYC for ubiquitination-mediated proteasomal degradation.

CDCA5, commonly referred to as sororin, was first recognized as a substrate of the anaphase-promoting complex 128. This protein plays a crucial role in maintaining the binding of cohesin to chromatids throughout the S and G2/M phases of the cell cycle, and it is also involved in the repair of DNA double-strand breaks 128. Recent studies have shown that CDCA5 mRNA and protein levels are significantly upregulated in PCa tissues, with high expression correlating with poor prognosis. These findings highlight CDCA5 as a potential biomarker and therapeutic target. Functional studies further confirm its oncogenic role in PCa, as CDCA5 knockdown inhibits cell proliferation in C4-2 and PC-3 cell lines both *in vitro* and *in vivo* 129. These findings provide compelling evidence for the critical role of CDCA5 in sustaining PCa growth and progression and underscore its potential as a therapeutic target. A pivotal study revealed that WT-SPOP, but not its mutant form, directly interacts with CDCA5 and promotes its polyubiquitination-mediated degradation in DU145 PCa cells 30. In addition, SPOP influences the growth of both DU145 and PC-3 PCa cell lines through, or at least partially through, its regulation of CDCA5 30. The AR-negative (AR-) PCa cell lines DU145 and PC-3 have been extensively studied in this context. However, the potential occurrence of SPOP-mediated CDCA5 degradation in AR-positive (AR+) cells remains to be elucidated.

Elevated DEK expression has been observed in both neuroendocrine prostate cancer (NEPC) xenograft models and clinical specimens 130. Evidence shows that DEK is a substrate of SPOP-mediated ubiquitination, with SPOP mutations impairing DEK degradation and contributing to cellular dysregulation 112. In PCa, overexpression of WT-DEK or SPOP-binding-deficient DEK mutants enhances cellular invasiveness 112. The SPOP Y87N mutant disrupts DEK degradation, promoting DEK accumulation and enhancing sphere-forming capacity in prostate epithelial cells, suggesting a role in tumor initiation 112. Targeted DEK depletion in SPOP-Y87N cells reduces sphere-forming ability 112, highlighting DEK's critical role in SPOP-mutant PCa and suggesting a potential therapeutic target. SPOP regulation of DEK may influence stem-like phenotypes in PCa 112. This regulatory axis potentially contributes to cellular plasticity and the acquisition of cancer stem cell-like properties, which are increasingly recognized as key factors in tumor progression and therapeutic resistance.

The EglN family of prolyl hydroxylases (EglN1, EglN2, EglN3) regulates the stability of hypoxia-inducible factor alpha (HIFα) subunits, but EglN2 also has HIF-independent roles in cellular proliferation 131,132. Recent investigations into the role of EglN2 in PCa have revealed intriguing patterns of expression and clinical correlation, further expanding our understanding of this prolyl hydroxylase's significance in various cancer types. Notably, studies have shown that EglN2 is aberrantly expressed in PCa tissues, with its expression levels correlating with Gleason score 43. EglN2 knockdown significantly inhibits PC3 cell growth *in vitro* and in a xenograft model, highlighting its role in PCa progression 43. In AR+ PCa cell lines (RV1, LNCaP, C4-2), silencing AR downregulates EglN2 transcription. In contrast, ectopic AR expression in the AR- PC-3 cell line upregulates EglN2 at both mRNA and protein levels 43. SPOP interacts with and promotes the degradation of EglN2. However, SPOP mutants associated with PCa patients show impaired ability to degrade EglN2, resulting in elevated EglN2 levels, which contribute to PCa progression 43. These findings implicate EglN2 as having pro-oncogenic functions in PCa, while suggesting that SPOP exerts tumor-suppressive effects, at least partially through its role in promoting EglN2 degradation.

PCa is often characterized by *TMPRSS2* gene fusions with ETS family transcription factors, particularly the *TMPRSS2-ERG* fusion, which occurs in about 50% of cases and drives disease progression through aberrant ETS expression 133. Notably, *TMPRSS2-ERG* is considered an early molecular event, as it has been detected in the PCa precursor lesion high-grade prostatic intraepithelial neoplasia (HGPIN), suggestive of its association with invasiveness and disease initiation 134. Two independent studies have shown that the E3 ubiquitin ligase adaptor SPOP regulates ERG ubiquitination and subsequent proteasomal degradation 44,135. However, N-terminal-truncated ERG proteins encoded by *TMPRSS2-ERG* fusions evade this process by impairing the degron, a critical region for SPOP-mediated ubiquitination 135. In C4-2 cells, SPOP mutants fail to bind and degrade ERG, highlighting the importance of functional SPOP in regulating ERG levels 135. Several studies have reported near-complete mutual exclusivity between *SPOP* mutations and *ERG* rearrangements, suggesting distinct molecular subclasses of PCa 136,137. Consistent with these findings, Shoag *et al.* demonstrated that SPOP-mutant PCa lacks detectable ERG protein expression in human samples 138. Furthermore, gene expression comparisons between *SPOP*-mutant and *ERG*-fusion organoid models revealed distinct transcriptional signatures, reinforcing the divergent molecular pathways underlying these PCa subtypes 138. Thus, further investigation is needed to determine whether ERG acts as an effector of SPOP mutation in human PCa.

The p160 SRC family, comprising SRC1, SRC2, and SRC3, plays crucial roles in cancer initiation, progression, and metastasis through multiple pathways 139,140. In PCa, SRC overexpression correlates with high tumor recurrence, advanced disease stage, and elevated tumor grade 140. SRC3, an AR-preferential coactivator, is particularly important for PCa proliferation and survival 141,142. Geng *et al.* demonstrated that WT-SPOP promotes SRC3 degradation, thereby suppressing AR transcriptional activity, while sparing SRC1 and SRC2 143. Notably, all PCa-associated SPOP mutants fail to bind SRC3, highlighting the critical role of SPOP in regulating SRC3 and AR signaling 143. Therefore, SRC3 and AR are key downstream effectors of SPOP, critically influencing PCa pathophysiology and therapy resistance.

The Hh signaling pathway, frequently hyperactive in various human malignancies, including PCa, plays a crucial role in driving cancer metastasis 144-147. The GLI zinc-finger transcription factors are the ultimate effectors of the Hh pathway, with GLI1 and GLI2 acting as positive regulators, and GLI3 generally functioning as a negative regulator 146. Paradoxically, GLI3 upregulation is observed in many prostate tumors, with its expression levels surpassing those of GLI1 and GLI2 in various PCa models 45,148. GLI3 is a substrate of SPOP, which targets it for proteasomal degradation 149. However, oncogenic SPOP mutations stabilize GLI3 and activate an AR/GLI3 axis, potentially driving PCa development and castration resistance 45. Depletion of GLI3 inhibits castration-resistant PCa formation by disrupting AR/GLI3 crosstalk 45, suggesting that GLI3-specific inhibitors may offer a rational therapeutic strategy for PCa.

ITCH, a HECT E3 ubiquitin ligase, plays diverse roles in cellular processes and exhibits both anti- and pro-tumorigenic functions in a cancer type-specific manner 150. In PCa, evidence suggests that SPOP mediates ITCH ubiquitination and degradation, thereby protecting against cancer metastasis 46. This finding implies that ITCH is a substrate of SPOP, warranting further investigation to elucidate the precise mechanisms and consequences of this regulatory axis in PCa progression.

PrLZ, a member of the tumor protein D52 (TPD52) family, is a prostate-specific protein implicated in multiple oncogenic processes 151. Overexpression of PrLZ promotes PCa progression by upregulating AR expression, enhancing cell growth, and conferring resistance to docetaxel chemotherapy 152-156. Recent studies have shown that PrLZ is a substrate of SPOP, with SPOP mediating its degradation 47. Although PrLZ lacks a classic SBC motif, it contains a SBC-like motif, and mutation of Ser40 in this motif nearly abolishes SPOP-mediated degradation 47. While the pathological Ser40 mutation has not been identified in patient databases, these findings suggest that clinical SPOP mutations could lead to aberrant PrLZ accumulation, driving tumor progression and contributing to poor outcomes in PCa patients. These studies underscore the importance of SPOP-mediated regulation of PrLZ in PCa development and progression, highlighting the need for further research to elucidate the full implications of this interaction. Additionally, these findings may inform potential therapeutic strategies targeting the SPOP-PrLZ axis in PCa treatment.

#### 5.1.2 Downstream substrates of SPOP associated with drug resistance

From Table 4 and Figure 5, we can see that downstream substrates of SPOP implicated in drug resistance include bromodomain containing proteins 2/3/4 (BRD2/3/4), cell division cycle 20 (Cdc20), tripartite motif containing 24 (TRIM24), Caprin1, and ELK3.

Bromodomain and extraterminal domain (BET) proteins, including BRD2, BRD3, and BRD4, co-regulate transcriptional activation and repression 157. While BRD2 and BRD4 are essential for cell growth, the role of BRD3 in this process remains unclear 157. Recent evidence shows that SPOP targets BRD2, BRD3, and BRD4 for ubiquitination-mediated degradation 35. Oncogenic SPOP mutations impair this degradation, leading to BET protein accumulation and conferring resistance to BET inhibitors in PCa cells 35. Consistently, sequencing data reveal that SPOP-mutated tumors exhibit strong or intermediate staining of BET proteins 35. Collectively, these findings suggest that SPOP may function as a tumor suppressor in PCa, in part by promoting the degradation of BRD2, BRD3, and BRD4.

Cdc20, a subunit of the anaphase-promoting complex/cyclosome (APC/C) ubiquitin ligase, plays a crucial role in regulating the M and G1 phases of the cell cycle by mediating the ubiquitination and degradation of securin and cyclin B, thereby promoting anaphase onset and mitotic exit 158. Recent studies have uncovered the oncogenic properties of Cdc20, with its overexpression observed in numerous human cancers 158-161, including non-small cell lung cancer (NSCLC) 162, breast cancer 163,164, pancreatic cancer 165, CRC 166, HCC 167, gastric cancer (GC) 168, glioblastoma 169, PCa 170, and bladder, oral, and cervical cancers 171,172. Genetic ablation of *CDC20* leads to efficient tumor regression both *in vitro* and *in vivo* 170,173, making it an attractive target for cancer therapy 158. Wu *et al.* identified Cdc20 as a novel ubiquitin substrate of the E3 ubiquitin ligase adaptor SPOP, which promotes Cdc20 polyubiquitination and subsequent degradation 48. Consequently, PCa cells deficient in SPOP and exhibiting increased Cdc20 expression demonstrated resistance to pharmacological inhibition of Cdc20 48. This finding provides a rationale for designing therapeutic strategies using Cdc20 inhibitors to treat SPOP-WT PCa, where SPOP's tumor-suppressive function remains intact.

TRIM24, also known as TIFα, is a member of the TRIM family and primarily functions as a dual epigenetic reader 174,175. TRIM24 enhances AR signaling and promotes proliferation, and it has been identified as an effector substrate of SPOP 51. Oncogenic SPOP mutants impair the ubiquitylation and proteasomal degradation of TRIM24, leading to its stabilization 51. This stabilization amplifies AR signaling, resulting in significant upregulation of co-activated AR and TRIM24 target genes in castration-resistant prostate cancer (CRPC) 51. Additionally, TRIM24 protein expression increases as PCa progresses from primary PC to CRPC 51. In LNCaP cells expressing the SPOP Y87C mutant, there is a significant growth advantage over SPOP-WT cells, particularly under low androgen conditions 51. This growth advantage is abrogated when TRIM24 expression is knocked down by specific short hairpin RNA (shRNA), indicating that the stabilization of TRIM24 via SPOP mutations is essential for promoting PCa cell proliferation under low androgen conditions 51.

Caprin1 plays a crucial role in nucleating stress granule (SG) assembly in response to environmental stress 176. Caprin1 is found to be upregulated in various types of cancers 177,178. In PCa, SPOP mutation status is linked to increased Caprin1 expression 49. Cytoplasmic, but not nuclear, SPOP promotes the ubiquitination and degradation of Caprin1 49. SPOP specifically regulates Caprin1-dependent SG assembly in C4-2 cells, and PCa-associated SPOP mutations enhance cancer cell survival by elevating Caprin1 levels 49. Knockout of SPOP or expression of PCa-associated SPOP mutants confers resistance to cell death triggered by SG inducers, including docetaxel, sodium arsenite, and H₂O₂, in PCa cells 49. These findings underscore the importance of SPOP-mediated regulation of Caprin1 in PCa and suggest that targeting this interaction may have therapeutic implications.

ELK3, also known as Net, SAP-2, or Erp, is a member of the ETS family of transcription factors. It forms a ternary complex with serum response factor (SRF) to regulate key target genes, such as* C-FOS*, involved in fundamental cellular processes like proliferation, differentiation, and stress responses 179. Studies have shown that silencing *ELK3* in PCa cells induces S-M phase arrest and apoptosis, while also upregulating SERPINE1 expression, which subsequently inhibits cell migration 180. Recent research reveals that SPOP interacts with ELK3 to promote its ubiquitination and degradation, a process driven by checkpoint kinase-mediated phosphorylation 50. This regulation of ELK3 stability by SPOP impacts c-fos-driven proliferation and invasion in PCa cells 50. Docetaxel treatment induces cell death by activating checkpoint kinase- and SPOP-mediated ELK3 degradation; however, PCa cells with SPOP depletion or mutation exhibit resistance to this mechanism 50. These findings suggest that targeting ELK3 activation and its stability-enhancing pathways may offer effective therapeutic strategies to overcome docetaxel resistance in PCa, potentially improving the treatment of CRPC, warranting further investigation.

#### 5.1.3 Downstream substrates of SPOP associated with DNA damage response

According to Table 4 and Figure 5, SPOP downstream substrates involved in the DDR include homeodomain interacting protein kinase 2 (HIPK2), p53 binding protein 1 (53BP1), GEMININ, and minichromosome maintenance complex component 3 (MCM3).

HIPK2, a member of the HIPK family, is a well-characterized serine/threonine protein kinase involved in various biological processes, including the DDR 181,182. It has been identified as a tumor suppressor, activated by the checkpoint kinase ataxia-telangiectasia mutated (ATM), and triggers apoptosis through the regulatory phosphorylation of the tumor suppressor p53 182,183. Several reports suggest that HIPK2 plays a dual role in determining cell fate following DNA damage 184-187. After sublethal DNA damage, HIPK2 phosphorylates the epigenetic regulator heterochromatin protein 1γ (HP1γ), stimulating the DDR. In contrast, under severe damage, HIPK2 phosphorylates p53 at Ser46, irreversibly driving cells toward apoptosis 184-187. Recent studies have identified HIPK2 as a novel SPOP-interacting protein 52. In PC-3/DU145 cells, SPOP promotes non-degradative ubiquitination of HIPK2 52. This interaction is facilitated by ATM-mediated phosphorylation of SPOP at Ser119 upon DNA damage, which enhances SPOP binding to HIPK2 52. The binding of SPOP to HIPK2 increases HIPK2's phosphorylation activity toward HP1γ, promoting the dissociation of HP1γ from the trimethylation of histone H3 at lysine 9 (H3K9me3), thereby initiating the DDR 52. Thus, the SPOP-HIPK2 axis plays a crucial role in facilitating the DDR.

53BP1 regulates nonhomologous end joining (NHEJ) and homologous recombination (HR) repair pathways 188. It promotes NHEJ and inhibits HR by preventing DNA end resection, which can lead to genomic instability 189,190. Additionally, SPOP induces non-degradable polyubiquitination of 53BP1, facilitating its extraction from chromatin and promoting HR repair over NHEJ during DNA replication 53. However, cancer-derived SPOP mutations disrupt the SPOP-53BP1 interaction, leading to HR defects and chromosomal instability 53. As a result, tumors with SPOP mutations may benefit from Poly(ADP-ribose) polymerase (PARP) inhibition, a DNA repair-targeted therapy. This notion was recently confirmed by research from Xiaofeng Jin and colleagues 32.

Geminin plays a critical role in the cell cycle, with two key functions: inhibiting DNA replication initiation and undergoing degradation during the metaphase-anaphase transition 191. It has been implicated in regulating differentiation, cell proliferation, and the DDR 192,193. Ma *et al.* suggested that SPOP promotes non-degradable polyubiquitination of geminin at lysine residues 100 and 127, preventing DNA replication over-firing and genome instability 55. However, mutations in SPOP lead to geminin inactivation, resulting in undesired replication over-firing, replication catastrophe, and extensive DNA breaks 55.

MCM3 is a member of the MCM protein family, essential for DNA synthesis and the regulation of DNA replication initiation and elongation 194,195. Aberrant expression and activation of MCMs are frequently observed in various malignancies, contributing to genome instability 196. In 2021, researchers demonstrated that SPOP ubiquitinates and degrades MCM3 in response to DNA damage 54. This process is inhibited by phosphorylation of SPOP at Ser119 54. The underlying mechanism involves ATM-mediated phosphorylation of SPOP, which is required for the dissociation of the SPOP-MCM3 complex and subsequent degradation of MCM3 54.

In summary, SPOP regulates four critical substrates—HIPK2, 53BP1, MCM3, and geminin—that collectively contribute to genome stability. Notably, while most of these substrates undergo non-degradable polyubiquitination, MCM3 is a unique exception. Importantly, MCM3 alone inhibits the DDR, whereas the other substrates actively promote it, highlighting SPOP's essential role in supporting DDR pathways.

#### 5.1.4 Downstream substrates of SPOP associated with X-chromosome inactivation

As detailed in Table 4 and Figure 5, SPOP regulates several downstream substrates involved in X-chromosome inactivation, including B-lymphoma Mo-MLV insertion region 1 (BMI1) and macroH2A2.

BMI1 is a component of the maintenance polycomb repressive complex 1 (PRC1), which is part of the epigenetic gene regulators known as polycomb group (PcG) proteins 56. SPOP, in conjunction with CULLIN3, mediates the non-degradative ubiquitination of BMI1, thereby stabilizing X chromosome inactivation 56.

Histone variants, such as macroH2A2 (previously referred to as H2AFY2), differ from core histones due to key amino acid variations. Specifically, macroH2A2 is a closely related variant of the core histone H2A, sharing only about 60% sequence identity in its histone domain 197. Similar to BMI1, SPOP ubiquitinates macroH2A2, impairing its localization to the inactive X chromosome without affecting its overall stability 56.

#### 5.1.5 Downstream substrates of SPOP associated with cancer metabolism

Based on Table 4 and Figure 5, SPOP modulates key downstream substrates implicated in cancer metabolism, such as pancreatic duodenal homeobox 1 (Pdx1), FASN, and 17βHSD4.

Pdx1 is a transcription factor essential for pancreatic development during embryogenesis and the survival of pancreatic cells in adults 198,199. Recent studies have demonstrated that SPOP targets Pdx1 for ubiquitination and proteasomal degradation, a regulation associated with improved β-cell function and mass, thereby enhancing glucose homeostasis and β-cell survival 57. However, no established link between Pdx1 and PCa exists, warranting further investigation.

FASN, the rate-limiting enzyme in de novo lipogenesis, is often upregulated in cancer, providing growth and survival advantages across various malignancies, including PCa 200-203. In 2019, Gang* et al.* reported that FASN is a substrate of SPOP, and their interaction facilitates FASN ubiquitination and proteasome-dependent degradation 58. As a result, FASN serves as one of the key mediators of SPOP-induced inhibition of PCa cell growth 58. Given that SPOP fails to regulate FASN in SPOP-mutant PCa, targeting FASN or its downstream metabolic pathways represents a promising therapeutic strategy.

17βHSD4, encoded by *HSD17B4*, traditionally inactivates testosterone and dihydrotestosterone by converting them to their inert 17-keto forms 204. Among its five alternative splice forms, only isoform 2 encodes an enzyme capable of inactivating these hormones. The regulation of HSD17B2, HSD17B4, and HSD17B5 by ligands of LXR, VDR, and AR in PCa cells is complex, yet functional expression of isoform 2 is specifically suppressed during CRPC development 204,205. SPOP interacts with a functional SBC motif in 17βHSD4, facilitating its non-degradable K27- and K29-linked polyubiquitination 59. This action is counteracted by serum- and glucocorticoid-regulated kinase-3 (SGK3)-mediated phosphorylation of serine 318 (S318) within the SBC motif 59. Phosphorylation at S318 enhances the binding of the SKP2 E3 ligase, which then induces K48-linked polyubiquitination and proteasomal degradation of 17βHSD4 59. Consequently, mutations in SPOP or overexpression of SKP2 promote PCa progression by reducing 17βHSD4 levels and enhancing intertumoral androgen production.

#### 5.1.6 Downstream substrates of SPOP associated with cell senescence

As evidenced by Table 4 and Figure 5, SPOP regulates key downstream substrates involved in cell senescence, including Sentrin/SUMO-specific protease 7 (SENP7).

SENP7, a SUMO2/3-specific protease, plays a crucial role in various physiological and pathological processes, including epithelial-mesenchymal transition (EMT), cancer cell motility and invasiveness, DNA repair, and innate immune responses 206-209. Recent studies have shown that SPOP targets SENP7 for degradation during senescence, while cancer-associated SPOP mutants are impaired in this function 60. Mechanistically, SPOP-mediated SENP7 downregulation increases the sumoylation levels of HP1α, leading to gene silencing and promoting cellular senescence, an important tumor suppression mechanism 60. These findings underscore SPOP's role as a tumor suppressor and provide a rationale for designing novel therapeutic strategies targeting the SPOP-SENP7-HP1α axis.

#### 5.1.7 Downstream substrates of SPOP associated with lymphocytes infiltration

As evidenced by the tabulated results (Table 4) and corresponding visualization (Figure 5), SPOP regulates key downstream substrates involved in lymphocyte infiltration, including programmed death-ligand 1 (PD-L1) and low-density lipoprotein receptor-related protein 5 (LRP5).

PD-L1, primarily expressed by tumor cells, interacts with its receptor, programmed death receptor-1 (PD-1), playing a pivotal role in immune tolerance or escape 210,211. Recent research has demonstrated that cyclin D-CDK4 and SPOP regulate PD-L1 protein levels via proteasome-mediated degradation 61. Cyclin D-CDK4 mediates SPOP phosphorylation, leading to its degradation by APC/Cdh1, thereby elevating PD-L1 levels 61. Additionally, loss-of-function SPOP mutations result in increased PD-L1 levels and reduced tumor-infiltrating lymphocytes (TILs) in both mouse tumors and primary human PCa specimens 61. These findings suggest that combining SPOP activators or CDK4/6 inhibitors with immune checkpoint inhibitors targeting PD-L1 may enhance therapeutic efficacy in human cancers.

Blood lipids and apolipoproteins assemble into lipoproteins, which are distributed throughout the body via the circulatory system. Tissues internalize these lipoproteins through LRP on the cell surface to support normal cellular functions. In PCa patients, lipid profiles are significantly altered, and genetic variations in *APOE* and *APOJ* have been implicated in disease development and progression 212. As previously mentioned, SPOP regulates lipid metabolism by decreasing the expression of FASN and fatty acid synthesis, contributing to tumor suppression 58. Similarly, the intracellular tail of LRP5 contains a SPOP binding site, facilitating direct interaction between LRP5 and SPOP 62. However, the functions of the SPOP-FASN axis and the SPOP-LRP5 axis differ. Specifically, overexpression of the LRP5 tail shifts the regulatory balance toward enhanced Daxx-mediated transcriptional inhibition, subsequently diminishing T cell activity in co-culture systems 62. Interestingly, the SPOP-F133V and SPOP-A227V mutations uniquely elevate PD-1 and PD-L1 protein levels 62. Consistently, these SPOP variants exert pronounced inhibitory effects on T cells relative to WT SPOP in co-culture 62. This SPOP-LRP5 axis is crucial, as specific *SPOP* genetic variants differentially influence immune checkpoint expression and activity within the PCa microenvironment.

#### 5.1.8 Downstream substrates of SPOP associated with stem cell-like traits

According to Table 4 and Figure 5, the downstream substrate of SPOP associated with stem cell-like traits is Nanog. Nanog, a master transcriptional regulator of stemness in cancer stem cells (CSCs), is frequently aberrantly expressed in various cancer types 213. In 2019, two reports indicated that SPOP promotes Nanog poly-ubiquitination and subsequent degradation via a conserved SBC motif, thereby regulating PCa cell stem traits 63,64. Pin1 and the AMPK-BRAF signaling axis were identified as upstream negative regulators of SPOP, blocking the interaction between SPOP and Nanog. Specifically, BRAF phosphorylates Nanog at Ser68 63,64. Notably, PCa-associated mutations in SPOP or the S68Y mutation in Nanog disrupt SPOP-mediated degradation of Nanog, leading to elevated cancer stem cell traits and PCa progression 63,64. Therefore, targeting the Pin1-SPOP-Nanog axis and the AMPK-BRAF-Nanog/SPOP-Nanog axis may offer promising therapeutic strategies for PCa in the future.

#### 5.1.9 Downstream substrates of SPOP associated with ER-stress-induced apoptosis

DNA damage inducible transcript 3 (DDIT3), also known as GADD153 or CHOP, is an endoplasm transcription factor that plays crucial roles in various stress responses and regulates cancer stemness across diverse tumor types 214,215. For instance, DDIT3 is associated with prognosis and the immune microenvironment in breast cancer and contributes to the progression of PCa 216-218. SPOP recruits DDIT3 for its ubiquitination and subsequent degradation. SPOP recognizes an SBC motif in the transactivation domain of DDIT3, triggering its degradation via the ubiquitin-proteasome pathway 65. Notably, PCa-associated mutants of SPOP are defective in this function 65. Therefore, in PCa, the DDIT3-SPOP axis significantly influences tumor growth and progression. Disruptions in this axis can lead to abnormal protein turnover, resulting in the accumulation of oncogenic proteins that fuel tumor development. Moreover, mutations in SPOP, frequently found in PCa, may compromise the function of the DDIT3-SPOP axis, contributing to therapy resistance and more aggressive cancer phenotypes. Consequently, targeting the DDIT3-SPOP axis offers a promising therapeutic strategy for PCa.

#### 5.1.10 Downstream substrates of SPOP associated with mitochondrial disfunction

The quantitative findings summarized in Table 4, along with the categorical organization in Figure 5, inverted formin 2 (INF2) is a downstream substrate of SPOP linked to mitochondrial dysfunction. INF2, a distinctive vertebrate formin protein, enhances both actin polymerization and depolymerization 219. SPOP binds to the SBC motif in the C-terminal region of INF2, triggering atypical polyubiquitination. This modification does not destabilize INF2 but decreases its localization to the ER and the formation of DRP1 puncta on mitochondria, impairing its role in promoting mitochondrial fission 113. However, both INF2 mutants and PCa-associated SPOP mutants promote mitochondrial fission 113. Additionally, deletion of the NLS sequence causes PCa-associated SPOP mutants to localize in the cytosol as puncta. Unlike WT SPOP, these mutants do not affect the endoplasmic reticulum localization of INF2 113. Therefore, SPOP may perform its tumor-suppressive functions in both the nucleus and the cytoplasm.

#### 5.1.11 Downstream substrates of SPOP associated with DNA hypermethylation

As depicted in Table 4 and Figure 5, GLP and G9a are downstream substrates of SPOP linked to DNA hypermethylation. GLP, encoded by *EHMT1*, and G9a, encoded by *EHMT2*, form a protein complex that functions as a euchromatic histone methyltransferase (HMTase), catalyzing the mono- and di-methylation of H3K9me1/2, which leads to the epigenetic silencing of target genes 220,221. SPOP interacts with GLP, promoting its polyubiquitination and subsequent degradation. Mutations in SPOP result in the stabilization of GLP and G9a, causing abnormal upregulation of global DNA hypermethylation in a subset of tumor suppressor genes, including* FOXO3*,* GATA5*, and *NDRG1* 66. The DNA methylation inhibitor 5-azacytidine effectively reactivates the expression of these tumor suppressor genes, inhibits the growth of SPOP-mutated PCa cells both *in vitro* and *in vivo*, and enhances the anti-cancer efficacy of docetaxel 66. Therefore, for SPOP-mutated PCa, the use of methylation inhibitors, either alone or in combination with docetaxel, should be considered.

#### 5.1.12 Downstream substrates of SPOP associated with AKT kinase activity

According to Table 4 and Figure 5, 3-phosphoinositide-dependent kinase 1 (PDK1) is a downstream substrate of SPOP associated with AKT kinase activity. It was initially isolated from tissue extracts as an enzyme that phosphorylates the T-loop of PKB at Thr308 in the presence of PtdIns (3,4,5) P3 (PIP3) 222,223. SPOP directly binds to PDK1 through a consensus degron in a phosphorylation-dependent manner, regulated by CK1 and GSK3β 67. Pathologically, mutations in SPOP associated with PCa disrupt PDK1 degradation, while mutations within or near the PDK1 degron—either by blocking SPOP binding or inhibiting CK1/GSK3β-mediated PDK1 phosphorylation—enable PDK1 to evade SPOP-mediated degradation 67. These alterations promote oncogenesis by enhancing AKT activation. Therefore, the therapeutic potential of PDK1 inhibitors in SPOP-mutant PCa merits further investigation.

#### 5.1.13 Downstream substrates of SPOP associated with cellular stress response

Sequestosome-1 (SQSTM1, p62), a multifunctional autophagy adaptor induced during cellular stress 224, emerges as a critical SPOP substrate. Shi *et al.* demonstrated that cytoplasmic SPOP binds to p62 and triggers its non-degradative ubiquitination at residue K420 within the UBA domain 68. This action reduces p62 puncta formation, liquid phase condensation, dimerization, and ubiquitin-binding capacity, thereby suppressing p62-dependent autophagy 68. SPOP also disrupts p62-mediated Keap1 sequestration, leading to decreased Nrf2-driven transcription of antioxidant genes 68. In PCa, SPOP mutants lose the ability to ubiquitinate p62, instead enhancing autophagy and redox responses in a dominant-negative manner 68. These mechanisms highlight the oncogenic roles of autophagy and Nrf2 activation in SPOP-mutant PCa, making this pathway a promising therapeutic target.

In conclusion, SPOP governs its substrates through ubiquitin-mediated proteasomal degradation or non-degradative ubiquitination. Mutations or reduced expression of SPOP disrupt this regulatory mechanism, leading to substrate dysregulation and affecting various biological processes in cells, driving tumorigenesis and progression in PCa.

### 5.2 Versatile roles of SPOP in tumorigenesis of the breast cancer and gynecologic cancer

**Figure 6:**
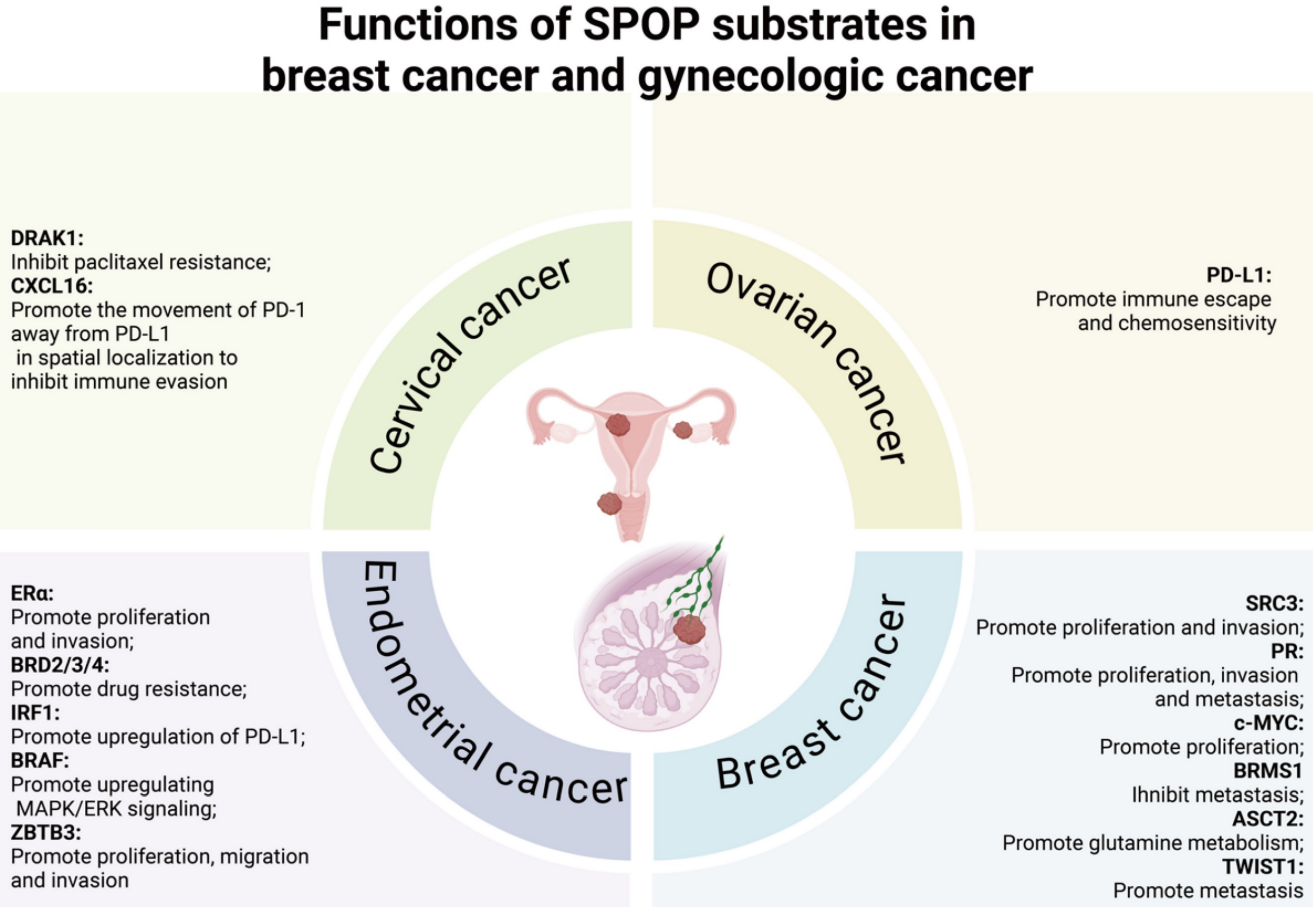
** Functional roles of SPOP substrates in breast cancer and gynecologic cancer.** The figure encapsulates the multifaceted roles of SPOP substrates in the oncogenic processes of breast cancer and gynecologic cancer. By regulating various pathways—ranging from growth factor signaling to immune evasion and hormonal regulation—SPOP substrates are pivotal in determining the aggressiveness and progression of these malignancies. SPOP: Speckle-type POZ protein.

A growing body of research has investigated the role of SPOP in breast cancer and gynecologic cancers, including endometrial, cervical, and ovarian cancers. As can be observed from Table 4 and Figure 6, several SPOP substrates have been identified across these cancer types, including SRC3, progesterone receptor (PR), c-MYC, breast cancer metastasis suppressor 1 (BRMS1), alanine serine cysteine transporter 2 (ASCT2) and twist family BHLH transcription factor 1 (TWIST1) in breast cancer, estrogen receptor α (ERα), BRD2/3/4, B-Raf proto-oncogene (BRAF), zinc finger and BTB domain-containing protein 3 (ZBTB3), and interferon regulatory factor 1 (IRF1) in endometrial cancer, death-associated protein kinase-related apoptosis-inducing kinase 1 (DRAK1) and C-X-C motif chemokine ligand 16 (CXCL16) in cervical cancer, and PD-L1 in ovarian cancer.

#### 5.2.1 Downstream substrates of SPOP in breast cancer

SRC-3/AIB1, also referred to as ACTR/pCIP/TRAM-1/RAC3, was originally identified as a mediator of ER signaling and is often amplified or overexpressed in breast cancer 225. The role of SRC-3 in breast cancer is similar to its role in PCa, with its primary function being the enhancement of gene transcription involved in cell proliferation, survival, and metastasis 226,227. SRC-3 is a coactivator of ER, which is crucial in estrogen-dependent breast cancer 226,227. Li, C *et al.* demonstrated that SPOP orchestrates the ubiquitination and degradation of SRC-3 through a phosphorylation-dependent interaction with an SRC-3 phospho-degron 69. Casein kinase Iɛ phosphorylates Serine 102 within this degron, thereby enhancing SPOP-dependent SRC-3 turnover 69. Genomic analysis of the SPOP locus in breast cancer reveals frequent instances of genomic loss or loss of heterozygosity 69. Furthermore, re-expression of SPOP effectively suppresses SRC-3-driven oncogenic signaling and tumorigenesis, highlighting its role as a tumor suppressor in breast cancer 69. In summary, the SPOP-SRC-3 axis serves as a crucial regulatory mechanism in breast cancer, with therapeutic interventions aimed at restoring this pathway potentially improving outcomes and overcoming resistance in patients.

PR, a protein modulated by estrogen, was established as the first prognostic and predictive biomarker for evaluating response to endocrine therapies 228. Today, it remains the gold standard for identifying functional, targetable estrogen receptors in breast malignancies 228. Recent reports have identified PR as a bona fide substrate for SPOP 70. The SPOP-PR axis plays a critical role in breast cancer by regulating PR protein stability through ubiquitin-dependent degradation 70. SPOP's interaction with PR suppresses PR's activity, including its transactivation potential and downstream signaling effects, such as ERK1/2 activation and S-phase entry 70. This axis highlights a molecular pathway essential for maintaining PR homeostasis, and its disruption—such as through SPOP inactivation—may contribute to breast cancer progression 70. Understanding this axis provides valuable insights into potential therapeutic strategies targeting PR regulation in breast cancer.

*c-MYC* amplification and/or hyperactivation occurs in 20% to 40% of human cancers, including breast cancer, and is often associated with poor clinical outcomes 229. As a transcription factor, c-MYC exerts its oncogenic effects by modulating gene expression programs, both activating and repressing target genes to drive tumor progression 229. c-MYC binds to both LINC01638 and SPOP, with LINC01638 preventing SPOP-mediated ubiquitination and degradation of c-MYC 42. In turn, c-MYC promotes the transcription of metadherin (MTDH), which subsequently activates Twist1 expression, driving EMT 42. Therapeutic strategies targeting this pathway could involve disrupting the c-MYC/LINC01638 interaction to restore SPOP-mediated c-MYC degradation. Alternatively, direct inhibition of c-MYC, MTDH, or Twist1 expression could effectively block downstream signaling, thereby suppressing EMT and limiting tumor progression.

BRMS1, located on chromosome 11q13, was first identified in the 1990s following clinical observations linking deletions in chromosome 11 to increased breast cancer aggressiveness and reduced overall survival in patients 230,231. One possible explanation for BRMS1's metastasis suppression is its interaction with retinoblastoma binding protein 1 (RBP1) and multiple components of the mSin3 histone deacetylases (HDAC) complex, suggesting a role in transcriptional repression mechanisms 232. Additionally, BRMS1 functions as a negative regulator of EGFR, indicating its potential to inhibit breast cancer progression 233. This could represent an additional mechanism by which BRMS1 suppresses metastasis, as demonstrated by the findings of Hurst, Douglas R., *et al* 234. The SPOP-BRMS1 axis plays a crucial role in regulating metastasis by affecting the stability and activity of BRMS1. Through knockdown of SPOP, BRMS1 evades ubiquitin-mediated degradation, which augments its transcriptional repression of metastasis-related genes such as *uPA* and *OPN* 76. This axis holds promise as a therapeutic target, providing insights into novel strategies for inhibiting metastasis in aggressive cancers.

Glutamine, a versatile amino acid with pleiotropic functions, serves as a critical nutrient source for cancer cells, facilitating their rapid proliferation and supporting the maintenance of the tumorigenic phenotype 235. Recent studies have revealed a novel mechanism by which the neddylation inhibitor MLN4924 modulates glutamine metabolism in cancer cells 75. This process involves the inactivation of SPOP, leading to enhanced glutamine uptake 75. Mechanistic investigations show that ASCT2, a major glutamine transporter, is a substrate of SPOP 75. Upon MLN4924 treatment, ASCT2 accumulates, resulting in increased glutamine uptake 75. Notably, glutamine deprivation itself initiates a feedback loop, triggering SPOP self-ubiquitylation and subsequent degradation, which further promotes ASCT2 accumulation 75. This finding underscores the intricate interplay between cellular metabolic states and protein degradation pathways in cancer cells. From a therapeutic perspective, combining MLN4924 with the glutamine metabolism inhibitor V-9302 demonstrated synergistic effects, significantly enhancing cytotoxicity against breast cancer cells both *in vitro* and *in vivo* 75. These results highlight the potential of targeting multiple nodes in the glutamine metabolism pathway for improved anticancer efficacy.

Twist1, a key transcription factor implicated in embryonic development and cancer progression, plays a pivotal role in orchestrating EMT in various malignancies, including breast cancer 236-239. Recent studies have shown that SPOP physically interacts with Twist1, facilitating both K63- and K48-linked ubiquitination, primarily at the K73 residue 114. This ubiquitination marks Twist1 for degradation, which subsequently suppresses EMT processes, including cancer cell migration and invasion 114. When *SPOP* is silenced, Twist1 stability increases, leading to enhanced EMT characteristics 114. This alteration significantly accelerates breast cancer cell migration and invasiveness *in vitro* and promotes lung metastasis *in vivo* 114. These findings suggest that the loss of SPOP contributes to a more aggressive cancer phenotype due to the unregulated activity of Twist1. In conclusion, SPOP's role in ubiquitinating and destabilizing Twist1 is crucial for controlling EMT levels and mitigating aggressive cancer behaviors. This study highlights the therapeutic potential of targeting the SPOP-Twist1 axis in breast cancer treatment strategies.

#### 5.2.2 Downstream substrates of SPOP in endometrial cancer

From outlined in Table 4 and Figure 6, the downstream substrates of SPOP in endometrial cancer include ERα, BRD2/3/4, IRF1, BRAF, and ZBTB3.

ERα, encoded by the *ESR1* gene, belongs to the steroid hormone receptor superfamily and is essential for mediating estrogen-induced proliferation in hormone-responsive cancers, such as endometrial cancer 240. ERα plays a central role in promoting endometrial cancer and serves as a substrate for SPOP. SPOP targets ERα by recognizing its Ser/Thr (S/T)-rich degrons located in the AF2 domain, leading to ERα degradation via the ubiquitin-proteasome pathway 241. Inhibition of SPOP using small interfering RNAs (siRNAs) promotes the proliferation of endometrial cells, indicating its regulatory function 241. Mutations in *SPOP* found in endometrial cancer compromise its ability to mediate ERα degradation and ubiquitination 241. Moreover, SPOP also plays a role in estrogen-driven ERα degradation and transactivation, highlighting its multifaceted involvement in endometrial cancer progression 241. Recent studies have identified *G3BP1* as both highly expressed and frequently mutated in endometrial cancer, with its expression positively correlating with ERα protein levels 242. Mechanistically, *G3BP1* and its mutant variant, the latter characterized by a prolonged half-life, compete with ERα for binding to SPOP 242. This competitive binding interferes with SPOP-mediated ubiquitination and degradation of ERα, resulting in ERα stabilization 242. Functionally, G3BP1 and its mutant enhance endometrial cancer cell proliferation and migration by modulating the G3BP1/SPOP/ERα axis 242. Importantly, the anti-estrogen drug fulvestrant has shown the capacity to reverse the oncogenic effects of G3BP1 and its mutant, highlighting a promising therapeutic avenue.

The BET family proteins—BRD2, BRD3, and BRD4—are established as direct substrates of SPOP, a key regulator in PCa 35. Parallel studies have confirmed this interaction in endometrial cancer 36. Notably, SPOP mutations associated with PCa inhibit BET protein degradation, while mutations associated with endometrial cancer paradoxically enhance BET protein degradation through a gain-of-function mechanism 36. Specifically, endometrial cancer-specific SPOP mutants, which markedly reduce BET protein levels, increase endometrial cancer cells' sensitivity to BET inhibitors by promoting apoptosis and suppressing proliferation. In contrast, overexpression of PCa-specific SPOP mutants, relative to WT SPOP, renders PCa cells more resistant to BET inhibitors 35. This resistance is mitigated by individual or combined knockdown of BET proteins in cells with the SPOP-Y87C mutation 35, underscoring the context-dependent effects of SPOP mutations on BET-targeted therapies. In summary, the differential impact of SPOP mutations on BET protein degradation highlights a context-dependent mechanism that distinctly influences therapeutic responses in prostate and endometrial cancers. While endometrial cancer-specific SPOP mutations enhance BET protein degradation and sensitize cells to BET inhibitors, PCa-specific mutations hinder this degradation, leading to increased resistance. These findings underscore the importance of SPOP mutation profiling in personalizing BET inhibitor therapies, offering a potential strategy for more targeted and effective cancer treatments.

BRAF is a member of the rapidly accelerated fibrosarcoma (RAF) kinase family, which also includes ARAF and CRAF (RAF1) 243. Among these, BRAF exhibits the highest affinity for binding to RAS and demonstrates the greatest activity in phosphorylating MEK1/2, thereby effectively transducing signals downstream of RAS through the MEK-ERK signaling cascade 244. Cytoplasmic SPOP directly interacts with BRAF, promoting its non-degradative ubiquitination and thereby limiting BRAF's association with other essential components of the MAPK/ERK pathway 74. Loss of SPOP function enhances MAPK/ERK activation, a process further exacerbated by endometrial cancer - and PCa-associated SPOP mutations, which show diminished binding and ubiquitination capacity toward BRAF 74. Additionally, cancer-specific mutations within BRAF disrupt its interaction with SPOP, allowing BRAF to evade SPOP-mediated ubiquitination 74. This escape leads to increased MAPK/ERK signaling, thereby intensifying the neoplastic potential and malignant behavior of cancer cells 74. In conclusion, targeting the dysregulation of the SPOP-BRAF interaction presents a potential therapeutic strategy for cancers characterized by aberrant MAPK/ERK signaling. Moreover, therapies aimed at counteracting the effects of cancer-associated BRAF mutations may provide a tailored approach to inhibit oncogenic signaling, offering new opportunities for more effective treatments in cancers with SPOP or BRAF mutations, such as endometrial and PCa.

ZBTB proteins represent a growing family of transcription factors, defined by a DNA-binding zinc finger domain paired with a transcription-repressing BTB/POZ domain 245. These ZBTB proteins are essential in numerous biological processes, including development, cellular differentiation, and oncogenesis, reflecting their importance across normal physiology and disease 246. Among them, ZBTB3 has emerged as a critical regulator of cancer cell proliferation via the reactive oxygen species (ROS) detoxification pathway 246. SPOP selectively recognizes two Ser/Thr (S/T)-rich degrons within ZBTB3, initiating its degradation via the ubiquitin-proteasome pathway 116. However, endometrial cancer -associated SPOP mutants exhibit impaired regulation of ZBTB3 stability 116. Loss of SPOP function consequently promotes endometrial cell proliferation, migration, and invasion, partly through ZBTB3 accumulation 116. Notably, ZBTB3 regulates the transcription of sonic hedgehog (SHH), with SPOP inactivation leading to ZBTB3-dependent SHH upregulation in endometrial cancer cells 116. The small molecule SHH inhibitor RUSKI-43 effectively suppresses cell proliferation, migration, and invasion in endometrial cancer cells lacking functional SPOP or expressing endometrial cancer -associated SPOP mutants 116, underscoring its potential as a therapeutic strategy in SPOP-deficient endometrial cancer. Importantly, by targeting the downstream effects of SPOP loss—particularly the accumulation of ZBTB3 and its upregulation of SHH—these therapies could address the unique oncogenic mechanisms in SPOP-mutant cancers, potentially improving patient outcomes with reduced off-target effects.

IRFs are critical transcription factors within the interferon system, playing key roles in immune response regulation 247. Certain IRFs, such as IRF1, is pivotal as a transcription factor in driving the expression of immune response genes during infection 248. Distinct from other IRFs, IRF1 uniquely promotes the expression of various cell cycle inhibiting factors, thus serving as an important tumor suppressor 248. Recent studies have identified the SPOP as a key mediator of IRF1 proteasomal turnover in both human and mouse cells 248. Specifically, S/T-rich degrons in IRF1 are essential for its degradation through the SPOP MATH domain 248. In the absence of SPOP, elevated levels of IRF1 enhance IRF1-dependent cellular responses, underscoring the critical role of SPOP in regulating IRF1 protein abundance 248. Recently, the SPOP has also been identified as a key negative regulator of the IRF1-PD-L1 axis in endometrial cancer 71. Mechanistically, WT SPOP binds to IRF1, the primary transcription factor governing PD-L1 expression, and facilitates its ubiquitin-proteasomal degradation 71. This interaction suppresses IRF1-mediated transcriptional upregulation of PD-L1, thereby limiting immune evasion. In contrast, endometrial cancer-associated SPOP mutants fail to degrade IRF1 and instead promote its stabilization, leading to enhanced PD-L1 expression 71. Functionally, endometrial cancer-associated SPOP mutations accelerate xenograft tumor growth, partially by augmenting IRF1 and PD-L1 levels 71. These findings highlight the critical roles of IRF1 and PD-L1 in SPOP mutation-driven tumor immune evasion in endometrial cancer and suggest potential targets for immunotherapeutic intervention.

#### 5.2.3 Downstream substrates of SPOP in cervical cancer

Based on the systematic classification presented in Table 4 and the progressive changes captured in Figure 6, SPOP substrates include DRAK1 and CXCL16 in cervical cancer.

DRAK1, or serine/threonine protein kinase 17A (STK17A), is indeed a significant member of the DAP kinase family, which is known for its role in promoting apoptosis and regulating various cellular processes 249. In cervical cancer, recent studies have showed that DRAK1 is identified as a novel antagonist of inflammation that targets TRAF6 for degradation, thereby limiting the progression of advanced cervical cancer mediated by inflammatory signaling 250. Furthermore, the downregulation of DRAK1 expression is associated with paclitaxel resistance in cervical cancer cells 77. In paclitaxel-resistant cells, DRAK1 protein is degraded by the SPOP through K48-linked polyubiquitination-mediated proteasomal degradation, leading to an increase in TRAF6 levels and subsequent TRAF6-mediated NF-κB activation, which promotes tumor progression 77. Targeting this axis may present a novel therapeutic strategy for overcoming drug resistance and inhibiting the advancement of cervical cancer.

In the CXC chemokine family, CXCL16 is a prominent chemokine produced by tumor cells, especially those that infiltrate the tumor microenvironment (TME), where it signals through its receptor, CXCR6 251. Recent studies have shown that myeloid cells promote tumor cell survival via CXCL16-CXCR6 signaling, and targeting this pathway has demonstrated promising efficacy against NK-cell tumors *in vivo* 252. In cervical cancer, SPOP has been identified as a critical regulator that binds to and promotes the degradation of the chemokine CXCL16 72. This interaction profoundly impacts the TME, particularly through the modulation of immune cell dynamics by cancer-associated fibroblasts (CAFs) 72. The degradation of CXCL16 by SPOP disrupts the chemoattractive gradient necessary for the efficient recruitment of immune cells to the tumor site 72. Consequently, this spatial separation impairs the ability of immune cells to effectively locate and attack tumor cells, facilitating immune evasion by the tumor 72. Elucidating the role of SPOP in the regulation of CXCL16 degradation reveals potential therapeutic targets. Strategies aimed at inhibiting SPOP activity or stabilizing CXCL16 levels may enhance the infiltration of immune cells into tumors, thereby augmenting the efficacy of immunotherapies.

#### 5.2.4 Downstream substrates of SPOP in ovarian cancer

In ovarian cancer, PD-L1 positivity has been associated with both poorer and better prognoses in various studies 253,254. Interestingly, in a trial with results published in 2024, PD-L1 expression was not associated with clinical response to Nivolumab in gynecologic cancers 255. Instead, total CD8+ T cell infiltration, as well as an increasing fraction of CD8+PD-1+ and CD8+PD-1+TOX+ T cells, was linked to improved clinical benefit 255. Recent studies have revealed that CRL3 facilitates the degradation of PD-L1 by forming a complex with its adaptor protein SPOP 73. This mechanism suppresses the malignant characteristics of cancer cells, thereby inhibiting the immune escape of ovarian cancer cells and enhancing their sensitivity to chemotherapeutic agents such as cisplatin 73. Therapies targeting the CUL3/SPOP complex-PD-L1 axis hold significant potential for improving treatment outcomes in ovarian cancer. By promoting the degradation of PD-L1, these therapies could effectively suppress cancer cell malignancy, inhibit immune escape, and enhance sensitivity to chemotherapeutic agents like cisplatin.

### 5.3 Tumor-suppressive roles of SPOP in digestive system malignancies

**Figure 7:**
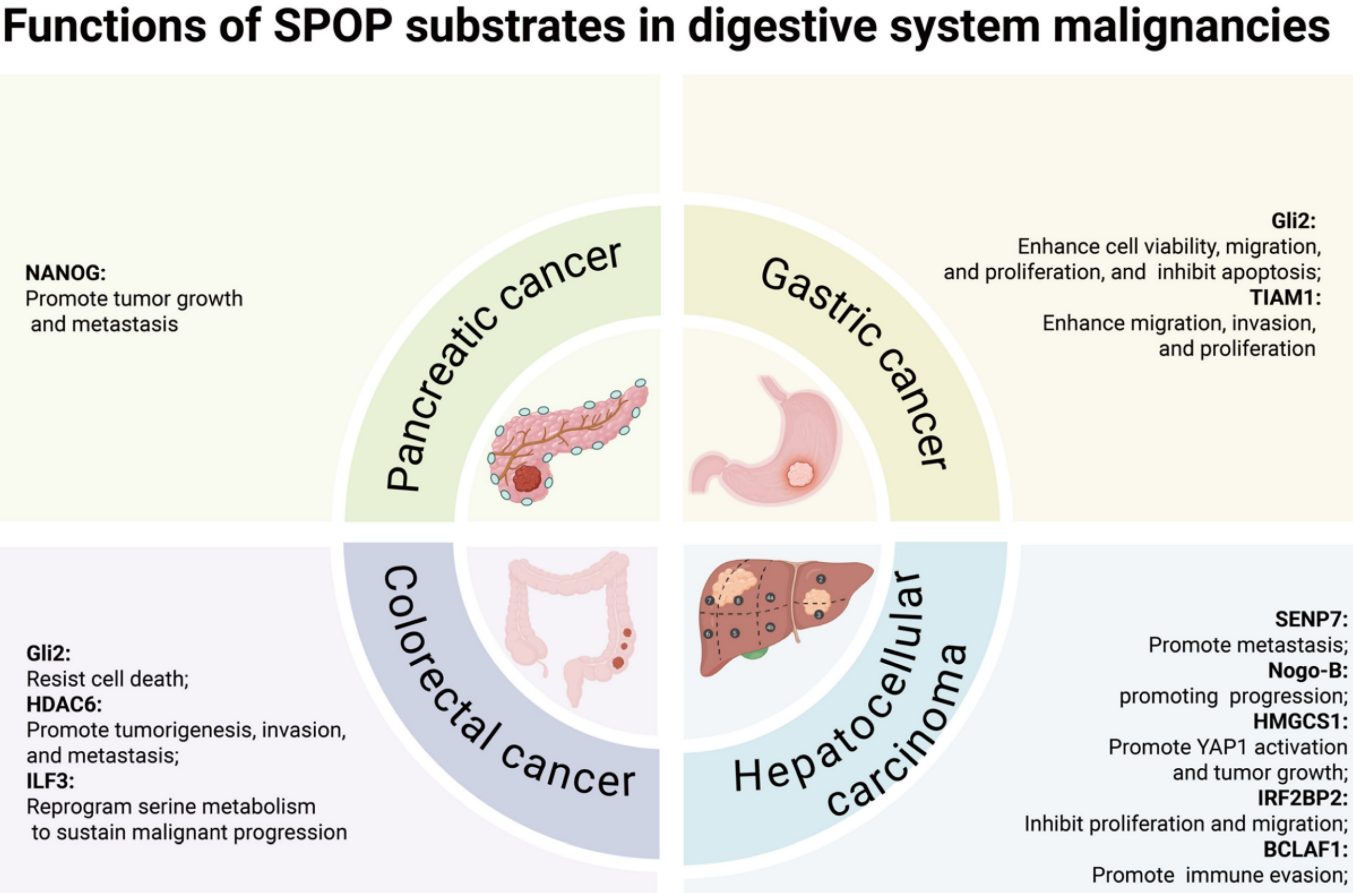
** Functional roles of SPOP substrates in digestive system tumors.** This figure illustrates the diverse roles of SPOP substrates in the oncogenic processes of digestive system cancers. By regulating pathways such as immune evasion, SPOP substrates play a crucial role in the aggressiveness and progression of these tumors. SPOP: Speckle-type POZ protein.

SPOP has been recognized as a critical tumor suppressor in various malignancies, including those of the digestive system. As represented in Table 4 and Figure 7, SPOP plays a crucial role in the degradation of several oncogenic proteins including SENP7, Nogo-B, 3-hydroxy-3-methylglutaryl-CoA synthase 1 (HMGCS1), interferon regulatory factor 2-binding protein 2 (IRF2BP2), and B cell lymphoma-2-associated transcription factor 1 (BCLAF1) in HCC; Gli2, HDAC6, and ILF3 in CRC; Gli2 and T lymphoma invasion and metastasis 1 (TIAM1) in GC; Nanog in pancreatic cancer.

#### 5.3.1 Downstream substrates of SPOP in HCC

The reversible post-translational modification of proteins by small ubiquitin-related modifier (SUMO), termed SUMOylation, is tightly regulated by SENPs 256. SENP7, a member of the SENP family, has been implicated in various critical cellular processes, including tumorigenesis 206, DNA repair 207, cytosolic DNA sensing 60, and lipid metabolism 257. As previously demonstrated, SPOP orchestrates the ubiquitin-dependent proteolysis of SENP7 during cellular senescence in PCa 60. In the context of HCC, studies suggest that SPOP recognizes and binds to SENP7, facilitating its degradation via ubiquitin-dependent proteolysis 79. Immunohistochemical analysis indicates that vimentin expression is negatively correlated with SPOP and positively correlated with SENP7 79. Consequently, the increased degradation of SENP7 due to SPOP overexpression leads to reduced vimentin levels, which in turn attenuates HCC cell metastasis 79. In conclusion, targeting the SPOP-SENP7 pathway presents a promising therapeutic strategy for HCC. Developing inhibitors or modulators that specifically alter SPOP activity or SENP7 stability could pave the way for novel treatments aimed at mitigating the metastatic spread of HCC.

Nogo-B, also known as reticulon-4B (RTN-4B), is a member of the reticulon protein family that is predominantly localized in the endoplasmic reticulum 258-260. It plays a critical role in maintaining the tubular structure and function of the endoplasmic reticulum 258-260. Nogo-B is widely expressed in various tissues, including the liver 261, kidney 262, and lung 263. With respect to its biological functions, Nogo-B is crucial in vascular remodeling 264, cell migration and proliferation, as well as the EMT 265. A recent study has uncovered a novel mechanism by which Nogo-B contributes to the progression of HCC. Specifically, in HCC, SPOP is highly O-GlcNAcylated by O-GlcNAc transferase (OGT) at Ser96, which enhances the nuclear localization of SPOP in hepatoma cells 78. This nuclear positioning attenuates the ubiquitination of the Nogo-B protein, thereby promoting HCC progression both *in vitro* and *in vivo* 78. Furthermore, the ablation of O-GlcNAcylation through an S96A mutation increased the cytoplasmic localization of SPOP, which in turn inhibited the Nogo-B/c-FLIP cascade and impeded HCC progression 78. These findings suggest that targeting the OGT/SPOP/Nogo-B axis could represent a promising therapeutic strategy for HCC.

HMGCS1 is a pivotal cytoplasmic enzyme in the lanosterol biosynthesis pathway, responsible for catalyzing the conversion of acetoacetyl-CoA to 3-hydroxy-3-methylglutaryl-CoA (HMG-CoA) 266. The expression of HMGCS1 was associated with the malignant progression in multiple cancers including HCC 267. A recent study revealed that SPOP interacts with HMGCS1 and facilitates its polyubiquitination, leading to its degradation 84. Conversely, CSN6 antagonizes the ubiquitin ligase activity of SPOP, thereby stabilizing HMGCS1, which in turn activates YAP1 to drive tumor growth 84. In orthotopic liver cancer models, targeting both CSN6 and HMGCS1 effectively suppresses tumor growth under both normal and high-fat diet conditions 84. Furthermore, depleting *HMGCS1* significantly enhances the efficacy of YAP inhibitors in patient-derived xenograft models 84. These findings suggest that therapeutic strategies aimed at the CSN6-SPOP-HMGCS1 axis hold potential for cancer treatment. Inhibiting CSN6 or enhancing SPOP activity could promote HMGCS1 degradation, thereby diminishing YAP1 activation and subsequent tumor growth. Moreover, combining HMGCS1 depletion with YAP inhibitors could further potentiate therapeutic outcomes. Such approaches indicate that modulating this axis could provide effective treatment options for HCC.

IRF2BP2 was initially identified as a transcription corepressor of IRF-2 268-270. It regulates the expression of various genes involved in oncogenic processes such as cell proliferation, metastasis, and immune response 268-270. Recent research has revealed that IRF2BP2 is a substrate of SPOP 37. Studies have shown that SPOP facilitates IRF2BP2 ubiquitination through a CUL3-dependent mechanism 37. From a functional perspective, IRF2BP2 was found to inhibit the proliferation and migration of HCC cells, an effect that could be reversed by co-expressing SPOP 37. Interestingly, an HCC-derived mutant, SPOP-M35L, demonstrated enhanced interaction with IRF2BP2 37. In line with this observation, SPOP-M35L exhibited a more potent ability to ubiquitinate and degrade IRF2BP2 compared to its WT counterpart 37. Unlike WT SPOP, the SPOP-M35L variant was capable of promoting HCC cell proliferation and migration, possibly due to its higher affinity for IRF2BP2 37. These findings suggest that the M35L mutation effectively transforms SPOP from a tumor suppressor into an oncoprotein. This discovery provides new insights into the molecular mechanisms underlying HCC progression and may have implications for developing targeted therapies for this type of cancer.

BCLAF1 was initially identified as a protein that interacts with anti-apoptotic members of the Bcl2 family; however, it has since been linked to various biological processes, including the regulation of transcription 271. Recent studies have demonstrated that BCLAF1 competitively inhibits the SPOP-mediated ubiquitination and degradation of PD-L1 by interacting with SPOP, thereby sustaining PD-L1 expression 86. This mechanism ultimately promotes immune evasion and tumor progression in HCC 86. Additionally, BCLAF1 has been identified as a potential therapeutic target, with the efficacy of immune checkpoint blockade (ICB) treatment potentially enhanced in HCC cases exhibiting high BCLAF1 expression *in vitro* 86. In conclusion, Targeting the SPOP-BCLAF1 axis may enhance the efficacy of immunotherapy in HCC.

Importantly, recent research has confirmed that SPOP functions as a tumor suppressor in hepatoblastoma (HB) development via the PI3K/Akt pathway, with its anti-cancer activity impaired by the S119N mutation 272. Furthermore, solute carrier family 7 member 1 (SLC7A1) has been identified as a potential substrate of SPOP, contributing to HB progression through the disruption of arginine metabolism 272.

#### 5.3.2 Downstream substrates of SPOP in CRC

As previously noted, GLI zinc-finger transcription factors serve as the final effectors of the Hh signaling pathway, with GLI1 and GLI2 generally acting as positive regulators and GLI3 often functioning as a negative regulator 146. In CRC, the expression levels of Hh pathway proteins vary considerably across different studies 273. A study has shown that SPOP interacts with Gli2, facilitating its ubiquitination and subsequent degradation 80. This interaction leads to a reduction in the expression of Bcl-2, an apoptotic protein associated with the Hh/Gli2 pathway, thereby impairing its function in preventing cell death in CRC 80. The SPOP-Gli2 axis, therefore, plays a critical role in maintaining the balance between cell survival and death, and its dysregulation could offer potential therapeutic targets for cancer treatment. This is different from the role of GLI zinc-finger transcription factors in PCa, where GLI3 is upregulated and acts as a substrate of SPOP 45.

HDAC6, a member of the HDAC family, is an enzyme involved in the dynamic regulation of the deacetylation of both histone and non-histone substrates 274. In CRC, HDAC6 expression is elevated in tumor tissue relative to adjacent non-cancerous tissue and is frequently linked to poor disease prognosis 275. A study reported that SPOP specifically interacts with HDAC6, promoting its polyubiquitination and subsequent degradation in cells 81. Notably, cancer-derived SPOP mutants disrupt this interaction, preventing HDAC6 degradation 81. Furthermore, increased cellular proliferation and migration observed in SPOP-depleted HCT116 colon cancer cells could be partially reversed by additional depletion of HDAC6, suggesting that HDAC6 is a key downstream effector of SPOP's tumor suppressor function 81. Together, these findings establish SPOP as an upstream negative regulator of HDAC6 stability. Loss-of-function mutations in SPOP may lead to elevated levels of the HDAC6 oncoprotein, which could promote tumorigenesis and metastasis in CRC, highlighting the potential for targeted therapies aimed at this axis.

ILF3, also referred to as NF90/NF110, encodes a double-stranded RNA (dsRNA)-binding protein that associates with proteins, mRNAs, small noncoding RNAs, and dsRNAs to regulate gene expression and enhance mRNA stability 276,277. Recent studies have shown that ILF3 is overexpressed in CRC and serves as a prognostic marker associated with poor survival, by reprogramming serine metabolism to sustain malignant progression 85. Mechanistic investigations revealed that the EGF-MEK-ERK signaling pathway is responsible for the phosphorylation of ILF3, which in turn inhibits the SPOP-mediated polyubiquitination and subsequent degradation of ILF3 85. Notably, the combination of the Serine-Glycine-One-Carbon (SGOC) inhibitor and the anti-EGFR monoclonal antibody cetuximab effectively suppresses the growth of patient-derived xenografts characterized by elevated levels of ERK and ILF3 85.

#### 5.3.3 Downstream substrates of SPOP in GC

As previously reported, GLI2 has been recognized as a substrate of SPOP, which mediates its proteasomal degradation in CRC. Recent studies have shown that GLI2 is significantly upregulated in GC, with high GLI2 expression correlating with poor survival outcomes 278. Recent studies have shown that high SPOP expression is negatively correlated with lymph node metastasis, poor histological differentiation, and tumor malignancy according to TNM staging 117. *In vitro*, SPOP overexpression suppressed cell proliferation, migration, and colony formation in GC cell lines, whereas SPOP knockdown enhanced cell viability, migration, and proliferation, while inhibiting apoptosis 117. Mechanistically, SPOP promoted Gli2 degradation without impacting its synthesis 117. Furthermore, in MKN45 cells, elevated SPOP expression was associated with a significant reduction in cytoplasmic Gli2 levels 117. These results indicate that SPOP plays a critical role in suppressing gastric tumorigenesis by inhibiting the Hh/Gli2 signaling pathway. This suggests that SPOP may serve as a potential target for the development of therapeutic strategies for GC in the future.

TIAM1 is a member of the Rac-specific guanine nucleotide exchange factor (GEF) family, with its primary function being the activation of RAC1 through the exchange of guanosine diphosphate (GDP) for guanosine triphosphate (GTP) 279. This activation triggers downstream RAS signaling pathways that regulate processes such as cytoskeletal remodeling, cell adhesion, migration, proliferation, and apoptosis 279. In 2024, a study has indicated that SPOP selectively interacts with TIAM1, facilitating its ubiquitination and degradation 83. Importantly, the disruption of SPOP-mediated degradation of TIAM1 enhances the migration, invasion, and proliferation of GC cells 83. Additionally, a strong correlation between TIAM1 and SPOP expression was observed in both GC tissues and adjacent normal tissues 83. Ultimately, dysregulation of the SPOP-TIAM1 axis may contribute to the uncontrolled growth and metastasis of GC, making it a potential therapeutic target.

#### 5.3.4 Downstream substrates of SPOP in pancreatic cancer

As noted earlier, Nanog is a substrate of SPOP in PCa, where it facilitates Nanog polyubiquitination and subsequent degradation, thereby regulating the stem cell characteristics of PCa cells. In the same year, researchers have also found that SPOP functions as a tumor suppressor in pancreatic cancer, where it was found to be downregulated in most patients, with low expression levels correlating with poor prognosis 82. Knockdown of SPOP in pancreatic cancer cell lines SW1990 and PANC-1 significantly enhanced cell proliferation, migration, and invasion, effects linked to the upregulation of proteins involved in cell cycle progression and EMT 82. This oncogenic activity was further associated with decreased ubiquitination and degradation of NANOG 82. Moreover, the patient-derived SPOP mutation Q360* impaired its nuclear localization, leading to NANOG accumulation in the nucleus, thereby driving tumor growth and metastasis 82. Targeting the SPOP-NANOG axis presents a promising therapeutic strategy for pancreatic cancer. Given that SPOP functions as a tumor suppressor and regulates NANOG degradation, restoring or mimicking SPOP activity could prevent NANOG accumulation and its subsequent oncogenic effects, including enhanced proliferation and metastasis.

### 5.4 Tumor-suppressive roles of SPOP in other malignancies

**Figure 8:**
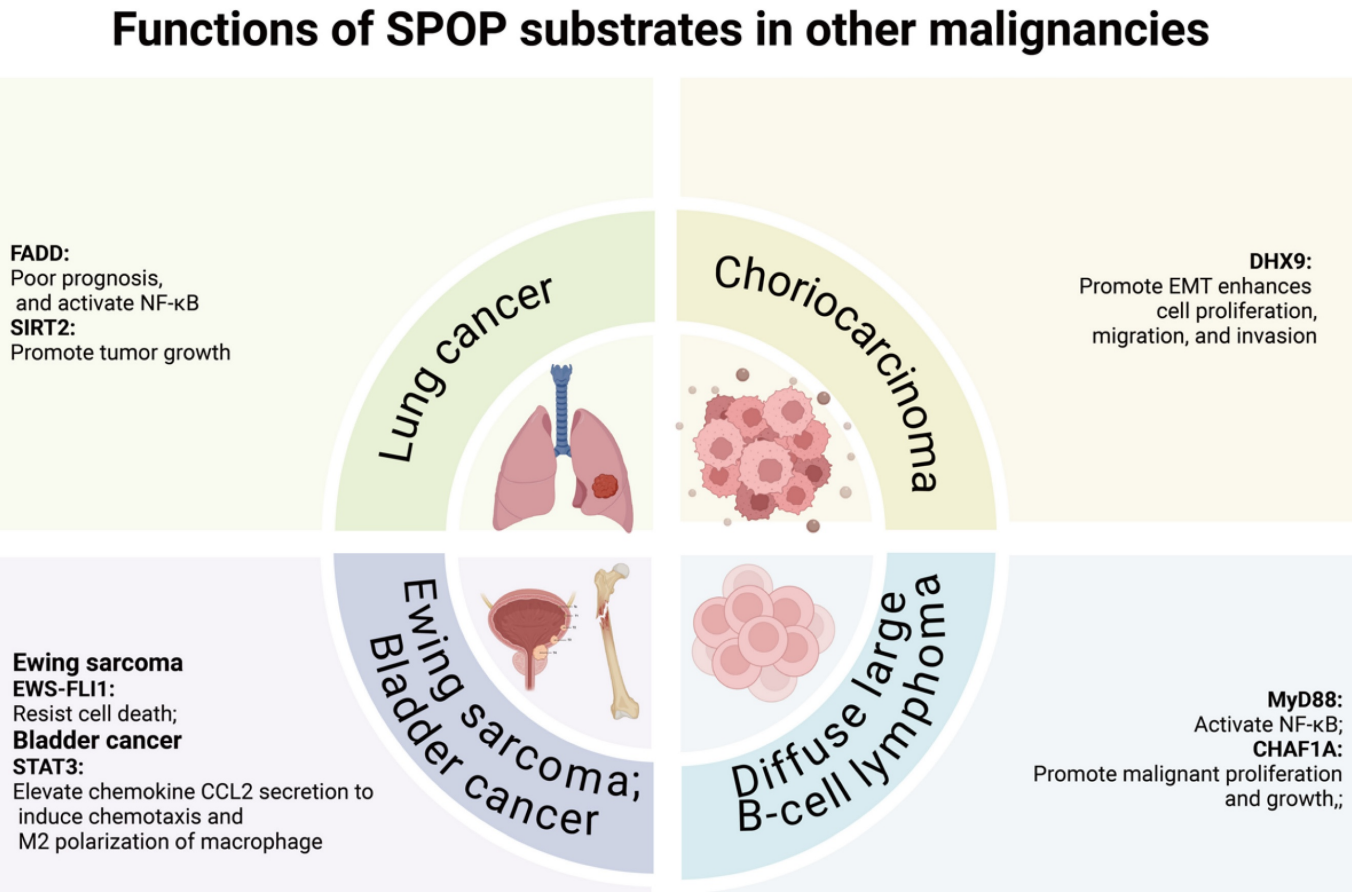
** Functional roles of SPOP substrates in other tumor types.** This figure illustrates the diverse roles of SPOP substrates in various cancers, including lung cancer, DLBCL, choriocarcinoma, Ewing sarcoma, and bladder cancer. By regulating pathways such as signaling and immune evasion, SPOP substrates play a crucial role in the aggressiveness and progression of these tumors. DLBCL: diffuse large B-cell lymphoma; SPOP: Speckle-type POZ protein.

Both the Table 4 and Figure 8 indicate that SPOP suppresses tumorigenesis in various human malignancies, extending beyond PCa, gynecological tumors, and digestive system cancers. These include lung cancer, diffuse large B-cell lymphoma (DLBCL), choriocarcinoma, and Ewing sarcoma. Notably, SPOP exerts its effects through the regulation of key factors such as FAS-associated death structural domain (FADD) and SIRT2 in lung cancer, MyD88 and chromatin assembly factor 1 subunit A (CHAF1A) in DLBLC, DHX9 in choriocarcinoma, EWS-FLI1 in Ewing sarcoma, and signal transducers and transcriptional activators 3 (STAT3) in bladder cancer.

#### 5.4.1 Downstream substrates of SPOP in lung cancer

FADD is a key adaptor protein that transmits apoptotic signals from primary death receptors. In addition to its crucial role in cell death, FADD is also involved in proliferation, cell cycle progression, tumorigenesis, inflammation, innate immunity, and autophagy 280,281. A recent study revealed that elevated FADD protein levels correlate with poor prognosis in NSCLC patients, with its expression primarily regulated by the 26S proteasome 87. SPOP binds FADD and facilitates its degradation, a process that can be blocked by MG132 treatment 87. Notably, SPOP inhibits NF-κB activity and the expression of its target genes via FADD 87. Targeting the SPOP-FADD axis presents a promising therapeutic strategy in lung cancer.

SIRT2, an NAD(+)-dependent protein deacetylase targeting histone H4 lysine 16, p53, and α-tubulin, is essential for mitotic progression and regulates checkpoint functions during early metaphase to ensure chromosomal stability 282. A previous study found that SPOP levels were significantly reduced, while SIRT2 levels were markedly elevated in NSCLC cell lines compared to normal bronchial epithelial cells and in NSCLC specimens compared to paired non-tumor lung tissues 88. SIRT2 is a substrate of SPOP, with SPOP binding to SIRT2 and mediating its degradation 88. Mutations in the MATH domain (G75L and G132R) and BTB domain (G192A and K279N) of SPOP in NSCLC impair its ability to degrade SIRT2 and suppress NSCLC cell growth, highlighting a strong correlation between SPOP's degradation of SIRT2 and its role in inhibiting NSCLC cell proliferation 88. By modulating the SPOP-SIRT2 interaction or enhancing SPOP activity, it may be possible to restore the degradation of SIRT2, thereby inhibiting tumor progression. This approach could offer a novel avenue for therapeutic intervention in NSCLC and potentially other cancers where this axis is dysregulated. Further research into specific inhibitors or activators of the SPOP-SIRT2 pathway could provide valuable tools for targeted cancer treatment.

#### 5.4.2 Downstream substrates of SPOP in DLBLC

MyD88 is an adaptor protein that plays a key role in the innate immune response and inflammatory signaling 283. It is activated by members of the Toll-like receptor (TLR) and interleukin-1 receptor (IL-1R) families 283. Recent studies have revealed that SPOP negatively regulates NF-κB signaling by binding to MyD88 and facilitating its nondegradative ubiquitination 90. Mutations in MyD88 (S149G, S149I, S150I) or in the MATH domain of SPOP (F102I, D140H), commonly associated with DLBCL, disrupt the SPOP-MyD88 interaction and inhibit MyD88 ubiquitination 90. As a result, these mutations drive aberrant MyD88/NF-κB activation in DLBCL 90. Targeting the SPOP-MyD88-NF-κB axis holds therapeutic potential, particularly in cancers like DLBCL where mutations disrupt this pathway.

CHAF1A, the largest subunit of the chromatin assembly factor-1 (CAF-1) complex, is crucial for nucleosome assembly on newly synthesized DNA 284. In DLBCL, studies have shown that CHAF1A is overexpressed and plays a key role in promoting malignant proliferation and growth 89. SPOP acts as a negative regulator of CHAF1A by binding to it and inducing its ubiquitin-mediated degradation 89. Mutations in SPOP or its downregulation, commonly observed in DLBCL, lead to CHAF1A accumulation, which in turn enhances tumor autophagy in a TFEB-dependent manner 89. Targeting the SPOP-CHAF1A axis presents a promising therapeutic strategy for DLBCL. Inhibiting the CHAF1A-TFEB signaling pathway may further suppress tumor growth and survival. Therapeutic approaches such as small molecules or gene therapies designed to modulate this axis could provide novel treatment options for DLBCL patients, especially those with SPOP mutations or low SPOP expression.

#### 5.4.3 Downstream substrates of SPOP in choriocarcinoma

DHX9, formerly known as DNA helicase II and RNA helicase A, is a critical component of the RNA polymerase II (Pol II) holoenzyme, involved in co-transcriptional pre-mRNA processing 285. In choriocarcinoma, studies have shown that reduced SPOP expression enhances cell proliferation, migration, and invasion by promoting EMT 91. These findings further suggest that SPOP acts as a negative regulator of choriocarcinoma progression by binding to DHX9 and inducing its ubiquitin-mediated degradation 91. Targeting the SPOP-DHX9 axis presents a promising therapeutic strategy for choriocarcinoma and potentially other cancers.

#### 5.4.4 Downstream substrates of SPOP in Ewing sarcoma

Ewing sarcoma is a malignancy of bone and soft tissue that predominantly affects children and young adults 286,287. It is driven by a chromosomal translocation that fuses the *EWS* gene with an ETS family transcription factor, most commonly *FLI1*. The resulting *EWS-FLI1* fusion protein is the key oncogenic driver of the disease 286,287. A recent study has identified SPOP as the bona fide E3 ligase that regulates the turnover of EWS-FLI1 in Ewing sarcoma 92. Phosphorylation of the VTSSS degron within the FLI1 domain by Casein kinase 1 enhances SPOP-mediated degradation of EWS-FLI1 92. In contrast, OTUD7A deubiquitinates and stabilizes EWS-FLI1 92. Knockdown of OTUD7A in Ewing sarcoma cell lines reduces EWS-FLI1 levels and inhibits tumor growth both *in vitro* and *in vivo* 92. In conclusion, targeting the SPOP/OTUD7A-EWS-FLI1 axis offers a promising therapeutic strategy for Ewing sarcoma, particularly in cases driven by the EWS-FLI1 fusion protein.

#### 5.4.5 Downstream substrates of SPOP in bladder cancer

STAT, as a family of cytoplasmic transcription factors, responds to stimuli such as cytokines, growth factors, and hormones, transmitting extracellular signals to various organelles within the cell 288. STAT3 plays a key role in promoting cell cycle progression, proliferation, migration, and invasion across various cancer types, including bladder cancer 289. In 2024, researchers identified STAT3 as a novel substrate of SPOP, revealing that SPOP deficiency increased STAT3 protein stability and elevated the secretion of chemokine CCL2, which induced macrophage chemotaxis and M2 polarization 93. In co-cultured macrophages, IL-6 secretion promoted bladder cancer cell proliferation and stemness 93. Furthermore, the transcription factor VEZF1 was found to directly activate SPOP transcription, and its overexpression suppressed these effects in bladder cancer cells 93. Targeting this crosstalk may provide a promising therapeutic strategy for patients with bladder cancer harboring *SPOP* deficiency.

### 5.5 SPOP's oncogenic role in KC

**Figure 9:**
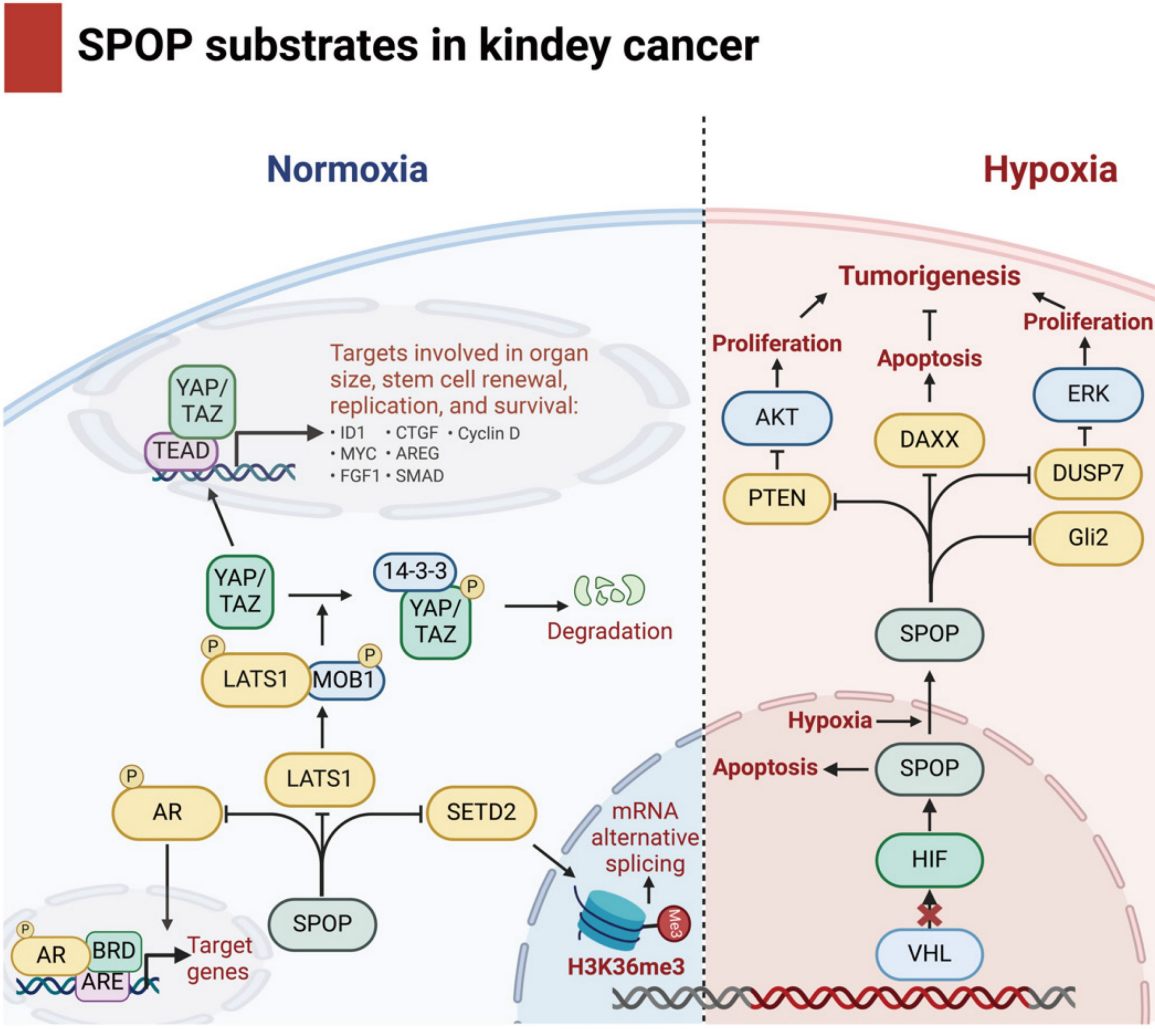
** Potential oncogenic roles of SPOP in KC.** The SPOP contributes to oncogenesis in KC by targeting multiple substrates. Specifically, the cytoplasmic accumulation of SPOP promotes the ubiquitination and degradation of Daxx, DUSP7, Gli2, and PTEN, enhancing cell proliferation and inhibiting apoptosis. Additionally, SPOP mediates the ubiquitination and degradation of SETD2, resulting in decreased H3K36me3, which may facilitate renal carcinogenesis. Furthermore, cytoplasmic SPOP prevents the degradation of the AR in the nucleus, leading to the activation of AR-driven pathways and the progression of KC. AR: androgen receptor; H3K36me: Trimethylation of histone H3 lysine 36; Kidney cancer: KC; SETD2: SET domain-containing 2; SPOP: Speckle-type POZ protein.

No mutations in *SPOP* have been detected in KC to date. The experimental outcomes documented in Table 4 and Figure 9 showed that SPOP plays an oncogenic role in kidney tumorigenesis by targeting key tumor suppressors, including AR, Daxx, DUSP7, Gli2, PTEN, SETD2, and LATS1, and these proteins are essential for regulating cellular processes such as cell proliferation, the cell cycle, and apoptosis.

#### 5.5.1 Downstream substrates of SPOP in KC

As mentioned above, AR is a key factor driving PCa progression. Similarly, studies have shown that targeting AR in both RCC cells (HKC-5, 786-O, 786-P, and SW839) and xenografts (HKC-5 and 786-O) inhibits cell migration and invasion by modulating HIF2a/VEGF signaling by recruiting vascular endothelial cells 290. Accumulating evidence suggests that AR functions as an oncoprotein in RCC, with SPOP inhibiting KC tumorigenesis and progression by targeting AR 94. In an RCC patient-derived xenograft model of acquired resistance to the receptor tyrosine kinase inhibitor (RTKi) sunitinib, AR expression was significantly elevated 94. Similarly, AR levels were increased in RCC cell lines with either acquired or intrinsic sunitinib resistance *in vitro* 94. Sunitinib-induced AR transcriptional activity was associated with increased phosphorylation of serine 81 (pS81) on AR, leading to its nuclear translocation 94. Notably, enzalutamide induced degradation of the phosphorylated AR-SPOP complex, restoring sunitinib sensitivity *in vivo* and promoting tumor regression in the 786-O model 94. In sunitinib-resistant UMRC2 RCC cells, pharmacological inhibition of the proteasome or SPOP ablation via siRNA prevented the degradation of AR induced by enzalutamide 94. These findings underscore the potential of targeting the SPOP-AR axis as a novel approach to improve treatment outcomes in RCC, particularly for patients with acquired resistance to current therapies.

The hypoxic response plays a crucial role in the tumorigenesis of most solid tumors, particularly in RCC 291. Hypoxia, or low oxygen levels within tumors, triggers adaptive responses that promote tumor growth, metastasis, and resistance to therapy 292. In RCC, hypoxia-induced signaling pathways, such as the HIF pathway, are central to these processes 291. These pathways regulate critical factors involved in angiogenesis, metabolism, and cell survival, making them key drivers of KC progression 291. One study demonstrated that in RCC, hypoxia leads to the accumulation of SPOP in the cytoplasm, where it exerts anti-apoptotic and pro-proliferative effects 31. This is achieved by promoting the ubiquitination and degradation of key tumor suppressors, including Daxx, the ERK phosphatase DUSP7, Gli2, and PTEN 31. A significant inverse correlation between PTEN levels and SPOP levels was observed in 100% (14/14) of primary ccRCC tumor samples examined 31. *In vivo* experiments further supported this, where subcutaneous injection of stably transfected HEK293-SPOP-cyto cells into nude mice resulted in tumor formation in approximately 80% (15/19) of the mice within 6 weeks 31. In contrast, WT SPOP and control empty vector cells did not induce tumor growth (0/19 in both cases) 31. These findings suggest that SPOP may have an oncogenic role in RCC, potentially due to its accumulation in the cytoplasm, which impairs its ability to promote the ubiquitination and degradation of substrates typically regulated in the nucleus.

SETD2 primarily catalyzes the trimethylation of histone H3 at lysine 36 (H3K36me3) from the dimethylated form (H3K36me2) within gene bodies, thereby facilitating transcription elongation 293. It has also been identified as a potential tumor suppressor in several human cancers, including RCC 294. One study demonstrated that SPOP directly interacts with SETD2, thereby modulating SETD2 activity on a broad range of genes in HEK293 95. This pathway is particularly important for regulating splicing through the modulation of H3K36me3 levels within the cell 95. The events regulated by SETD2 and SPOP encompass various forms of alternative splicing, with a predominant effect on exon exclusion, thereby highlighting the role of PTB in the alternative splicing process controlled by both SPOP and SETD2 95. In conclusion, the SPOP-SETD2 axis plays a crucial role in regulating gene expression and alternative splicing in cells. Through its influence on SETD2, SPOP regulates H3K36me3 levels, which are essential for proper splicing, with a notable effect on exon exclusion. Given the significance of SETD2 as a tumor suppressor and SPOP's role in regulating splicing, targeting this axis may offer new therapeutic opportunities in cancer treatment, particularly in cancers like RCC where both proteins are implicated in tumorigenesis and drug resistance.

The Hippo/Warts (Mst/Lats) pathway is a critical signaling cascade that regulates organ size and tissue growth during embryonic development. It controls the activity of genes involved in cell differentiation, proliferation, and survival through a kinase-driven mechanism. As illustrated in Figure 9, the Mst1 and Mst2 kinases (orthologs of *Drosophila* Hippo), in complex with Sav1, activate Lats1 and Lats2 (orthologs of *Drosophila* Warts) via phosphorylation 295. In turn, these Lats kinases phosphorylate the transcriptional coactivators Yap and Taz (orthologs of *Drosophila* Yorkie), sequestering them in the cytoplasm and inhibiting their activity 295. Recent studies have demonstrated that the deletion of *Lats1/2* in adult kidney epithelium leads to the development of renal cell carcinoma (RCC), suggesting that LATS1 functions as a tumor suppressor that negatively regulates tumor progression 296. One study identified LATS1, a key component of the Hippo tumor suppressor pathway, as a novel ubiquitin substrate of SPOP 96. Mechanistically, SPOP specifically interacted with LATS1, promoting its polyubiquitination and subsequent degradation in a degron-dependent manner 96. Overexpression of SPOP enhanced cell proliferation, partly by regulating cell cycle distribution, in both 786-O and A498 KC cells. Additionally, SPOP facilitated KC cell invasion by degrading LATS1 96.

In conclusion, SPOP plays a pivotal oncogenic role in KC, particularly in RCC, by regulating key tumor suppressors and modulating critical cellular processes. SPOP facilitates tumorigenesis by targeting and promoting the degradation of tumor suppressors like LATS1, PTEN, SETD2, and others, thus disrupting important signaling pathways such as the Hippo, PI3K/Akt, and cell cycle regulation pathways. Through these actions, SPOP enhances cell proliferation, invasion, and survival, contributing to tumor growth and metastasis. The dysregulation of SPOP-mediated substrate degradation may also be involved in resistance to therapy. As such, targeting SPOP or its downstream effects offers a promising therapeutic avenue for treating KC, particularly for patients with aggressive or resistant forms of the disease.

## 6. SPOP-Targeting Strategies

### 6.1 SPOP as a therapeutic target

Given the dual roles of SPOP as both an oncogene and tumor suppressor in a cancer type-specific manner, the development of SPOP-targeting agents may prove crucial for the treatment of diverse cancers. Structurally, the SPOP protein selectively interacts with specific substrates via its N-terminal MATH domain, which recognizes the SBC motif 33. As previously noted, SPOP is overexpressed and mislocalized in the cytoplasm of nearly all ccRCC, a condition that may drive cellular proliferation and contribute to kidney tumorigenesis 31. A structure-based design, followed by hit optimization, facilitated the identification of small molecules that inhibit the SPOP-substrate protein interaction, thereby disrupting oncogenic SPOP signaling 297. Computational screening, integrating pharmacophore modeling and molecular docking, led to the selection of 109 compounds from the SPECS database, which contains over 200,000 drug-like molecules 297. Compound 6a was identified as a promising hit, effectively competing with the puc_SBC1 peptide for SPOP binding 297. Further chemical optimization produced the more potent compound 6b. Inhibitors 6a, 6b, and soluble compound 6b-HCl significantly disrupted SPOP binding to PTEN and DUSP7 in a dose-dependent manner, whereas compound 6c did not affect these interactions 297. Both 6a and 6b exhibited notable inhibitory effects on the proliferation of the ccRCC A498 cell line 297. Subsequently, the research team continued their investigation and, in 2020, established a structure-activity relationship for 6b analogues as SPOP inhibitors 298. Compound 6lc was found to significantly inhibit colony formation in both A498 and OS-RC-2 cell lines, outperforming previously reported 6b and other tested analogues 298. Various assays confirmed that 6lc directly interacts with the SPOP protein both *in vitro* and in cell lysates. Further mechanistic studies revealed that compound 6lc disrupts the SPOP-substrate protein interaction in ccRCC cell lines, leading to the stabilization and accumulation of tumor suppressors PTEN and DUSP7, while reducing the levels of phosphorylated AKT and ERK downstream 298. Furthermore, SPOP interacts with the cullin 3-RING box 1 scaffold protein through its C-terminal BTB domain, promoting SPOP dimerization and enhancing the ubiquitination activity of the E3 ligase 109. Disrupting the BTB-cullin 3 interaction or inhibiting SPOP dimerization with small molecules could therefore provide a promising strategy for RCC therapy. Notably, the role of SPOP protein varies depending on the cancer context, highlighting the need for future studies to focus on developing cancer treatments that are specific to particular tissues or cell types.

### 6.2 SPOP Ligand-Based PROTACs

Proteolysis-targeting chimeras (PROTACs) are a leading class of agents used for targeted protein degradation (TPD). A PROTAC molecule consists of three components: a ligand for an E3 ubiquitin ligase, a linker, and a ligand for the protein of interest (POI) 299. This structure facilitates the polyubiquitination and subsequent degradation of the POI through the action of the E3 ligase and the UPS 299. Certain E3 ubiquitin ligases recognize specific degradation signals, known as "degrons," which were originally used as ligands for the POI in PROTAC design. Studies have revealed that SPOP substrates contain one or more SBC motifs 33, positioning SPOP as a promising target for developing PROTACs to treat RCC with SPOP overexpression. In 2025, Deng* et al.* presented a bridged PROTAC strategy and successfully developed a proof-of-concept PROTAC degrader, 9 (MS479), which recruits the E3 ligase SPOP by directly binding its substrate GLP as a bridging protein 300. This approach facilitates the polyubiquitination and subsequent degradation of BRD4/3/2 via the 26S proteasome 300. Compound 9 notably reduced the protein levels of the BRD4 short isoform in a time-, concentration-, GLP-, SPOP-, and UPS-dependent manner. Additionally, it effectively suppressed the proliferation of CRC cells 300. Similar strategies may be extended to other cancer types. The bridged PROTAC approach holds promise for targeting E3 ligases that lack small-molecule binders but can interact with substrate proteins amenable to small-molecule binding. Notably, cereblon (CRBN) is, to date, the most widely employed E3 ligases in PROTAC development, with all PROTACs currently in clinical trials relying on CRBN, Hippel-Lindau (VHL), or CRL4^DCAF15^ 301. Consequently, the emergence of acquired resistance to PROTACs that target VHL or CRBN has been observed 302. Future research should therefore focus on expanding the repertoire of SPOP E3 ligases for PROTAC development, a critical step for advancing this field. Such expansion could help address emerging resistance issues, enhance tissue and cell-type specificity, and significantly improve the therapeutic window.

Importantly, for cancers with SPOP loss-of-function mutations, SPOP ligand-based PROTACs are ineffective. In contrast, Wang *et al*. developed potent small-molecule PROTACs for AR degradation 303. Using a strong AR antagonist and E3 ligase ligands with weak VHL binding affinities, they identified compound 11 (ARD-266), which induced over 95% AR protein degradation in AR+ PCa cell lines (LNCaP, VCaP, 22Rv1) and suppressed *AR*-regulated gene expression 303. This approach shows promise for treating SPOP-mutated PCa. Comparable strategies could be employed to eliminate the cytoplasmic oncogenic activities of SPOP as a potential treatment for RCC.

## 7. Conclusion

Emerging insights into the diverse substrates of SPOP across various cancer types reveal a complex network of interactions that can either promote or inhibit tumorigenesis. Understanding these molecular interactions is crucial for the development of targeted therapies that can modulate SPOP activity and its downstream effects. Future trends in cancer therapy are likely to focus on the creation of small molecule inhibitors or activators of SPOP, tailored to specific cancer types and their underlying genetic aberrations. Additionally, integrating SPOP-targeted therapies with current treatment modalities, such as immunotherapy and precision medicine, holds significant promise for enhancing therapeutic efficacy and overcoming resistance mechanisms. Continued research into the SPOP interactome and its regulatory pathways will undoubtedly broaden our therapeutic arsenal, offering new hope for patients with SPOP-related malignancies.

In summary, the multifaceted role of SPOP in cancer biology presents both challenges and opportunities. By deepening our understanding of its diverse substrates and their contributions to carcinogenesis, we can pave the way for innovative and more effective cancer therapies in the future.

## Supplementary Material

Supplementary figures and tables.

## Figures and Tables

**Table 1:** Regulators that enhance SPOP expression.

Regulators	Regulation mechanism	Tumor types	References
C/EBPα	C/EBPα binds to the promoter of the SPOP gene to enhance the expression of SPOP mRNA	NSCLC	98
LncRNA ADAMTS9-AS2	The underlying molecular mechanism remains unclear	GC	105
CDK1	Preventing SPOP degradation mediated by CDK1	PCa	106
Dzip1	Dzip1 regulates Gli turnover by preventing proteasome-dependent degradation of SPOP	Non cancer (Embryo)	107

Abbreviations: Dzip1: DAZ-interacting protein 1; GC: gastric cancer; NSCLC: non-small cell lung cancer; PCa: prostate cancer.

**Table 2:** Regulators that reduce SPOP expression.

Regulators	Regulation mechanism	Tumor types	References
miRNAs from exosome (miR-520/372/373; miRNA-543; microRNA-17-5p)	Targeting the 3' UTR of SPOP transcripts diminishes SPOP mRNA levels, thereby inhibiting SPOP protein expression	RCC; GC; CRC	99-101
SMAD3	Recognizing SBEs in the SPOP promoter, SMAD3 directly binds to it and represses SPOP transcription	PCa	108
Promoter hypermethylation	Hypermethylation of specific CpG sites within the SPOP promoter region has been observed	CRC; NSCLC	80,98
LIMK2	LIMK2 promotes SPOP degradation through direct phosphorylation	CRPC	102
Aurora A	AURKA directly phosphorylates SPOP, leading to its ubiquitylation	CRPC	103
Snail	Snail promotes SPOP ubiquitination and degradation through its BTB domain	PCa	104

Abbreviations: BTB: bric-a-brac/tramtrack/broad complex; CRC: colorectal cancer; CRPC: castration-resistant prostate cancer; GC: gastric cancer; NSCLC: non-small cell lung cancer; PCa: prostate cancer; RCC: renal cell carcinoma; SBEs: SMAD-binding elements.

**Table 3:** Regulators that influence SPOP's function.

Regulators	Regulation mechanism	Tumor types	References
HIFs	Under hypoxic conditions, HIFs promote the cytoplasmic accumulation of SPOP and influence the degradation of its substrates	RCC	31
ATM	SPOP is phosphorylated at Ser119 by the ATM kinase (serine/threonine), modulating its interaction with substrates in response to DNA damage	PCa	52-54
GRK2	Phosphorylation of the serine residue at codon 222 (SPOP^S222^) disrupts SPOP dimerization, triggering SPOP self-ubiquitylation and degradation	Breast cancer	75
SPOPL	SPOP and SPOPL (SPOP-like) form a molecular rheostat that fine-tunes E3 ubiquitin ligase activity by modulating the oligomeric state of the E3 complex	/	109
G3BP1	G3BP1 competes with SPOP substrates for binding to the MATH domain, inhibiting SPOP's ubiquitination activity	PCa	110

Abbreviations: ATM: Ataxia-telangiectasia mutated; HIF: Hypoxia-inducible factor; PCa: prostate cancer; RCC: renal cell carcinoma; SBEs: SMAD-binding elements; SPOP: Speckle-type POZ protein.

**Table 4:** Human SPOP substrates across different cancer types.

Substrates	Degron sequences in human	Cellular functions	Cancer types	References
				
AR	203-EGSSS-207aa/645-ASSTT-649aa	PCa: AR signaling activation; KC: Sunitinib resistance	PCa KC	38,94,111
ATF2	192-PTSST-196 aa/ 318-ATSTT-322 aa	Cell proliferation, migration and invasion	PCa	39
CyclinE1	306-HFSSS-310 aa	Proliferation, migration, and tumor formation	PCa	40
c-Myc	185-VCSTS-189aa/ 261-PTTSS-265aa	PCa: Cell proliferation; Breast cancer: Epithelial-mesenchymal transition	PCa Breast cancer	41,42
CDCA5	121-AESSS-125aa	Cell survival and proliferation	PCa	30
DEK	285-ADSST-299aa	Cell invasion	PCa	112
EglN2	17-PGSSS-21aa/67-ATSTT-71aa	Facilitated PCa growth	PCa	43
ERG	42-ASSSS-46aa	Cell migration and invasion	PCa	44
SRC3	99-DVSST-103	PCa: Cell migration and invasion; Breast cancer: Tumor growth and proliferation	PCa Breast cancer	69
Gli3	1177-VQSSS-1181aa	AR signaling activation	PCa	45
ITCH	281-DGSST-285aa	Metastasis	PCa	46
PrLZ	30-42aa	Promoting cell growth, chemotherapy resistance, cell migration and invasion	PCa	47
BRD2/3/4	BRD2 (287-291aa), BRD3 (250-254aa), BRD4 (296-300aa): ADTTT	PCa: Decreasing drug resistance; Endometrial cancer: Increasing cell resistance to BET inhibitors	PCa Endometrial cancer	35,36
Cdc20	61-GKSSS-65aa	Drug resistance	PCa	48
TRIM24	151-VPSST-155aa/594-DCSST-598aa	AR signaling activation	PCa	112
Caprin1	35-VSSTS-39aa	Docetaxel resistance	PCa	49
SENP7	201-LSSSS-205aa/393-AGSTT-397aa	Inhibiting senescence	PCa HCC	60
PD-L1	285-HLEET-289aa	Promoting immune escape and decrease chemotherapy sensitivity	PCa Ovarian cancer	61,73
HIPK2	97-ASSTS-101aa/863- ASSTT-867aa	DNA damage	PCa	52
53BP1	1641-ASSSS-1645aa	Genomic instability	PCa	53
MCM3	123-FPSSS-127aa	DNA damage repair	PCa	54
Geminin	200-VSSST-204aa	Genomic instability	PCa	55
BMI1	288-HISST-292aa	X-chromosome inactivation	PCa	56
MacroH2A	285-ADSST-289aa	X-chromosome inactivation	PCa	56
Pdx1	/	β cell mass and function	PCa	57
FASN	160-ACSSS-164aa/1715-LDSTS-1719aa/2251-EGSTT-2255aa	Lipid accumulation	PCa	58
Nanog	66-PDSST-70aa	PCa: Stem cell traits; Pancreatic cancer: Promoting growth and metastasis	PCa Pancreatic cancer	63,82
DDIT3	96-VTSTS-100aa	Apoptotic execution pathways triggered by endoplasmic reticulum stress	PCa	65
INF2	1144-ADSTS-1148aa	Mitochondrial fission	PCa	113
17βHSD4	315-RATST-319aa	Androgen synthesis	PCa	59
GLP	645-ADTTS-649aa/667-ADTTT-671aa	DNA methylation	PCa	66
PDK1	VSSSS	Activating the AKT kinase	PCa	67
SQSTM1	272-PESSS-276aa	Autophagy and Nrf2 activation	PCa	68
LRP5	1481-ASSSS-1485aa	Transcriptional inhibition and inhibit T cell activity	PCa	62
ELK3	129-LRSTS-133aa/101-LPSTS-105aa	Docetaxel resistance	PCa	50
PR	98-GSSSS-102aa	Cell growth and invasion	Breast cancer	70
BRMS1	189-GSSRS-193aa	Suppressing metastasis	Breast cancer	76
ASCT2	349-GTSSS-353aa	Glutamine uptake and metabolism	Breast cancer	75
TWIST1	4-DVSSS-9aa	Cell migration and invasion	Breast cancer	114
ERα	461-FLSST-465aa/571-AGSTS-575aa	Cell proliferation, migration, and invasion	Endometrial cancer	115
IRF1	208-PDSTS-212aa	The inducible expression of PD-L1	Endometrial cancer	71
BRAF	120-VTSSS-124 aa	Activation of the MAPK/ERK pathway	Endometrial cancer	74
ZBTB3	196-LSSTS-200 aa, 272-PSSST-276 aa	Cell proliferation, migration, and invasion	Endometrial cancer	116
DRAK1	/	Inhibiting growth of paclitaxel-resistant cervical cancer cells	Cervical cancer	77
CXCL16	/	Promoting immune tolerance	Cervical cancer	72
Nogo-B	9-LVSSS-13aa/113-PVSST-117aa/169-173aaPPSTP/181-GSSGS-185aa	Promoting carcinogenesis	HCC	78
HMGCS1	143-IESSS-147aa	Activating YAP1 to promote tumor growth	HCC	84
IRF2BP2	447-VHSTT-451aa	Inhibiting cell proliferation and metastasis	HCC	37
BCLAF1	137- PRSSS-141 aa	Stabilizing PD‑L1 and promote the development and immune escape	HCC	86
Gli2	371-PSSTS-375aa/1362-VSSST-1366aa	CRC: Resisting cell death; GC: Promoting cell viability, migration, proliferation, and attenuated apoptosis	CRC GC	80,117
HDAC6	7-DSTTT-11aa /843-GPSSS-847aa	Tumorigenesis and metastasis	CRC	81
ILF3	360-PPSTT-364aa	Increasing *SGOC* genes expression and facilitating tumor growth	CRC	85
TIAM1	210-QHSST-214aa	Promoting the proliferation, migration and invasion	GC	83
FADD	201-DASTS-205aa	Promoting NF-κB activity	Lung cancer	87
SIRT2	49-GISTS-53aa	Promoting cell growth	Lung cancer	88
CHAF1A	281-PSSTS-285aa	Enhancing aggressiveness, including cell proliferation, migration	DLBCL	89
MyD88	14-VSSTS-18 aa	NF-κB signaling activation	DLBCL	90
DHX9	341-PWTSS-345aa	Promoting migration and invasion	Choriocarcinoma	91
EWS-FLI1	462-VTSSS-466aa	Promoting growth	Ewing sarcoma	92
STAT3	512-FSSTT-516aa	Elevated chemokine CCL2 secretion	Bladder cancer	93
Daxx	608-VSSTS-612aa/680-ADSST-684aa	Apoptosis	KC	31
DUSP7	191- VDSSS-195aa	Inhibit cell proliferation	KC	31
Gli2	371-PSSTS-375aa/1362-VSSST-1366aa	Cell proliferation, anti-apoptosis	KC	31
PTEN	359-ASSST-363aa	Inhibit cell proliferation	KC	31
SETD2	1238-SSS-1240aa/1268-STT-1270aa/1373-SSNS-1376aa	H3K36 trimethyltransferase	KC	95
LATS1	332-MQSSS-336aa/434-PQSSS-438aa	Inhibit cell invasion	KC	96

Abbreviations: 53BP1: p53 binding protein 1; AR: Androgen receptor; ASCT2: Alanine serine cysteine transporter 2; ATF2: Activating transcription factor 2; BCLAF1: B cell lymphoma-2-associated transcription factor 1; BMI1: B-lymphoma Mo-MLV insertion region 1; BRAF: B-Raf proto-oncogene; BRD2/3/4: Bromodomain containing proteins 2/3/4; BRMS1: Breast cancer metastasis suppressor 1; Cdc20: Cell division cycle 20; CDCA5: Cell division cycle associated 5; CHAF1A: Chromatin assembly factor 1 subunit A; CXCL16: C-X-C motif chemokine ligand 16; DDIT3: DNA damage inducible transcript 3; DLBCL: Diffuse large B-cell lymphoma; DRAK1:Death-associated protein kinase-related apoptosis-inducing kinase 1; EglN2: Egl-9 family hypoxia inducible factor 2; Erα: Estrogen receptor α; ERG: ETS-related gene; FADD: FAS-associated death structural domain; GC: Gastric cancer; HCC: Hepatocellular carcinoma; HDAC: Histone deacetylases; HIPK2: Homeodomain interacting protein kinase 2; HMGCS1: 3-hydroxy-3-methylglutaryl-CoA synthase 1; INF2: Inverted formin 2; IRF2BP2: Interferon regulatory factor 2-binding protein 2; IRF1: Interferon regulatory factor 1; KC: Kidney cancer; LRP5: Low-density lipoprotein receptor-related protein 5; MCM3: Minichromosome maintenance complex component 3; NF-κB: Nuclear factor kappa-light-chain-enhancer of activated B cells; PCa: Prostate cancer; Pdx1: Pancreatic duodenal homeobox 1; PDK1: 3-phosphoinositide-dependent kinase 1; PD-L1: Programmed death-ligand 1; PR: Progesterone receptor; PrLZ: Prostate leucine zipper; SENP7: Sentrin/SUMO-specific protease 7; SGOC: Serine-glycine-one-carbon; STAT3: Signal transducers and transcriptional activators 3; TIAM1: T lymphoma invasion and metastasis 1; TRIM24: Tripartite motif containing 24; TWIST1: Twist family BHLH transcription factor 1; ZBTB3: Zinc finger and BTB domain-containing protein 3.

## References

[1] Ciechanover A (2015). The unravelling of the ubiquitin system. Nat Rev Mol Cell Biol.

[2] Chen H-Y, Chen R-H (2016). Cullin 3 Ubiquitin Ligases in Cancer Biology: Functions and Therapeutic Implications. Front Oncol.

[3] Wang Z, Liu P, Inuzuka H, Wei W (2014). Roles of F-box proteins in cancer. Nat Rev Cancer.

[4] Skaar JR, Pagan JK, Pagano M (2014). SCF ubiquitin ligase-targeted therapies. Nat Rev Drug Discov.

[5] Hershko A (1983). Ubiquitin: Roles in protein modification and breakdown. Cell.

[6] Scheffner M, Nuber U, Huibregtse JM (1995). Protein ubiquitination involving an E1-E2-E3 enzyme ubiquitin thioester cascade. Nature.

[7] Hochstrasser M (1996). Ubiquitin-dependent protein degradation. Annu Rev Genet.

[8] Hershko A, Ciechanover A (1998). The ubiquitin system. Annu Rev Biochem.

[9] Li W, Bengtson MH, Ulbrich A (2008). Genome-Wide and Functional Annotation of Human E3 Ubiquitin Ligases Identifies MULAN, a Mitochondrial E3 that Regulates the Organelle's Dynamics and Signaling. Ploegh H, Ed. PLoS ONE.

[10] Schulman BA, Wade Harper J (2009). Ubiquitin-like protein activation by E1 enzymes: the apex for downstream signalling pathways. Nat Rev Mol Cell Biol.

[11] Berndsen CE, Wolberger C (2014). New insights into ubiquitin E3 ligase mechanism. Nat Struct Mol Biol.

[12] Deshaies RJ, Joazeiro CAP (2009). RING domain E3 ubiquitin ligases. Annu Rev Biochem.

[13] Budhidarmo R, Nakatani Y, Day CL (2012). RINGs hold the key to ubiquitin transfer. Trends Biochem Sci.

[14] Wang Z, Song Y, Ye M, Dai X, Zhu X, Wei W (2020). The diverse roles of SPOP in prostate cancer and kidney cancer. Nat Rev Urol.

[15] Metzger MB, Hristova VA, Weissman AM (2012). HECT and RING finger families of E3 ubiquitin ligases at a glance. J Cell Sci.

[16] Freemont PS, Hanson IM, Trowsdale J (1991). A novel cysteine-rich sequence motif. Cell.

[17] Rotin D, Kumar S (2009). Physiological functions of the HECT family of ubiquitin ligases. Nat Rev Mol Cell Biol.

[18] Huang L, Kinnucan E, Wang G (1999). Structure of an E6AP-UbcH7 complex: insights into ubiquitination by the E2-E3 enzyme cascade. Science.

[19] van der Reijden BA, Erpelinck-Verschueren CA, Löwenberg B, Jansen JH (1999). TRIADs: a new class of proteins with a novel cysteine-rich signature. Protein Sci.

[20] Wenzel DM, Lissounov A, Brzovic PS, Klevit RE (2011). UBCH7 reactivity profile reveals parkin and HHARI to be RING/HECT hybrids. Nature.

[21] Ishikawa K, Ishihara A, Moriya H (2020). Exploring the Complexity of Protein-Level Dosage Compensation that Fine-Tunes Stoichiometry of Multiprotein Complexes. PLoS Genet.

[22] Jang H-S, Lee Y, Kim Y, Huh W-K (2024). The ubiquitin-proteasome system degrades fatty acid synthase under nitrogen starvation when autophagy is dysfunctional in Saccharomyces cerevisiae. Biochem Biophys Res Commun.

[23] Petroski MD, Deshaies RJ (2005). Function and regulation of cullin-RING ubiquitin ligases. Nat Rev Mol Cell Biol.

[24] Song Y, Xu Y, Pan C, Yan L, Wang Z, Zhu X (2020). The emerging role of SPOP protein in tumorigenesis and cancer therapy. Mol Cancer.

[25] Li X-M, Wu H-L, Xia Q-D (2022). Novel insights into the SPOP E3 ubiquitin ligase: From the regulation of molecular mechanisms to tumorigenesis. Biomedicine & Pharmacotherapy.

[26] Ruel L, Thérond PP (2009). Variations in Hedgehog signaling: divergence and perpetuation in Sufu regulation of Gli. Genes Dev.

[27] Cai H, Liu A (2016). Spop promotes skeletal development and homeostasis by positively regulating Ihh signaling. Proc Natl Acad Sci U S A.

[28] Yang X, Zhu Q (2022). SPOP in Cancer: Phenomena, Mechanisms and Its Role in Therapeutic Implications. Genes (Basel).

[29] Nagai Y, Kojima T, Muro Y (1997). Identification of a novel nuclear speckle-type protein, SPOP. FEBS Lett.

[30] Luo Z, Wang J, Zhu Y (2021). SPOP promotes CDCA5 degradation to regulate prostate cancer progression via the AKT pathway. Neoplasia.

[31] Li G, Ci W, Karmakar S (2014). SPOP promotes tumorigenesis by acting as a key regulatory hub in kidney cancer. Cancer Cell.

[32] Zhang H, Kong L, Li J (2025). SPOP mutations increase PARP inhibitor sensitivity via CK2/PIAS1/SPOP axis in prostate cancer. JCI Insight.

[33] Zhuang M, Calabrese MF, Liu J (2009). Structures of SPOP-substrate complexes: insights into molecular architectures of BTB-Cul3 ubiquitin ligases. Mol Cell.

[34] Zhang H, Jin X, Huang H (2023). Deregulation of SPOP in Cancer. Cancer Res.

[35] Zhang P, Wang D, Zhao Y (2017). Intrinsic BET inhibitor resistance in SPOP-mutated prostate cancer is mediated by BET protein stabilization and AKT-mTORC1 activation. Nat Med.

[36] Janouskova H, El Tekle G, Bellini E (2017). Opposing effects of cancer-type-specific SPOP mutants on BET protein degradation and sensitivity to BET inhibitors. Nat Med.

[37] Deng Y, Ding W, Ma K (2024). SPOP point mutations regulate substrate preference and affect its function. Cell Death Dis.

[38] Geng C, Rajapakshe K, Shah SS (2014). Androgen receptor is the key transcriptional mediator of the tumor suppressor SPOP in prostate cancer. Cancer Res.

[39] Ma J, Chang K, Peng J (2018). SPOP promotes ATF2 ubiquitination and degradation to suppress prostate cancer progression. J Exp Clin Cancer Res.

[40] Ju L-G, Zhu Y, Long Q-Y (2019). SPOP suppresses prostate cancer through regulation of CYCLIN E1 stability. Cell Death Differ.

[41] Geng C, Kaochar S, Li M (2017). SPOP regulates prostate epithelial cell proliferation and promotes ubiquitination and turnover of c-MYC oncoprotein. Oncogene.

[42] Luo L, Tang H, Ling L (2018). LINC01638 lncRNA activates MTDH-Twist1 signaling by preventing SPOP-mediated c-Myc degradation in triple-negative breast cancer. Oncogene.

[43] Zhang L, Peng S, Dai X (2017). Tumor suppressor SPOP ubiquitinates and degrades EglN2 to compromise growth of prostate cancer cells. Cancer Lett.

[44] Gan W, Dai X, Lunardi A (2015). SPOP Promotes Ubiquitination and Degradation of the ERG Oncoprotein to Suppress Prostate Cancer Progression. Mol Cell.

[45] Burleson M, Deng JJ, Qin T (2022). GLI3 Is Stabilized by SPOP Mutations and Promotes Castration Resistance via Functional Cooperation with Androgen Receptor in Prostate Cancer. Mol Cancer Res.

[46] Ma J, Cai M, Mo Y (2021). The SPOP-ITCH Signaling Axis Protects Against Prostate Cancer Metastasis. Front Oncol.

[47] Fan Y, Hou T, Dan W (2022). ERK1/2 inhibits Cullin 3/SPOP-mediated PrLZ ubiquitination and degradation to modulate prostate cancer progression. Cell Death Differ.

[48] Wu F, Dai X, Gan W (2017). Prostate cancer-associated mutation in SPOP impairs its ability to target Cdc20 for poly-ubiquitination and degradation. Cancer Lett.

[49] Shi Q, Zhu Y, Ma J (2019). Prostate Cancer-associated SPOP mutations enhance cancer cell survival and docetaxel resistance by upregulating Caprin1-dependent stress granule assembly. Mol Cancer.

[50] Lee C-J, Lee H, Kim SR (2024). ELK3 destabilization by speckle-type POZ protein suppresses prostate cancer progression and docetaxel resistance. Cell Death Dis.

[51] Groner AC, Cato L, de Tribolet-Hardy J (2016). TRIM24 Is an Oncogenic Transcriptional Activator in Prostate Cancer. Cancer Cell.

[52] Jin X, Qing S, Li Q (2021). Prostate cancer-associated SPOP mutations lead to genomic instability through disruption of the SPOP-HIPK2 axis. Nucleic Acids Res.

[53] Wang D, Ma J, Botuyan MV (2021). ATM-phosphorylated SPOP contributes to 53BP1 exclusion from chromatin during DNA replication. Sci Adv.

[54] Xiao M, Fried JS, Ma J (2021). A disease-relevant mutation of SPOP highlights functional significance of ATM-mediated DNA damage response. Signal Transduct Target Ther.

[55] Ma J, Shi Q, Cui G (2021). SPOP mutation induces replication over-firing by impairing Geminin ubiquitination and triggers replication catastrophe upon ATR inhibition. Nat Commun.

[56] Hernández-Muñoz I, Lund AH, van der Stoop P (2005). Stable X chromosome inactivation involves the PRC1 Polycomb complex and requires histone MACROH2A1 and the CULLIN3/SPOP ubiquitin E3 ligase. Proc Natl Acad Sci U S A.

[57] Claiborn KC, Sachdeva MM, Cannon CE, Groff DN, Singer JD, Stoffers DA (2010). Pcif1 modulates Pdx1 protein stability and pancreatic β cell function and survival in mice. J Clin Invest.

[58] Gang X, Xuan L, Zhao X (2019). Speckle-type POZ protein suppresses lipid accumulation and prostate cancer growth by stabilizing fatty acid synthase. Prostate.

[59] Shi L, Yan Y, He Y (2021). Mutated SPOP E3 Ligase Promotes 17βHSD4 Protein Degradation to Drive Androgenesis and Prostate Cancer Progression. Cancer Res.

[60] Zhu H, Ren S, Bitler BG (2015). SPOP E3 Ubiquitin Ligase Adaptor Promotes Cellular Senescence by Degrading the SENP7 deSUMOylase. Cell Rep.

[61] Zhang J, Bu X, Wang H (2018). Cyclin D-CDK4 kinase destabilizes PD-L1 via cullin 3-SPOP to control cancer immune surveillance. Nature.

[62] Gan S, Qu F, Zhang X (2024). LRP5 competes for SPOP binding to enhance tumorigenesis mediated by Daxx and PD-L1 in prostate cancer. Exp Cell Res.

[63] Zhang J, Chen M, Zhu Y (2019). SPOP Promotes Nanog Destruction to Suppress Stem Cell Traits and Prostate Cancer Progression. Dev Cell.

[64] Wang X, Jin J, Wan F (2019). AMPK Promotes SPOP-Mediated NANOG Degradation to Regulate Prostate Cancer Cell Stemness. Dev Cell.

[65] Zhang P, Gao K, Tang Y (2014). Destruction of DDIT3/CHOP protein by wild-type SPOP but not prostate cancer-associated mutants. Hum Mutat.

[66] Zhang J, Gao K, Xie H (2021). SPOP mutation induces DNA methylation via stabilizing GLP/G9a. Nat Commun.

[67] Jiang Q, Zheng N, Bu L (2021). SPOP-mediated ubiquitination and degradation of PDK1 suppresses AKT kinase activity and oncogenic functions. Mol Cancer.

[68] Shi Q, Jin X, Zhang P (2022). SPOP mutations promote p62/SQSTM1-dependent autophagy and Nrf2 activation in prostate cancer. Cell Death Differ.

[69] Li C, Ao J, Fu J (2011). Tumor-suppressor role for the SPOP ubiquitin ligase in signal-dependent proteolysis of the oncogenic co-activator SRC-3/AIB1. Oncogene.

[70] Gao K, Jin X, Tang Y (2015). Tumor suppressor SPOP mediates the proteasomal degradation of progesterone receptors (PRs) in breast cancer cells. Am J Cancer Res.

[71] Gao K, Shi Q, Gu Y (2023). SPOP mutations promote tumor immune escape in endometrial cancer via the IRF1-PD-L1 axis. Cell Death Differ.

[72] Wu J, Wu Y, Guo Q (2022). SPOP promotes cervical cancer progression by inducing the movement of PD-1 away from PD-L1 in spatial localization. J Transl Med.

[73] Dong M, Qian M, Ruan Z (2022). CUL3/SPOP complex prevents immune escape and enhances chemotherapy sensitivity of ovarian cancer cells through degradation of PD-L1 protein. J Immunother Cancer.

[74] Feng K, Shi Q, Jiao D (2022). SPOP inhibits BRAF-dependent tumorigenesis through promoting non-degradative ubiquitination of BRAF. Cell Biosci.

[75] Zhou Q, Lin W, Wang C (2022). Neddylation inhibition induces glutamine uptake and metabolism by targeting CRL3SPOP E3 ligase in cancer cells. Nat Commun.

[76] Kim B, Nam HJ, Pyo KE (2011). Breast cancer metastasis suppressor 1 (BRMS1) is destabilized by the Cul3-SPOP E3 ubiquitin ligase complex. Biochem Biophys Res Commun.

[77] Pang K, Lee J, Kim J (2022). Degradation of DRAK1 by CUL3/SPOP E3 Ubiquitin ligase promotes tumor growth of paclitaxel-resistant cervical cancer cells. Cell Death Dis.

[78] Zhou P, Chang W-Y, Gong D-A (2023). O-GlcNAcylation of SPOP promotes carcinogenesis in hepatocellular carcinoma. Oncogene.

[79] Ji P, Liang S, Li P (2018). Speckle-type POZ protein suppresses hepatocellular carcinoma cell migration and invasion via ubiquitin-dependent proteolysis of SUMO1/sentrin specific peptidase 7. Biochem Biophys Res Commun.

[80] Zhi X, Tao J, Zhang L, Tao R, Ma L, Qin J (2016). Silencing speckle-type POZ protein by promoter hypermethylation decreases cell apoptosis through upregulating Hedgehog signaling pathway in colorectal cancer. Cell Death Dis.

[81] Tan Y, Ci Y, Dai X (2017). Cullin 3SPOP ubiquitin E3 ligase promotes the poly-ubiquitination and degradation of HDAC6. Oncotarget.

[82] Tan P, Xu Y, Du Y (2019). SPOP suppresses pancreatic cancer progression by promoting the degradation of NANOG. Cell Death Dis.

[83] Liu F, Zhang T, Sun X (2024). Deficiency in SPOP-mediated ubiquitination and degradation of TIAM1 promotes gastric cancer progression. Biochim Biophys Acta Mol Basis Dis.

[84] Li K, Zhang J, Lyu H (2024). CSN6-SPOP-HMGCS1 Axis Promotes Hepatocellular Carcinoma Progression via YAP1 Activation. Adv Sci (Weinh).

[85] Li K, Wu J-L, Qin B (2020). ILF3 is a substrate of SPOP for regulating serine biosynthesis in colorectal cancer. Cell Res.

[86] Yu Z, Wu X, Zhu J (2024). BCLAF1 binds SPOP to stabilize PD-L1 and promotes the development and immune escape of hepatocellular carcinoma. Cell Mol Life Sci.

[87] Luo J, Chen B, Gao C-X, Xie H-K, Han C-N, Zhou C-C (2018). SPOP promotes FADD degradation and inhibits NF-κB activity in non-small cell lung cancer. Biochem Biophys Res Commun.

[88] Luo J, Bao Y-C, Ji X-X, Chen B, Deng Q-F, Zhou S-W (2017). SPOP promotes SIRT2 degradation and suppresses non-small cell lung cancer cell growth. Biochem Biophys Res Commun.

[89] Yan W, Shi X, Wang H, Liao A, Yang W (2022). Aberrant SPOP-CHAF1A ubiquitination axis triggers tumor autophagy that endows a therapeutical vulnerability in diffuse large B cell lymphoma. J Transl Med.

[90] Jin X, Shi Q, Li Q (2020). CRL3-SPOP ubiquitin ligase complex suppresses the growth of diffuse large B-cell lymphoma by negatively regulating the MyD88/NF-κB signaling. Leukemia.

[91] Yuan D, Chen Y, Yang Z (2020). SPOP attenuates migration and invasion of choriocarcinoma cells by promoting DHX9 degradation. Am J Cancer Res.

[92] Su S, Chen J, Jiang Y (2021). SPOP and OTUD7A Control EWS-FLI1 Protein Stability to Govern Ewing Sarcoma Growth. Adv Sci (Weinh).

[93] Li M, Cui Y, Qi Q (2024). SPOP downregulation promotes bladder cancer progression based on cancer cell-macrophage crosstalk via STAT3/CCL2/IL-6 axis and is regulated by VEZF1. Theranostics.

[94] Adelaiye-Ogala R, Damayanti NP, Orillion AR (2018). Therapeutic Targeting of Sunitinib-Induced AR Phosphorylation in Renal Cell Carcinoma. Cancer Res.

[95] Zhu K, Lei P-J, Ju L-G (2017). SPOP-containing complex regulates SETD2 stability and H3K36me3-coupled alternative splicing. Nucleic Acids Res.

[96] Wang L, Lin M, Chu M (2020). SPOP promotes ubiquitination and degradation of LATS1 to enhance kidney cancer progression. EBioMedicine.

[97] Bouchard JJ, Otero JH, Scott DC (2018). Cancer Mutations of the Tumor Suppressor SPOP Disrupt the Formation of Active, Phase-Separated Compartments. Mol Cell.

[98] Yao S, Chen X, Chen J (2018). Speckle-type POZ protein functions as a tumor suppressor in non-small cell lung cancer due to DNA methylation. Cancer Cell Int.

[99] Ding M, Lu X, Wang C (2018). The E2F1-miR-520/372/373-SPOP Axis Modulates Progression of Renal Carcinoma. Cancer Res.

[100] Xu J, Wang F, Wang X, He Z, Zhu X (2018). miRNA-543 promotes cell migration and invasion by targeting SPOP in gastric cancer. Onco Targets Ther.

[101] Sun W, Cui J, Ge Y (2022). Tumor stem cell-derived exosomal microRNA-17-5p inhibits anti-tumor immunity in colorectal cancer via targeting SPOP and overexpressing PD-L1. Cell Death Discov.

[102] Nikhil K, Haymour HS, Kamra M, Shah K (2021). Phosphorylation-dependent regulation of SPOP by LIMK2 promotes castration-resistant prostate cancer. Br J Cancer.

[103] Nikhil K, Kamra M, Raza A, Haymour HS, Shah K (2020). Molecular Interplay between AURKA and SPOP Dictates CRPC Pathogenesis via Androgen Receptor. Cancers (Basel).

[104] Lv W, Huan M, Yang W (2020). Snail promotes prostate cancer migration by facilitating SPOP ubiquitination and degradation. Biochem Biophys Res Commun.

[105] Wang F, Tang C, Xu D (2020). LncRNA ADAMTS9-AS2 suppresses the proliferation of gastric cancer cells and the tumorigenicity of cancer stem cells through regulating SPOP. J Cell Mol Med.

[106] Tang Z, Pilié PG, Geng C (2021). ATR Inhibition Induces CDK1-SPOP Signaling and Enhances Anti-PD-L1 Cytotoxicity in Prostate Cancer. Clin Cancer Res.

[107] Schwend T, Jin Z, Jiang K, Mitchell BJ, Jia J, Yang J (2013). Stabilization of speckle-type POZ protein (Spop) by Daz interacting protein 1 (Dzip1) is essential for Gli turnover and the proper output of Hedgehog signaling. J Biol Chem.

[108] Jiao C, Meng T, Zhou C (2020). TGF-β signaling regulates SPOP expression and promotes prostate cancer cell stemness. Aging (Albany NY).

[109] Errington WJ, Khan MQ, Bueler SA, Rubinstein JL, Chakrabartty A, Privé GG (2012). Adaptor protein self-assembly drives the control of a cullin-RING ubiquitin ligase. Structure.

[110] Mukhopadhyay C, Yang C, Xu L (2021). G3BP1 inhibits Cul3SPOP to amplify AR signaling and promote prostate cancer. Nat Commun.

[111] An J, Wang C, Deng Y, Yu L, Huang H (2014). Destruction of full-length androgen receptor by wild-type SPOP, but not prostate-cancer-associated mutants. Cell Rep.

[112] Theurillat J-PP, Udeshi ND, Errington WJ (2014). Prostate cancer. Ubiquitylome analysis identifies dysregulation of effector substrates in SPOP-mutant prostate cancer. Science.

[113] Jin X, Wang J, Gao K (2017). Dysregulation of INF2-mediated mitochondrial fission in SPOP-mutated prostate cancer. PLoS Genet.

[114] Wei C, Liu Y, Liu X (2022). The speckle-type POZ protein (SPOP) inhibits breast cancer malignancy by destabilizing TWIST1. Cell Death Discov.

[115] Zhang N, Sun P, Xu Y (2021). The GPER1/SPOP axis mediates ubiquitination-dependent degradation of ERα to inhibit the growth of breast cancer induced by oestrogen. Cancer Lett.

[116] Jin X, Wang J, Li Q (2019). SPOP targets oncogenic protein ZBTB3 for destruction to suppress endometrial cancer. Am J Cancer Res.

[117] Zeng C, Wang Y, Lu Q (2014). SPOP suppresses tumorigenesis by regulating Hedgehog/Gli2 signaling pathway in gastric cancer. J Exp Clin Cancer Res.

[118] García-Flores M, Casanova-Salas I, Rubio-Briones J (2014). Clinico-pathological significance of the molecular alterations of the SPOP gene in prostate cancer. Eur J Cancer.

[119] Kan Z, Jaiswal BS, Stinson J (2010). Diverse somatic mutation patterns and pathway alterations in human cancers. Nature.

[120] Broomfield J, Kalofonou M, Gulli C (2025). Handheld ISFET Lab-on-Chip detection of YAP1 nucleic acid and AR-FL and AR-V7 mRNA from liquid biopsies for prostate cancer prognosis. Biosens Bioelectron.

[121] Lopez-Bergami P, Lau E, Ronai Z (2010). Emerging roles of ATF2 and the dynamic AP1 network in cancer. Nat Rev Cancer.

[122] Ricote M, García-Tuñón I, Bethencourt F (2006). The p38 transduction pathway in prostatic neoplasia. J Pathol.

[123] Híveš M, Jurečeková J, Kliment J (2022). Role of Genetic Variations in, and in Prostate Cancer. Cancer Genomics Proteomics.

[124] Liao Y, Wu N, Wang K (2020). OTUB1 Promotes Progression and Proliferation of Prostate Cancer via Deubiquitinating and Stabling Cyclin E1. Front Cell Dev Biol.

[125] Gurel B, Iwata T, Koh CM (2008). Nuclear MYC protein overexpression is an early alteration in human prostate carcinogenesis. Mod Pathol.

[126] Sena LA, Kumar R, Sanin DE (2022). Prostate cancer androgen receptor activity dictates efficacy of bipolar androgen therapy through MYC. J Clin Invest.

[127] Vatapalli R, Sagar V, Rodriguez Y (2020). Histone methyltransferase DOT1L coordinates AR and MYC stability in prostate cancer. Nat Commun.

[128] Zhao X, Ha M, Zhou L, Wang Y, Li P (2025). Berberine diminishes the malignant progression of non-small cell lung cancer cells by targeting CDCA5 and CCNA2. J Nat Med.

[129] Ji J, Shen T, Li Y, Liu Y, Shang Z, Niu Y (2021). CDCA5 promotes the progression of prostate cancer by affecting the ERK signalling pathway. Oncol Rep.

[130] Lin D, Dong X, Wang K (2015). Identification of DEK as a potential therapeutic target for neuroendocrine prostate cancer. Oncotarget.

[131] Zheng X, Zhai B, Koivunen P (2014). Prolyl hydroxylation by EglN2 destabilizes FOXO3a by blocking its interaction with the USP9x deubiquitinase. Genes Dev.

[132] Zhang Q, Gu J, Li L (2009). Control of cyclin D1 and breast tumorigenesis by the EglN2 prolyl hydroxylase. Cancer Cell.

[133] Tomlins SA, Rhodes DR, Perner S (2005). Recurrent fusion of TMPRSS2 and ETS transcription factor genes in prostate cancer. Science.

[134] Perner S, Mosquera J-M, Demichelis F (2007). TMPRSS2-ERG fusion prostate cancer: an early molecular event associated with invasion. Am J Surg Pathol.

[135] An J, Ren S, Murphy SJ (2015). Truncated ERG Oncoproteins from TMPRSS2-ERG Fusions Are Resistant to SPOP-Mediated Proteasome Degradation. Mol Cell.

[136] Barbieri CE, Baca SC, Lawrence MS (2012). Exome sequencing identifies recurrent SPOP, FOXA1 and MED12 mutations in prostate cancer. Nat Genet.

[137] Blattner M, Lee DJ, O'Reilly C (2014). SPOP mutations in prostate cancer across demographically diverse patient cohorts. Neoplasia.

[138] Shoag J, Liu D, Blattner M (2018). SPOP mutation drives prostate neoplasia without stabilizing oncogenic transcription factor ERG. J Clin Invest.

[139] Gnanapragasam VJ, Leung HY, Pulimood AS, Neal DE, Robson CN (2001). Expression of RAC 3, a steroid hormone receptor co-activator in prostate cancer. Br J Cancer.

[140] Xu J, Wu R-C, O'Malley BW (2009). Normal and cancer-related functions of the p160 steroid receptor co-activator (SRC) family. Nat Rev Cancer.

[141] Zhou XE, Suino-Powell KM, Li J (2010). Identification of SRC3/AIB1 as a preferred coactivator for hormone-activated androgen receptor. J Biol Chem.

[142] Zhou H-J, Yan J, Luo W (2005). SRC-3 is required for prostate cancer cell proliferation and survival. Cancer Res.

[143] Geng C, He B, Xu L (2013). Prostate cancer-associated mutations in speckle-type POZ protein (SPOP) regulate steroid receptor coactivator 3 protein turnover. Proc Natl Acad Sci U S A.

[144] Briscoe J, Thérond PP (2013). The mechanisms of Hedgehog signalling and its roles in development and disease. Nat Rev Mol Cell Biol.

[145] Berman DM, Karhadkar SS, Maitra A (2003). Widespread requirement for Hedgehog ligand stimulation in growth of digestive tract tumours. Nature.

[146] Stecca B, Mas C, Ruiz i Altaba A (2005). Interference with HH-GLI signaling inhibits prostate cancer. Trends Mol Med.

[147] Karhadkar SS, Bova GS, Abdallah N (2004). Hedgehog signalling in prostate regeneration, neoplasia and metastasis. Nature.

[148] Sanchez P, Hernández AM, Stecca B (2004). Inhibition of prostate cancer proliferation by interference with SONIC HEDGEHOG-GLI1 signaling. Proc Natl Acad Sci U S A.

[149] Cai H, Liu A (2017). Spop regulates Gli3 activity and Shh signaling in dorsoventral patterning of the mouse spinal cord. Dev Biol.

[150] Lee T-L, Shyu Y-C, Hsu T-Y, Shen C-KJ (2008). Itch regulates p45/NF-E2 *in vivo* by Lys63-linked ubiquitination. Biochem Biophys Res Commun.

[151] Byrne JA, Frost S, Chen Y, Bright RK (2014). Tumor protein D52 (TPD52) and cancer-oncogene understudy or understudied oncogene?. Tumour Biol.

[152] Wang R, Xu J, Saramäki O (2004). PrLZ, a novel prostate-specific and androgen-responsive gene of the TPD52 family, amplified in chromosome 8q21.1 and overexpressed in human prostate cancer. Cancer Res.

[153] Li L, Zhang D, Zhang L (2009). PrLZ expression is associated with the progression of prostate cancer LNCaP cells. Mol Carcinog.

[154] Zhang D, He D, Xue Y (2011). PrLZ protects prostate cancer cells from apoptosis induced by androgen deprivation via the activation of Stat3/Bcl-2 pathway. Cancer Res.

[155] Li L, Xie H, Liang L (2013). Increased PrLZ-mediated androgen receptor transactivation promotes prostate cancer growth at castration-resistant stage. Carcinogenesis.

[156] Zeng J, Liu W, Fan Y-Z, He D-L, Li L (2018). PrLZ increases prostate cancer docetaxel resistance by inhibiting LKB1/AMPK-mediated autophagy. Theranostics.

[157] Belkina AC, Denis GV (2012). BET domain co-regulators in obesity, inflammation and cancer. Nat Rev Cancer.

[158] Jeong SM, Bui QT, Kwak M, Lee JY, Lee PC-W (2022). Targeting Cdc20 for cancer therapy. Biochim Biophys Acta Rev Cancer.

[159] Edwards AC, Stalnecker CA, Jean Morales A (2023). TEAD Inhibition Overcomes YAP1/TAZ-Driven Primary and Acquired Resistance to KRASG12C Inhibitors. Cancer Res.

[160] Schuyler SC, Chen H-Y, Chang K-P (2024). Suppressing Anaphase-Promoting Complex/Cyclosome-Cell Division Cycle 20 Activity to Enhance the Effectiveness of Anti-Cancer Drugs That Induce Multipolar Mitotic Spindles. Int J Mol Sci.

[161] Chen OJ, Castellsagué E, Moustafa-Kamal M (2022). Germline Missense Variants in CDC20 Result in Aberrant Mitotic Progression and Familial Cancer. Cancer Res.

[162] Song H, Wu J, Liu W (2023). Key genes involved with prognosis were identified in lung adenocarcinoma by integrated bioinformatics analysis. Heliyon.

[163] Li Q, Wang W (2024). The predictive significance of CDC20 across various molecular subtypes of breast cancer. Asian J Surg.

[164] Zhao S-F, Leng J-F, Xie S-S (2024). Design, synthesis and biological evaluation of CDC20 inhibitors for treatment of triple-negative breast cancer. Eur J Med Chem.

[165] Chang DZ, Ma Y, Ji B (2012). Increased CDC20 expression is associated with pancreatic ductal adenocarcinoma differentiation and progression. J Hematol Oncol.

[166] Gao Y, Wen P, Chen B (2020). Downregulation of CDC20 Increases Radiosensitivity through Mcl-1/p-Chk1-Mediated DNA Damage and Apoptosis in Tumor Cells. Int J Mol Sci.

[167] Li H, Shi Y, Li Y (2024). DNA damage response-related signatures characterize the immune landscape and predict the prognosis of HCC via integrating single-cell and bulk RNA-sequencing. Int Immunopharmacol.

[168] Hemati M, Haghiralsadat F, Jafary F, Moosavizadeh S, Moradi A (2019). Targeting cell cycle protein in gastric cancer with CDC20siRNA and anticancer drugs (doxorubicin and quercetin) co-loaded cationic PEGylated nanoniosomes. Int J Nanomedicine.

[169] Wang J, Xiao Z, Li P (2023). PRMT6-CDC20 facilitates glioblastoma progression via the degradation of CDKN1B. Oncogene.

[170] Yang C, Ge Y, Zang Y (2023). CDC20 promotes radioresistance of prostate cancer by activating Twist1 expression. Apoptosis.

[171] Chen Z-H, Jing Y-J, Yu J-B (2019). ESRP1 Induces Cervical Cancer Cell G1-Phase Arrest Via Regulating Cyclin A2 mRNA Stability. Int J Mol Sci.

[172] Xian F, Yang X, Xu G (2022). Prognostic significance of CDC20 expression in malignancy patients: A meta-analysis. Front Oncol.

[173] Manchado E, Guillamot M, de Cárcer G (2010). Targeting mitotic exit leads to tumor regression *in vivo*: Modulation by Cdk1, Mastl, and the PP2A/B55α,δ phosphatase. Cancer Cell.

[174] Xu C, Chen G, Yu B (2024). TRIM24 Cooperates with Ras Mutation to Drive Glioma Progression through snoRNA Recruitment of PHAX and DNA-PKcs. Adv Sci.

[175] Fong K-W, Zhao JC, Song B, Zheng B, Yu J (2018). TRIM28 protects TRIM24 from SPOP-mediated degradation and promotes prostate cancer progression. Nat Commun.

[176] Panas MD, Ivanov P, Anderson P (2016). Mechanistic insights into mammalian stress granule dynamics. J Cell Biol.

[177] Gong B, Hu H, Chen J (2013). Caprin-1 is a novel microRNA-223 target for regulating the proliferation and invasion of human breast cancer cells. Biomed Pharmacother.

[178] Guo X-M, Zhu F-F, Pan L-W (2020). Caprin-1 promotes HepG2 cell proliferation, invasion and migration and is associated with poor prognosis in patients with liver cancer. Oncol Lett.

[179] Nozaki M, Onishi Y, Kanno N, Ono Y, Fujimura Y (1996). Molecular cloning of Elk-3, a new member of the Ets family expressed during mouse embryogenesis and analysis of its transcriptional repression activity. DNA Cell Biol.

[180] Mao Y, Li W, Hua B (2020). Silencing of ELK3 Induces S-M Phase Arrest and Apoptosis and Upregulates SERPINE1 Expression Reducing Migration in Prostate Cancer Cells. Biomed Res Int.

[181] Blaquiere JA, Verheyen EM (2017). Homeodomain-Interacting Protein Kinases: Diverse and Complex Roles in Development and Disease. Curr Top Dev Biol.

[182] Hofmann TG, Glas C, Bitomsky N (2013). HIPK2: A tumour suppressor that controls DNA damage-induced cell fate and cytokinesis. Bioessays.

[183] Wu Y-Q, Zhang C-S, Xiong J (2023). Low glucose metabolite 3-phosphoglycerate switches PHGDH from serine synthesis to p53 activation to control cell fate. Cell Res.

[184] Akaike Y, Kuwano Y, Nishida K (2015). Homeodomain-interacting protein kinase 2 regulates DNA damage response through interacting with heterochromatin protein 1γ. Oncogene.

[185] Liebl MC, Hofmann TG (2019). Cell Fate Regulation upon DNA Damage: p53 Serine 46 Kinases Pave the Cell Death Road. Bioessays.

[186] Hofmann TG, Möller A, Sirma H (2002). Regulation of p53 activity by its interaction with homeodomain-interacting protein kinase-2. Nat Cell Biol.

[187] D'Orazi G, Cecchinelli B, Bruno T (2002). Homeodomain-interacting protein kinase-2 phosphorylates p53 at Ser 46 and mediates apoptosis. Nat Cell Biol.

[188] Hustedt N, Durocher D (2016). The control of DNA repair by the cell cycle. Nat Cell Biol.

[189] Ma J, Zhou Y, Pan P (2023). TRABID overexpression enables synthetic lethality to PARP inhibitor via prolonging 53BP1 retention at double-strand breaks. Nat Commun.

[190] King A, Reichl PI, Metson JS (2024). Shieldin and CST co-orchestrate DNA polymerase-dependent tailed-end joining reactions independently of 53BP1-governed repair pathway choice. Nat Struct Mol Biol.

[191] McGarry TJ, Kirschner MW (1998). Geminin, an inhibitor of DNA replication, is degraded during mitosis. Cell.

[192] Gonzalez MA, Tachibana KK, Adams DJ (2006). Geminin is essential to prevent endoreduplication and to form pluripotent cells during mammalian development. Genes Dev.

[193] Tada S (2007). Cdt1 and geminin: role during cell cycle progression and DNA damage in higher eukaryotes. Front Biosci.

[194] Wang Y, Chen H, Zhang J (2020). MCM family in gastrointestinal cancer and other malignancies: From functional characterization to clinical implication. Biochim Biophys Acta Rev Cancer.

[195] Forsburg SL (2004). Eukaryotic MCM proteins: beyond replication initiation. Microbiol Mol Biol Rev.

[196] Gou K, Liu J, Feng X, Li H, Yuan Y, Xing C (2018). Expression of Minichromosome Maintenance Proteins (MCM) and Cancer Prognosis: A meta-analysis. J Cancer.

[197] Nikolic A, Maule F, Bobyn A (2023). macroH2A2 antagonizes epigenetic programs of stemness in glioblastoma. Nat Commun.

[198] Jonsson J, Carlsson L, Edlund T, Edlund H (1994). Insulin-promoter-factor 1 is required for pancreas development in mice. Nature.

[199] Ashizawa S, Brunicardi FC, Wang X-P (2004). PDX-1 and the pancreas. Pancreas.

[200] Menendez JA, Lupu R (2017). Fatty acid synthase (FASN) as a therapeutic target in breast cancer. Expert Opin Ther Targets.

[201] Menendez JA, Lupu R (2007). Fatty acid synthase and the lipogenic phenotype in cancer pathogenesis. Nat Rev Cancer.

[202] Baenke F, Peck B, Miess H, Schulze A (2013). Hooked on fat: the role of lipid synthesis in cancer metabolism and tumour development. Dis Model Mech.

[203] Röhrig F, Schulze A (2016). The multifaceted roles of fatty acid synthesis in cancer. Nat Rev Cancer.

[204] Ko H-K, Berk M, Chung Y-M (2018). Loss of an Androgen-Inactivating and Isoform-Specific HSD17B4 Splice Form Enables Emergence of Castration-Resistant Prostate Cancer. Cell Rep.

[205] Wang J-H, Tuohimaa P (2007). Regulation of 17beta-hydroxysteroid dehydrogenase type 2, type 4 and type 5 by calcitriol, LXR agonist and 5alpha-dihydrotestosterone in human prostate cancer cells. J Steroid Biochem Mol Biol.

[206] Bawa-Khalfe T, Lu L-S, Zuo Y (2012). Differential expression of SUMO-specific protease 7 variants regulates epithelial-mesenchymal transition. Proc Natl Acad Sci U S A.

[207] Garvin AJ, Densham RM, Blair-Reid SA (2013). The deSUMOylase SENP7 promotes chromatin relaxation for homologous recombination DNA repair. EMBO Rep.

[208] Cui Y, Yu H, Zheng X (2017). SENP7 Potentiates cGAS Activation by Relieving SUMO-Mediated Inhibition of Cytosolic DNA Sensing. PLoS Pathog.

[209] Barry R, John SW, Liccardi G (2018). SUMO-mediated regulation of NLRP3 modulates inflammasome activity. Nat Commun.

[210] Liu Q, Guan Y, Li S (2024). Programmed death receptor (PD-)1/PD-ligand (L)1 in urological cancers : the 'all-around warrior' in immunotherapy. Mol Cancer.

[211] Lin X, Kang K, Chen P (2024). Regulatory mechanisms of PD-1/PD-L1 in cancers. Mol Cancer.

[212] Bettuzzi S, Davalli P, Davoli S (2009). Genetic inactivation of ApoJ/clusterin: effects on prostate tumourigenesis and metastatic spread. Oncogene.

[213] Cao J, Zhao M, Liu J (2019). RACK1 Promotes Self-Renewal and Chemoresistance of Cancer Stem Cells in Human Hepatocellular Carcinoma through Stabilizing Nanog. Theranostics.

[214] Jauhiainen A, Thomsen C, Strömbom L (2012). Distinct cytoplasmic and nuclear functions of the stress induced protein DDIT3/CHOP/GADD153. PLoS One.

[215] Matsumura T, Nakamura-Ishizu A, Muddineni SSNA (2020). Hematopoietic stem cells acquire survival advantage by loss of RUNX1 methylation identified in familial leukemia. Blood.

[216] Yu X, Li W, Sun S, Li J (2024). DDIT3 is associated with breast cancer prognosis and immune microenvironment: an integrative bioinformatic and immunohistochemical analysis. J Cancer.

[217] Yu Q, Zhao J, Yang A, Li X (2024). MLLT6/ATF2 Axis Restrains Breast Cancer Progression by Driving DDIT3/4 Expression. Mol Cancer Res.

[218] Jung AR, Shin S, Kim MY (2024). Integrated Bioinformatics Analysis Identified ASNS and DDIT3 as the Therapeutic Target in Castrate-Resistant Prostate Cancer. Int J Mol Sci.

[219] Chhabra ES, Higgs HN (2006). INF2 Is a WASP homology 2 motif-containing formin that severs actin filaments and accelerates both polymerization and depolymerization. J Biol Chem.

[220] Tachibana M, Ueda J, Fukuda M (2005). Histone methyltransferases G9a and GLP form heteromeric complexes and are both crucial for methylation of euchromatin at H3-K9. Genes Dev.

[221] Tachibana M, Sugimoto K, Fukushima T, Shinkai Y (2001). Set domain-containing protein, G9a, is a novel lysine-preferring mammalian histone methyltransferase with hyperactivity and specific selectivity to lysines 9 and 27 of histone H3. J Biol Chem.

[222] Mora A, Komander D, van Aalten DMF, Alessi DR (2004). PDK1, the master regulator of AGC kinase signal transduction. Semin Cell Dev Biol.

[223] Manning BD, Cantley LC (2007). AKT/PKB signaling: navigating downstream. Cell.

[224] Ghosh R, Fatahian AN, Rouzbehani OMT (2024). Sequestosome 1 (p62) mitigates hypoxia-induced cardiac dysfunction by stabilizing hypoxia-inducible factor 1α and nuclear factor erythroid 2-related factor 2. Cardiovasc Res.

[225] Bai J, Uehara Y, Montell DJ (2000). Regulation of invasive cell behavior by taiman, a Drosophila protein related to AIB1, a steroid receptor coactivator amplified in breast cancer. Cell.

[226] List HJ, Lauritsen KJ, Reiter R, Powers C, Wellstein A, Riegel AT (2001). Ribozyme targeting demonstrates that the nuclear receptor coactivator AIB1 is a rate-limiting factor for estrogen-dependent growth of human MCF-7 breast cancer cells. J Biol Chem.

[227] York B, O'Malley BW (2010). Steroid receptor coactivator (SRC) family: masters of systems biology. J Biol Chem.

[228] Horwitz KB, Sartorius CA (2020). 90 YEARS OF PROGESTERONE: Progesterone and progesterone receptors in breast cancer: past, present, future. J Mol Endocrinol.

[229] Meena JK, Wang JH, Neill NJ (2024). MYC Induces Oncogenic Stress through RNA Decay and Ribonucleotide Catabolism in Breast Cancer. Cancer Discov.

[230] Seraj MJ, Samant RS, Verderame MF, Welch DR (2000). Functional evidence for a novel human breast carcinoma metastasis suppressor, BRMS1, encoded at chromosome 11q13. Cancer Res.

[231] Phillips KK, Welch DR, Miele ME, Lee JH, Wei LL, Weissman BE (1996). Suppression of MDA-MB-435 breast carcinoma cell metastasis following the introduction of human chromosome 11. Cancer Res.

[232] Zimmermann RC, Welch DR (2020). BRMS1: a multifunctional signaling molecule in metastasis. Cancer Metastasis Rev.

[233] Sivri NS, Tetikoğlu S, Kolayli S, Farooqi AA, Çelik Uzuner S (2024). Anti-metastatic Effects of Bee Venom and Melittin in Breast Cancer Cells by Upregulation of BRMS1 and DRG1 Genes. Chem Biol Drug Des.

[234] Hurst DR, Edmonds MD, Scott GK, Benz CC, Vaidya KS, Welch DR (2009). Breast cancer metastasis suppressor 1 up-regulates miR-146, which suppresses breast cancer metastasis. Cancer Res.

[235] Matés JM, Di Paola FJ, Campos-Sandoval JA, Mazurek S, Márquez J (2020). Therapeutic targeting of glutaminolysis as an essential strategy to combat cancer. Semin Cell Dev Biol.

[236] Yang J, Hou Y, Zhou M (2016). Twist induces epithelial-mesenchymal transition and cell motility in breast cancer via ITGB1-FAK/ILK signaling axis and its associated downstream network. Int J Biochem Cell Biol.

[237] Xu Y, Lee D-K, Feng Z (2017). Breast tumor cell-specific knockout of Twist1 inhibits cancer cell plasticity, dissemination, and lung metastasis in mice. Proc Natl Acad Sci U S A.

[238] Yang J, Mani SA, Donaher JL (2004). Twist, a master regulator of morphogenesis, plays an essential role in tumor metastasis. Cell.

[239] Eckert MA, Lwin TM, Chang AT (2011). Twist1-induced invadopodia formation promotes tumor metastasis. Cancer Cell.

[240] Zhou W, Slingerland JM (2014). Links between oestrogen receptor activation and proteolysis: relevance to hormone-regulated cancer therapy. Nat Rev Cancer.

[241] Zhang P, Gao K, Jin X (2015). Endometrial cancer-associated mutants of SPOP are defective in regulating estrogen receptor-α protein turnover. Cell Death Dis.

[242] Ge Y, Jin J, Chen G, Li J, Ye M, Jin X (2023). Endometrial cancer (EC) derived G3BP1 overexpression and mutant promote EC tumorigenesis and metastasis via SPOP/ERα axis. Cell Commun Signal.

[243] Wellbrock C, Karasarides M, Marais R (2004). The RAF proteins take centre stage. Nat Rev Mol Cell Biol.

[244] Wan PTC, Garnett MJ, Roe SM (2004). Mechanism of activation of the RAF-ERK signaling pathway by oncogenic mutations of B-RAF. Cell.

[245] Lee S-U, Maeda T (2012). POK/ZBTB proteins: an emerging family of proteins that regulate lymphoid development and function. Immunol Rev.

[246] Lim J-H (2014). Zinc finger and BTB domain-containing protein 3 is essential for the growth of cancer cells. BMB Rep.

[247] Tamura T, Yanai H, Savitsky D, Taniguchi T (2008). The IRF family transcription factors in immunity and oncogenesis. Annu Rev Immunol.

[248] Schwartz I, Vunjak M, Budroni V (2023). SPOP targets the immune transcription factor IRF1 for proteasomal degradation. Elife.

[249] Sanjo H, Kawai T, Akira S (1998). DRAKs, novel serine/threonine kinases related to death-associated protein kinase that trigger apoptosis. J Biol Chem.

[250] Park Y, Pang K, Park J (2020). Destablilization of TRAF6 by DRAK1 Suppresses Tumor Growth and Metastasis in Cervical Cancer Cells. Cancer Res.

[251] Wilbanks A, Zondlo SC, Murphy K (2001). Expression cloning of the STRL33/BONZO/TYMSTRligand reveals elements of CC, CXC, and CX3C chemokines. J Immunol.

[252] Koya J, Tanigawa T, Mizuno K (2024). Modeling NK-cell lymphoma in mice reveals its cell-of-origin and microenvironmental changes and identifies therapeutic targets. Nat Commun.

[253] Abiko K, Hamanishi J, Matsumura N, Mandai M (2023). Dynamic host immunity and PD-L1/PD-1 blockade efficacy: developments after 'IFN-γ from lymphocytes induces PD-L1 expression and promotes progression of ovarian cancer'. Br J Cancer.

[254] Webb JR, Milne K, Kroeger DR, Nelson BH (2016). PD-L1 expression is associated with tumor-infiltrating T cells and favorable prognosis in high-grade serous ovarian cancer. Gynecol Oncol.

[255] Friedman CF, Manning-Geist BL, Zhou Q (2024). Nivolumab for mismatch-repair-deficient or hypermutated gynecologic cancers: a phase 2 trial with biomarker analyses. Nat Med.

[256] Claessens LA, Vertegaal ACO (2024). SUMO proteases: from cellular functions to disease. Trends Cell Biol.

[257] Pei J, Zou D, Li L (2024). Senp7 deficiency impairs lipid droplets maturation in white adipose tissues via Plin4 deSUMOylation. J Biol Chem.

[258] Hu J, Shibata Y, Zhu P-P (2009). A class of dynamin-like GTPases involved in the generation of the tubular ER network. Cell.

[259] Hu J, Shibata Y, Voss C (2008). Membrane proteins of the endoplasmic reticulum induce high-curvature tubules. Science.

[260] Rämö O, Kumar D, Gucciardo E (2016). NOGO-A/RTN4A and NOGO-B/RTN4B are simultaneously expressed in epithelial, fibroblast and neuronal cells and maintain ER morphology. Sci Rep.

[261] Zhang D, Utsumi T, Huang H-C (2011). Reticulon 4B (Nogo-B) is a novel regulator of hepatic fibrosis. Hepatology.

[262] Marin EP, Moeckel G, Al-Lamki R (2010). Identification and regulation of reticulon 4B (Nogo-B) in renal tubular epithelial cells. Am J Pathol.

[263] Wright PL, Yu J, Di YPP (2010). Epithelial reticulon 4B (Nogo-B) is an endogenous regulator of Th2-driven lung inflammation. J Exp Med.

[264] Acevedo L, Yu J, Erdjument-Bromage H (2004). A new role for Nogo as a regulator of vascular remodeling. Nat Med.

[265] Xiao W, Zhou S, Xu H (2013). Nogo-B promotes the epithelial-mesenchymal transition in HeLa cervical cancer cells via Fibulin-5. Oncol Rep.

[266] Zhao L, Qiu Z, Yang Z (2024). Lymphatic endothelial-like cells promote glioblastoma stem cell growth through cytokine-driven cholesterol metabolism. Nat Cancer.

[267] Xiao M-Y, Li F-F, Xie P (2023). Gypenosides suppress hepatocellular carcinoma cells by blocking cholesterol biosynthesis through inhibition of MVA pathway enzyme HMGCS1. Chem Biol Interact.

[268] Feng X, Lu T, Li J (2020). The Tumor Suppressor Interferon Regulatory Factor 2 Binding Protein 2 Regulates Hippo Pathway in Liver Cancer by a Feedback Loop in Mice. Hepatology.

[269] Manjur ABMK, Lempiäinen JK, Malinen M, Palvimo JJ, Niskanen EA (2019). IRF2BP2 modulates the crosstalk between glucocorticoid and TNF signaling. J Steroid Biochem Mol Biol.

[270] Wu A, Wu Q, Deng Y (2019). Loss of VGLL4 suppresses tumor PD-L1 expression and immune evasion. EMBO J.

[271] Wen Y, Zhou X, Lu M (2019). Bclaf1 promotes angiogenesis by regulating HIF-1α transcription in hepatocellular carcinoma. Oncogene.

[272] He W, Zhang J, Liu B (2020). S119N Mutation of the E3 Ubiquitin Ligase SPOP Suppresses SLC7A1 Degradation to Regulate Hepatoblastoma Progression. Mol Ther Oncolytics.

[273] Papadopoulos V, Tsapakidis K, Riobo Del Galdo NA (2016). The Prognostic Significance of the Hedgehog Signaling Pathway in Colorectal Cancer. Clin Colorectal Cancer.

[274] Li X, Su X, Liu R (2021). HDAC inhibition potentiates anti-tumor activity of macrophages and enhances anti-PD-L1-mediated tumor suppression. Oncogene.

[275] Zhang S-L, Zhu H-Y, Zhou B-Y (2019). Histone deacetylase 6 is overexpressed and promotes tumor growth of colon cancer through regulation of the MAPK/ERK signal pathway. Onco Targets Ther.

[276] Jayachandran U, Grey H, Cook AG (2016). Nuclear factor 90 uses an ADAR2-like binding mode to recognize specific bases in dsRNA. Nucleic Acids Res.

[277] Shi L, Godfrey WR, Lin J, Zhao G, Kao PN (2007). NF90 regulates inducible IL-2 gene expression in T cells. J Exp Med.

[278] Larsen LJ, Møller LB (2020). Crosstalk of Hedgehog and mTORC1 Pathways. Cells.

[279] Mertens AE, Roovers RC, Collard JG (2003). Regulation of Tiam1-Rac signalling. FEBS Lett.

[280] Lee E-W, Seo J, Jeong M, Lee S, Song J (2012). The roles of FADD in extrinsic apoptosis and necroptosis. BMB Rep.

[281] Tourneur L, Chiocchia G (2010). FADD: a regulator of life and death. Trends Immunol.

[282] Haigis MC, Sinclair DA (2010). Mammalian sirtuins: biological insights and disease relevance. Annu Rev Pathol.

[283] Iwasaki A, Medzhitov R (2010). Regulation of adaptive immunity by the innate immune system. Science.

[284] Liu T, Wei J, Jiang C (2017). CHAF1A, the largest subunit of the chromatin assembly factor 1 complex, regulates the growth of H1299 human non-small cell lung cancer cells by inducing G0/G1 cell cycle arrest. Exp Ther Med.

[285] Yang B-Z, Liu M-Y, Chiu K-L (2024). DHX9 SUMOylation is required for the suppression of R-loop-associated genome instability. Nat Commun.

[286] Riggi N, Suvà ML, Stamenkovic I (2021). Ewing's Sarcoma. N Engl J Med.

[287] Suvarna K, Jayabal P, Ma X (2024). Ceramide-induced cleavage of GPR64 intracellular domain drives Ewing sarcoma. Cell Rep.

[288] Shi D, Tao J, Man S (2024). Structure, function, signaling pathways and clinical therapeutics: The translational potential of STAT3 as a target for cancer therapy. Biochim Biophys Acta Rev Cancer.

[289] Hu Y, Dong Z, Liu K (2024). Unraveling the complexity of STAT3 in cancer: molecular understanding and drug discovery. J Exp Clin Cancer Res.

[290] He D, Li L, Zhu G (2014). ASC-J9 suppresses renal cell carcinoma progression by targeting an androgen receptor-dependent HIF2α/VEGF signaling pathway. Cancer Res.

[291] Jiang Y, Zhang W, Kondo K (2003). Gene expression profiling in a renal cell carcinoma cell line: dissecting VHL and hypoxia-dependent pathways. Mol Cancer Res.

[292] Chi J-T, Wang Z, Nuyten DSA (2006). Gene expression programs in response to hypoxia: cell type specificity and prognostic significance in human cancers. PLoS Med.

[293] Weng Y, Xue J, Niu N (2024). SETD2 in cancer: functions, molecular mechanisms, and therapeutic regimens. Cancer Biol Med.

[294] Brockett JS, Manalo T, Zein-Sabatto H (2024). A missense SNP in the tumor suppressor SETD2 reduces H3K36me3 and mitotic spindle integrity in Drosophila. Genetics.

[295] Yu F-X, Guan K-L (2013). The Hippo pathway: regulators and regulations. Genes Dev.

[296] Carter P, Schnell U, Chaney C (2021). Deletion of Lats1/2 in adult kidney epithelia leads to renal cell carcinoma. J Clin Invest.

[297] Guo Z-Q, Zheng T, Chen B (2016). Small-Molecule Targeting of E3 Ligase Adaptor SPOP in Kidney Cancer. Cancer Cell.

[298] Dong Z, Wang Z, Guo Z-Q (2020). Structure-Activity Relationship of SPOP Inhibitors against Kidney Cancer. J Med Chem.

[299] Liu J, Ma J, Liu Y (2020). PROTACs: A novel strategy for cancer therapy. Semin Cancer Biol.

[300] Deng Z, Catlett J, Lee Y (2025). Harnessing the SPOP E3 Ubiquitin Ligase via a Bridged Proteolysis Targeting Chimera (PROTAC) Strategy for Targeted Protein Degradation. J Med Chem.

[301] Tsai JM, Nowak RP, Ebert BL, Fischer ES (2024). Targeted protein degradation: from mechanisms to clinic. Nat Rev Mol Cell Biol.

[302] Zhang L, Riley-Gillis B, Vijay P, Shen Y (2019). Acquired Resistance to BET-PROTACs (Proteolysis-Targeting Chimeras) Caused by Genomic Alterations in Core Components of E3 Ligase Complexes. Mol Cancer Ther.

[303] Han X, Zhao L, Xiang W (2019). Discovery of Highly Potent and Efficient PROTAC Degraders of Androgen Receptor (AR) by Employing Weak Binding Affinity VHL E3 Ligase Ligands. J Med Chem.

