# Supplementary material for: Challenges and opportunities for the diverse substrates of SPOP E3 ubiquitin ligase in cancer
Source: Theranostics. 2025 May 8;15(13):6111–45. doi: 10.7150/thno.113356 (PMC12159753; doi:10.7150/thno.113356)
Supplement: Supplementary file 1 — Supplementary figures and tables. [file thnov15p6111s1.pdf]

Challenges and opportunities for the diverse substrates of SPOP E3 ubiquitin ligase in cancer

Xiaojuan Yang[1,2], Jiang Zhu[1,4], Xue Tao[1,3], Fengwei Gao[1,3], Yunshi Cai[1,3], Yinghao Lv[1,3], Sinan Xie[1,3], Kunlin Xie[1,3], Tian Lan[1,3*], Junhong Han[2*], Hong Wu[1,3*]

[1]Liver Digital Transformation Research Laboratory, State Key Laboratory of Biotherapy and Cancer Center, West China Hospital, Sichuan University and Collaborative Innovation Center of Biotherapy, Chengdu, Sichuan 610041, P.R. China

[2]Department of Biotherapy, Cancer Center and State Laboratory of Biotherapy, and Frontiers Science Center for Disease-related Molecular Network, West China Hospital, Sichuan University, Chengdu, 610041, China.

[3]Liver Transplantation Center, Liver Digital Transformation Research Laboratory, State Key Laboratory of Biotherapy and Cancer Center, West China Hospital, Sichuan University and Collaborative Innovation Center of Biotherapy, Chengdu, Sichuan 610041, P.R. China

[4]Breast Center, Department of General Surgery, West China Hospital, Sichuan University, Chengdu, China

Authors' contact details: Xiao Juan Yang: y3532388972@163.com; Jiang Zhu: zhujsurgery@163.com; Xue Tao: TaoXue1107@163.com; Fengwei Gao: gaofengwei@scu.edu.cn; Yunshi Cai: caiyunshi@scu.edu.cn; Yinghao Lv: lvyinghao@scu.edu.cn; Sinan Xie: snanxie1998@163.com; Kunlin Xie: xiekun@scu.edu.cn; Tian Lan: blue_sky_land@163.com; Junhong Han: hjunhong@scu.edu.cn; Hong Wu: wuhong@scu.edu.cn

***Correspondence:**

Tian Lan: Liver Transplantation Center, Liver Digital Transformation Research Laboratory, State Key Laboratory of Biotherapy and Cancer Center, West China Hospital, Sichuan University and Collaborative Innovation Center of Biotherapy, Chengdu, Sichuan 610041, P.R. China. blue_sky_land@163.com.

Junhong Han: Department of Biotherapy, Cancer Center and State Laboratory of Biotherapy, and Frontiers Science Center for Disease-related Molecular Network, West China Hospital, Sichuan University, Chengdu, 610041, China. hjunhong@scu.edu.cn

Hong Wu: Liver Transplantation Center, Liver Digital Transformation Research Laboratory, State Key Laboratory of Biotherapy and Cancer Center, West China Hospital, Sichuan University and Collaborative Innovation Center of Biotherapy, Chengdu, Sichuan 610041, P.R. China. wuhong@scu.edu.cn.

Supplementary Table 1. Abbreviations used.

Abbreviations (A-Z)	Full names
53BP1	p53 binding protein 1
APC/C	anaphase-promoting complex/cyclosome
AR	Androgen receptor
ASCT2	Alanine serine cysteine transporter 2
ATF2	Activating transcription factor 2
ATM	Ataxia-telangiectasia mutated
BCLAF1	B cell lymphoma-2-associated transcription factor 1
BET	Bromodomain and extraterminal domain
BMI1	B-lymphoma Mo-MLV insertion region 1
BRAF	B-Raf proto-oncogene
BTB	Bric-à-brac/Tramtrack/Broad
BRD2/3/4	Bromodomain containing proteins 2/3/4
BRMS1	Breast cancer metastasis suppressor 1
CAF-1	Chromatin assembly factor-1
CAFs	Cancer-associated fibroblasts
ccRCC	Clear cell renal cell carcinoma
CDCA5	Cell division cycle associated 5
Cdc20	Cell division cycle 20

Abbreviations (A-Z)	Full names
CDK2	cyclin-dependent kinase 2
CHAF1A	Chromatin assembly factor 1 subunit A
CRC	Colorectal cancer
CRLs	Cullin–RING ligases
CRPC	Castration-resistant prostate cancer
CSCs	Cancer stem cells
CXCL16	C-X-C motif chemokine ligand 16
DDIT3	DNA damage inducible transcript 3
DDR	DNA damage response
DLBCL	Diffuse large B-cell lymphoma
DRAK1	Death-associated protein kinase-related apoptosis-inducing kinase 1
dsRNA	Double-stranded RNA
EglN2	Egl-9 family hypoxia inducible factor 2
EMT	Epithelial-mesenchymal transition
ERα	Estrogen receptor α
ERG	ETS-related gene
FADD	FAS-associated death structural domain
FASN	Fatty acid synthase
FL	Full-length
GC	Gastric cancer
GDP	Guanosine diphosphate
GEF	Guanine nucleotide exchange factor
GTP	Guanosine triphosphate
H3K9me3	Trimethylation of histone H3 at lysine 9
H3K36me3	Trimethylation of histone H3 at lysine 36

Abbreviations (A-Z)	Full names
HB	Hepatoblastoma
HCC	Hepatocellular carcinoma
HDACs	Histone deacetylases
HECT	Homology to E6AP C-terminus
HGPIN	High-grade prostatic intraepithelial neoplasia
Hh	Hedgehog
HIFα	Hypoxia-inducible factor alpha
HIPK2	Homeodomain interacting protein kinase 2
HMGCS1	3-hydroxy-3-methylglutaryl-CoA synthase 1
HMTase	Histone methyltransferase
HP1γ	Heterochromatin protein 1γ
HR	Homologous recombination
ICB	Immune checkpoint blockade
IDRs	Intrinsically disordered regions
IL-1R	Interleukin-1 receptor
INF2	Inverted formin 2
IRF2BP2	Interferon regulatory factor 2-binding protein 2
IRF1	Interferon regulatory factor 1
KC	Kidney cancer
LLPS	Liquid-liquid phase separation
LRP5	Low-density lipoprotein receptor-related protein 5
MATH	Meprin and TRAF homology
MCM3	Minichromosome maintenance complex component 3
MTDH	metadherin
NEPC	Neuroendocrine prostate cancer

Abbreviations (A-Z)	Full names
NHEJ	Nonhomologous end joining
NLS	Nuclear localization sequence
NSCLC	Non-small cell lung cancer
OGT	O-GlcNAcylated by O-GlcNAc transferase
PARP	Poly(ADP-ribose) polymerase
PCa	Prostate cancer
PcG	Polycomb group
PD-1	Programmed death receptor-1
Pdx1	Pancreatic duodenal homeobox 1
PDK1	3-phosphoinositide-dependent kinase 1
PD-L1	Programmed death-ligand 1
PIN	Prostatic intraepithelial neoplasia
PIP3	PtdIns(3,4,5)P3
PR	Progesterone receptor
PRC1	Polycomb repressive complex 1
PrLZ	Prostate leucine zipper
RAF	Rapidly accelerated fibrosarcoma
RBP1	Retinoblastoma binding protein 1
RBR	RING homology-in-between-RING
RCC	Renal cell carcinoma
RING	Really interesting new gene
ROS	Reactive oxygen species
RTKi	Receptor tyrosine kinase inhibitor
RTN-4B	Reticulon-4B
SBC	SPOP-binding consensus
SENP7	Sentrin/SUMO-specific protease 7

Abbreviations (A-Z)	Full names
SGK3	Serum- and glucocorticoid-regulated kinase-3
SGOC	Serine–Glycine–One-Carbon
SG	Stress granule
SHH	Sonic hedgehog
shRNA	Short hairpin RNA
siRNAs	Small interfering RNAs
SLC7A1	Solute carrier family 7 member 1
SPOP	Speckle-type POZ protein
SQSTM1	Sequestosome-1
SRC3	Steroid receptor coactivator 3
STAT3	Signal transducers and transcriptional activators 3
SRF	Serum response factor
STK17A	Serine/threonine protein kinase 17A
SUMO	Small ubiquitin-related modifier
TILs	Tumor-infiltrating lymphocytes
TIAM1	T lymphoma invasion and metastasis 1
TLR	Toll-like receptor
TME	Tumor microenvironment
TPD52	Tumor protein D52
TRIM24	Tripartite motif containing 24
TWIST1	Twist family BHLH transcription factor 1
UPS	Ubiquitin-proteasome system
WT	Wild-type
ZBTB3	Zinc finger and BTB domain-containing protein 3
